# Non-energetic Formation of Ethanol via CCH Reaction with Interstellar H_2_O Ices. A Computational Chemistry Study

**DOI:** 10.1021/acsearthspacechem.1c00369

**Published:** 2022-03-07

**Authors:** Jessica Perrero, Joan Enrique-Romero, Berta Martínez-Bachs, Cecilia Ceccarelli, Nadia Balucani, Piero Ugliengo, Albert Rimola

**Affiliations:** † Departament de Química, Universitat Autònoma de Barcelona, Bellaterra, 08193 Catalonia, Spain; ‡ Dipartimento di Chimica and Nanostructured Interfaces and Surfaces (NIS) Centre, Università degli Studi di Torino, via P. Giuria 7, 10125 Torino, Italy; ¶ Univ. Grenoble Alpes, CNRS, Institut de Planétologie et d’Astrophysique de Grenoble (IPAG), 38000 Grenoble, France; § Dipartimento di Chimica, Biologia e Biotecnologie, Università di Perugia, Via Elce di Sotto 8, 06123 Perugia, Italy; ∥ Osservatorio Astrosico di Arcetri, Largo E. Fermi 5, 50125 Firenze, Italy

**Keywords:** interstellar medium, astrochemistry, DFT, iCOMs, grains

## Abstract

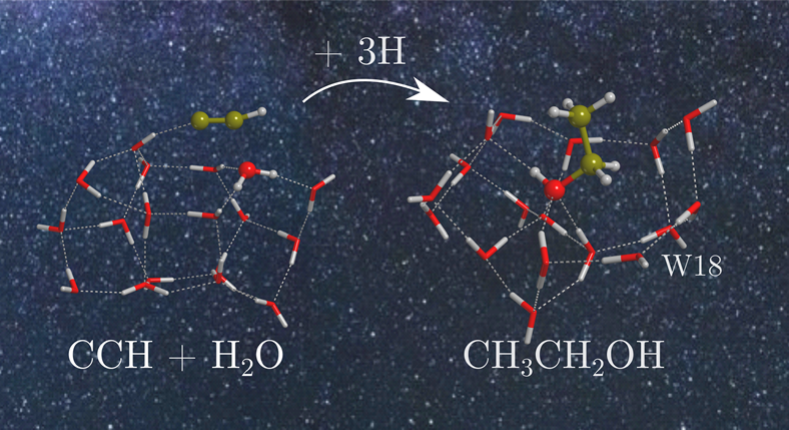

Ethanol (CH_3_CH_2_OH) is a relatively common molecule, often found in star-forming regions. Recent studies suggest that it could be a parent molecule of several so-called interstellar complex organic molecules (iCOMs). However, the formation route of this species remains under debate. In the present work, we study the formation of ethanol through the reaction of CCH with one H_2_O molecule belonging to the ice as a test case to investigate the viability of chemical reactions based on a “radical + ice component” scheme as an alternative mechanism for the synthesis of iCOMs, beyond the usual radical–radical coupling. This has been done by means of DFT calculations adopting two clusters of 18 and 33 water molecules as ice models. Results indicate that CH_3_CH_2_OH can potentially be formed by this proposed reaction mechanism. The reaction of CCH with H_2_O on the water ice clusters can be barrierless (because of the help of boundary icy water molecules acting as proton-transfer assistants), leading to the formation of vinyl alcohol precursors (H_2_CCOH and CHCHOH). Subsequent hydrogenation of vinyl alcohol yielding ethanol is the only step presenting a low activation energy barrier. We finally discuss the astrophysical implications of these findings.

## Introduction

Interstellar complex organic molecules (iCOMs) are compounds between 6 and 12/13 atoms, in which at least one is carbon, conferring the organic character.^1−3^ These molecules are important because (i) they can be considered as the simplest organic compounds that are synthesized in space (hence representing the dawn of organic chemistry) and (ii) they are the precursors of more complex organic molecules, which can be of biological relevance, such as amino acids, nucleobases, and sugars. Indeed, there is robust evidence that the iCOMs formed in the interstellar medium (ISM) were inherited by small objects of the solar system^4−8^ (*e.g.*, carbonaceous chondrites), which upon alteration mechanisms (*e.g.*, hydrothermal) can be converted into more evolved organic species,^9−12^ thereby providing a potential contribution to the emergence of life on Earth.

The first detection of iCOMs took place in 1971 in massive star formation regions,^13^ but we had to wait for the beginning of this century for detections in regions that could eventually evolve in solar-like planetary systems.^6,14^ Currently, complex organic molecules have been detected in different astrophysical environments such as prestellar cores, protostellar outflows, protoplanetary disks, and even external galaxies.^3,8,15−18^

Two different prevailing paradigms have been postulated for the formation of iCOMs: one based on gas-phase reactions^19−24^ and the other based on radical–radical couplings occurring on grain surfaces,^25,26^ although other paradigms have been postulated, like those based on the condensation of atomic C,^27,28^ excited O atom insertion,^29,30^ or the formation of HCO on surfaces as a parent precursor of other iCOMs.^31,32^ Both prevailing reaction models have the same initial step: formation of hydrogenated species (*e.g.*, CH_3_OH, NH_3_, H_2_CO, and CH_4_) by H-addition on icy grain surfaces in the cold prestellar phase. In the gas-phase paradigm, the process follows with the desorption of these species, either thermally when the grain surface reaches *ca.* 100 K like in hot cores/corinos, nonthermally by photodesorption because of UV incidence on the grains,^33−35^ by chemical desorption once they are formed,^36−41^ or induced by cosmic rays (CRs).^42−45^ In the gas phase they react with other gaseous species forming iCOMs. In the on-grain paradigm, the hydrogenated species act as parent species of molecular radicals (*e.g.*, CH_3_, HCO, and NH_2_) formed by the irradiation of UV photons and/or energetic ions, partial hydrogenation, and H-abstraction reactions^2,25,26,46−50^ during the cold preprotostellar stage. Later on, these radicals, because of the warm up of the protostar surroundings (*ca.* 30 K), can diffuse on the icy surfaces and encounter each other to couple and form the iCOMs.

These two reaction paradigms have been largely used in combined observational/astrochemical modeling studies to rationalize the presence of iCOMs in the given sources (*e.g.*, see refs (3 and 51−53)). Additionally, they have also been assumed to study the formation of iCOMs by means of quantum chemical simulations.^54−57^ By compiling all the available works, it seems that both paradigms are necessary to explain the presence, distribution, and abundance of the wide and rich organic chemistry in the ISM. Thus, knowing whether the formation of an iCOM is dominated by surface or gas-phase chemistry is a case-by-case problem. It depends on the nature of the iCOMs, and each one has to be addressed as a particular case.

With the different quantum chemical studies addressing on-grain radical–radical couplings, some drawbacks of this paradigm have been identified. One is that the chance for the coupling is a delicate trade-off between the diffusion of the radicals and their desorption. That is, the temperature increase that enables the radical diffusion must be lower than their desorption temperatures. This can give rise to a small temperature window in which the coupling can take place, because this *coupling temperature window* is defined by the lowest diffusion temperature and the lower desorption temperature between the two radicals. For instance, in acetaldehyde formation by the coupling of CH_3_ and HCO, the coupling temperature window was found to be between the temperatures at which CH_3_ becomes mobile on the surface (between 9 and 15 K, depending on the adopted diffusion barrier) and the temperature at which the methyl radical would desorb (30 K).^55^ Another drawback is that radical–radical couplings are often assumed to be barrierless because they are driven by the coupling of the opposite electronic spins of the radicals. However, the reactions can exhibit energy barriers because the radicals, to proceed with the coupling, need to break the interactions with the icy surfaces. Obviously, as a rule of thumb, the stronger the interaction, the higher the energy barrier. A third limitation of this paradigm is that radical–radical couplings can have competitive channels inhibiting the efficiency of the iCOM formation. The competitive reactions are based on H-abstractions from one radical to the other. For instance, the CH_3_ + HCO coupling can give CH_3_CHO (acetaldehyde as iCOM) but also CH_4_ + CO (the H-abstraction product).

In a previous work by some of us,^58^ an alternative on-grain mechanism different from the radical–radical coupling was proposed. It is based on the reaction of a radical (coming from the gas phase or generated by UV irradiation) with neutral, entire components of the ice, *i.e.*, H_2_O and CO as the most abundant components. In that work, this mechanism was tested to form formamide (NH_2_CHO) through the reaction of the radical CN with a water molecule belonging to the ice, *i.e.*, CN + H_2_O(ice) → NH_2_CO, in which the resulting species can be easily hydrogenated to form formamide. This alternative mechanism overcomes the problems of (i) the coupling temperature window (the diffusion of the radical is not needed because it reacts with an abundant ice component, thus increasing the chance of the encountering among the two reactants) and (ii) the competitive channel (they *a priori* do not present any other possible reaction). However, they present energy barriers because the reaction is between a radical and a neutral closed-shell species.

The present work aims to investigate this alternative on-grain mechanism by simulating with quantum chemical computations the reactivity of the CCH radical with a water molecule of the ice. The goal is to study the possibility of forming ethanol (EtOH), with the formation of vinyl alcohol (VA) as intermediate species, through the following reactions:
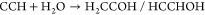
1
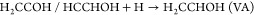
2
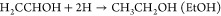
3We consider this path toward ethanol because the electronic structure of CCH is isoelectronic with CN, hence exhibiting a similar reactivity with water as shown in Rimola *et al*.^58^

CCH was one of the first interstellar polyatomic molecules to be detected.^59^ It is a fundamental and common carbon chain (in fact, the simplest) species in the ISM. It is found in regions near UV sources, the so-called photon dominated regions (PDRs) (*e.g.*, see refs (60 and 61)); in diffuse and translucent clouds (*e.g.*, see refs (62 and 63)); in protostellar objects;^64,65^ and in the cavities of protostellar outflows.^66,67^ In addition, CCH has been detected in protoplanetary disks^30,68−71^ and external galaxies (*e.g.*, see ref (72)) probably in their PDR regions/skins. CCH is also abundant toward the lukewarm (≤40 K) envelopes of the so-called warm carbon chain chemistry (WCCC) objects, which are young class 0/I sources characterized by higher abundances of carbon chains and lower abundances of iCOMs than those observed in hot corinos (see for example ref. (65)). CCH appears at scales of a few thousand AU around the protostellar center at densities of some 10^6^ cm^–3^ (*e.g.*, see ref (73)). Particularly relevant to this work, CCH is also relatively abundant in cold molecular clouds (*e.g.*, see refs (74 and 75)) and prestellar cores.^76^ Typical CCH column densities range from 10^12^–10^15^ cm^–2^,^61,77^ with the greatest values in PDRs.^61^ In molecular clouds, the CCH column density is around 10^14^ cm^–2^, equivalent to abundances of ∼10^–9^–10^–8^.^74,75^ Similar abundances are found in prestellar cores, as CCH probably resides in the least depleted zone, similar to the molecular clouds.^76,78,79^ In summary, CCH is abundant (∼10^–9^–10^–8^) in cold (≤20 K) regions where the interstellar dust grains are enveloped by icy mantles. Thus, it is worth investigating the possibility that it interacts with the water molecules of the ice to form ethanol, following the path described above (reactions 1–3).

The reaction of CCH + H_2_O has been studied as a gas-phase process at high temperatures, both experimentally and theoretically. Seminal experiments by Van Look and Peeters^80^ suggested that the outcome of the reaction was not the result of a direct H-abstraction forming HCCH + OH, and hence, the authors proposed a mechanism based on, first, an association between the two reactive partners, forming H_2_C=CHOH or HC=CHOH, followed by an elimination giving rise to H_2_CCO + H and/or HCCH + OH. Subsequent theoretical calculations,^81^ however, indicated that among the different reactive channels, the direct H-abstraction was the most kinetically favorable one, in detriment to the association–elimination mechanism. Investigations regarding this reaction ended with a combined experimental/theoretical study carried out by some of the first paper’s authors, concluding that the H-abstraction producing HCCH + OH is indeed the most facile chemical reaction.^82^

In contrast, to the best of our knowledge, no experimental works exist on the reactivity of CCH with water ices. However, the addition of OH radicals to C_2_H_2_ ices (isoelectronic with CCH + H_2_O) at temperatures below 20 K, followed by H-additions, has been studied by several authors, resulting in the formation of several products, among them vinyl alcohol and ethanol. This reactivity usually involves an energetic preprocessing of the ice analogues (*i.e.*, irradiation with ions, electrons, and photons) to generate the OH radicals. Among these works, we can find (1) ion radiolysis experiments (MeV protons) that generate mainly CO, CO_2_, methanol and ethanol;^83^ (2) MeV protons and far UV photon irradiation that yield vinyl alcohol formation;^84^ (3) UV irradiation and proton radiolysis of the ices that form vinyl alcohol, acetaldehyde, ketene, and ethanol;^85^ (4) ice irradiation with extreme UV photons that leads to the formation of some iCOMs like ethane, methanol, and ethanol, together with some simpler species (*e.g.*, CO, CO_2_, and methane);^86^ and (5) radiolysis of the C_2_H_2_:H_2_O ices with less energetic protons (200 keV) at 17 K that produce several iCOMs, like vinyl alcohol, acetaldehyde, ketene and ethanol, and some other species, such as C_2_H_4_, C_2_H_6_, C_4_H_2_, and C_4_H_4_.^87^ In this later work, it was proposed that once vinyl alcohol is formed by the attack of an OH radical to C_2_H_2_, two possible situations may take place: either an intramolecular isomerization step forming acetaldehyde or successive H-additions on vinyl alcohol to form ethanol. More recently, nonenergetic processes have also been explored, in which C_2_H_2_:O_2_ ices exposed to H atoms at 10 K produce most of the products found in Chuang **et al.**,^87^ including acetaldehyde, vinyl alcohol, ketene, and ethanol. Other experiments indicate that vinyl alcohol and acetaldehyde can also be formed through other chemical reactions. That is, the O addition to C_2_H_2_ mainly results in ketene formation,^30,85,88,89^ while the O addition to more saturated hydrocarbons (acetylene, ethane, and ethylene) leads to the formation of different iCOMs (ketene, ethanol and acetaldehyde, and acetaldehyde and ethylene oxide, respectively).^30,90^

Ethanol formation has recently received much attention because it has been postulated to be a parent molecule through which other iCOMs can be formed by different gas-phase reactions, such as acetaldehyde, glycolaldehyde, formic acid, acetic acid, and formaldehyde (the so-called genealogical tree of ethanol; see refs (23, 24, and 91)). Because of this significance, this work focuses on the formation of this ancestor molecule following reactions 1–3 on water ice surfaces by means of quantum chemical simulations to determine if they are energetically feasible.

## Methodology

All the calculations were based on density functional theory (DFT) and run with the Gaussian16 software package.^92^ Geometry optimizations and frequency calculations were all performed by combining the DFT methods with the Pople-based 6-311++G(d,p) basis set.^93,94^ These energies were subsequently refined at the 6-311++G(2df,2pd)^95^ level by performing single-point energy calculations on the optimized geometries. In order to identify the DFT method that better describes our systems (and hence to use it for the reaction simulations on the water ice surface models), we carried out a preliminary benchmarking study using the CCH + H_2_O and CH_2_CHOH + H gas-phase reactions as models. Five different hybrid DFT methods were used, which were corrected with Grimme’s D3 term or, if possible, with the D3(BJ) version^96−98^ to account for dispersion interactions. The tested DFT-D methods were BHLYP-D3(BJ),^99,100^ M062X-D3,^101^ MPWB1K-D3(BJ),^102^ PW6B95-D3(BJ),^103^ and ωB97X-D3.^104^ By comparing the results with those obtained with single energy points at the CCSD(T)/aug-cc-pVTZ^105^ level of theory, known as the “gold-standard” in quantum chemistry, we found that the ωB97X-D3 method showed the best performance when modeling the water addition to CCH, while MPWB1K-D3(BJ) described better the hydrogenation of CH_2_CHOH (see Results). Accordingly, the CCH + H_2_O and the CH_2_CHOH + H reactions on the water ice cluster models were computed at the ωB97X-D3/6-311++G(2df,2pd)//ωB97X-D3/6-311++G(d,p) and MPWB1K-D3(BJ)/6-311++G(2df,2pd)//MPWB1K-D3(BJ)/6-311++G(d,p) theory levels, respectively.

All the stationary points of the potential energy surfaces (PESs) were characterized by their analytical calculation of the harmonic frequencies as minima (reactants, products, and intermediates) and saddle points (transition states). When needed, intrinsic reaction coordinate (IRC) calculations at the level of theory adopted in the geometry optimizations were carried out to ensure that a given transition state actually connects with the corresponding minima. Thermochemical corrections to the potential energy values were carried out using the standard rigid rotor/harmonic oscillator formulas to compute the zero-point energy (ZPE) corrections.^106^ Because the systems are open-shell in nature, calculations were performed within the unrestricted formalism.

We additionally calculated the tunnelling crossover temperatures, *i.e.*, the temperature below which quantum tunnelling becomes the main mechanism for trespassing the potential energy barriers. To this end, we used eq 4,^107^ where Δ*H*^‡^ is the ZPE-corrected barrier height, ω^‡^ is the frequency associated with the TS, and *k*_B_ and *ℏ* are the Boltzmann’s and reduced Planck’s constants. This temperature indicates what reactions may actually have an important role at low temperatures despite having an activation barrier.
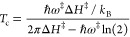
4

**Figure 1:**
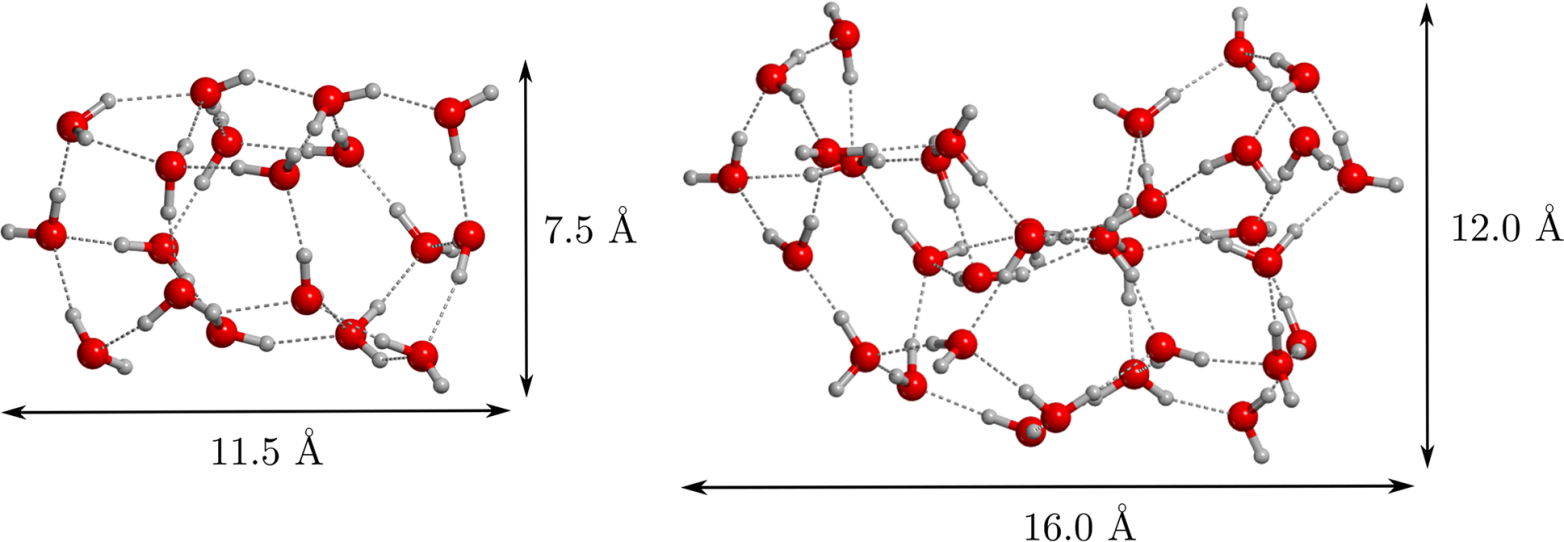
Structures of the 18 and 33 water molecule clusters, optimized at the ωB97X-D3/6-311++G(d,p) level of theory.

The surfaces of amorphous solid water (ASW) ice coating interstellar grains were simulated by two different cluster models: one consisting of 18 water molecules and the other consisiting of 33 water molecules (hereafter termed W18 and W33; Figure 1). While the former represents a compact, flat water ice surface, the latter presents a cavity structure of about 6 Å. For more details, we refer the reader to refs (54, 58, and 108).

For the calculation of the binding energies (BEs) of CCH interacting with the H_2_O ice surface models (*i.e.*, CCH/surf complexes), we adopted the same electronic structure methodology as for reactivity, namely, for each CCH/surf complex and corresponding isolated components, geometry optimizations and frequency calculations (and hence ZPE corrections) were computed at the ωB97X-D3/6-311++G(d,p) level, followed by single-point energy calculations at the improved ωB97X-D3/6-311++G(2df,2pd) level. Basis set superposition error (BSSE) was corrected following the Boys and Bernardi counterpoise method. The final, corrected, adsorption energy (Δ*E*_ads_^CP^) was calculated as
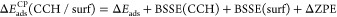
5where Δ*E*_ads_ = *E*(CCH/surf) – *E*(CCH) – *E*(surf) refers to the BSSE-noncorrected adsorption energy. Note that we used the same sign convention as in ref (54); namely, the adsorption energy is the negative of the binding energy: Δ*E*_ads_^CP^ = −BE.

## Results

### Benchmarking Study

**Figure 2:**
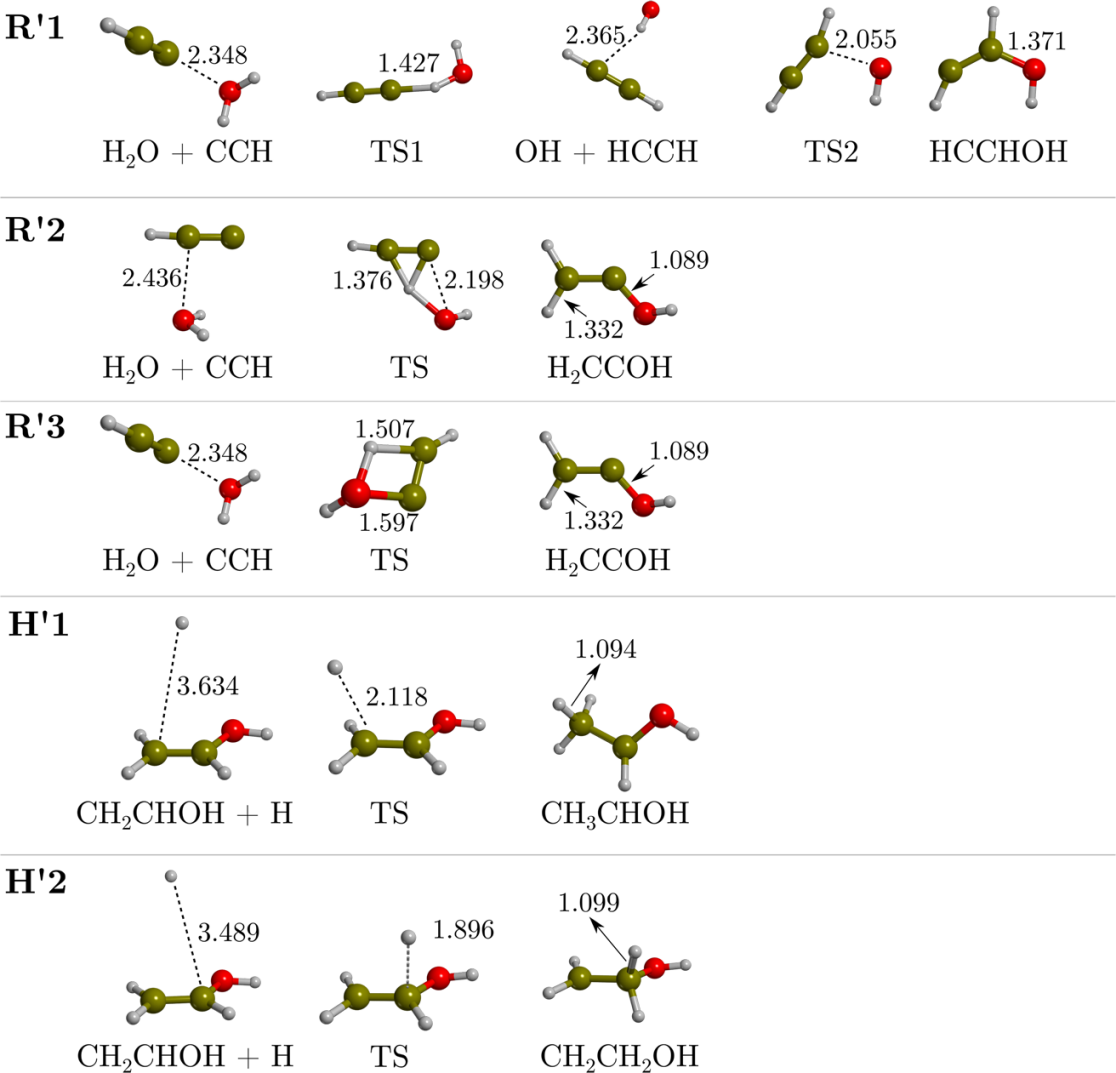
Stationary points identified in the benchmarking study. Reaction pathways **R′1**, **R′2**, and **R′3** refer to gas-phase CCH + H_2_O reaction and are optimized at ωB97X-D3. **H′1** and **H′2** correspond to the two hydrogenation pathways of vinyl alcohol, optimized at MPWB1K-D3(BJ). Distances are in Å.

As mentioned above, we carried out a preliminary benchmarking analysis for the reactivity using two gas-phase model reactions, CCH + H_2_O and CH_2_CHOH + H, to find the DFT method that describes better the reaction properties. For CCH + H_2_O, we found three possible reaction pathways, labeled as **R′1**, **R′2**, and **R′3**, the stationary points of which are shown in Figure 2.

**R′1** follows a stepwise mechanism: the first step involves the formation of acetylene (HCCH) and the hydroxyl radical (OH) as intermediate species, and the second step consists of the condensation of these intermediates to form the HCCHOH radical. In contrast, both **R′2** and **R′3** adopt a concerted mechanism. These two reactions involve a C–O bond formation followed by a H-transfer to the other C atom to form the H_2_CCOH radical (isomer of HCCHOH, the product of **R′1**). The difference between **R′2** and **R′3** is that, in the former, the H-transfer comes first, followed then by a spontaneous C–O bond formation, while in the latter, the C–O bond formation and the proton transfer evolve in a synchronized way.

The computed energetics of these reaction pathways using the different quantum chemical methods are shown in Table 1. The optimized geometries of the stationary points are available in the Supporting Information. As can be seen, the overall best functional for these reactions of water addition to CCH is ωB97X-D3, with an average unsigned error of ∼5% in the energy barriers and ∼6% in the whole energetics. The performance of the other functionals is, from better to worse, MPWB1K-D3(BJ), PW6B95-D3(BJ), BHLYP-D3(BJ), and M062X-D3.

**Table 1:** Relative Energies (in kJ mol^–1^) of the Stationary Points Involved in the Reaction Pathways Found for the Gas-Phase CCH + H_2_O Reaction Model (**R′1**, **R′2**, and **R′3**) and for the Gas-Phase Hydrogenation (**H′1** and **H′2**) for All the DFT-D Methods and the CCSD(T) Method Used for the Benchmarking Study^a^

reaction	step	BHLYP-D3(BJ)	M062X-D3	MPWB1K-D3(BJ)	PW6B95-D3(BJ)	ωB97X-D3	CCSD(T)
R′1	H_2_O + CCH	0.0	0.0	0.0	0.0	0.0	0.0
	TS1	28.5	8.9	25.6	19.6	29.0	29.1
	OH + HCCH	–91.6	–57.0	–72.5	–63.0	–62.3	–69.0
	TS2	–68.5	–62.8	–60.2	–61.1	–53.5	–51.5
	HCCHOH	–217.0	–222.0	–217.7	–203.6	–205.9	–193.8
TS1 % R′1		2.1	69.5	11.8	32.6	0.4	
TS2 % R′1		33.2	22.1	17.0	18.8	4.0	
Avg % R′1		20.0	30.9	11.5	16.3	5.1	
R′2	H_2_O + CCH	0.0	0.0	0.0	0.0	0.0	0.0
	TS1	117.4	107.5	96.4	90.8	108.0	99.5
	H_2_CCOH	–249.3	–246.8	–266.3	–253.2	–247.8	–241.1
TS % R′2		18.0	8.1	3.1	8.7	8.5	
Avg % R′2		10.7	5.2	6.8	6.9	5.6	
R′3	H_2_O + CCH	0.0	0.0	0.0	0.0	0.0	0.0
	TS	120.3	87.7	98.6	123.5	113.0	105.4
	H_2_CCOH	–240.3	–244.8	–241.9	–228.8	–229.7	–214.1
TS % R′3		16.7	16.8	6.5	17.1	7.2	
Avg % R′3		14.5	15.6	9.7	12.0	7.2	
Global Avg %		15.1	17.2	9.3	11.7	6.0	
H′1	CH_2_CHOH + H	0.0	0.0	0.0	0.0	0.0	0.0
	TS	0.3		4.1	2.3	11.4	6.5
	CH_3_CHOH	–192.6		–183.3	–177.1	–188.1	–176.0
TS % H′1		116.4		43.6	78.5	93.0	
Avg % H′1		62.9		23.9	39.6	49.9	
H′2	CH_2_CHOH + H	0.0	0.0	0.0	0.0	0.0	0.0
	TS	8.0		14.2	12.5	21.6	15.9
	CH_2_CH_2_OH	–157.9		–143.0	–177.1	–149.0	–142.0
TS % H′2		54.2		11.9	23.7	38.5	
Avg % H′2		32.7		6.3	24.1	21.7	
Global Avg %		47.8		15.1	31.8	35.8	

a See Methodology for more details. Rows indicated as “TS % RX” show the unsigned error (in percent) relative to the predicted energy of the TS. Rows indicated as “Avg % RX” show the averaged unsigned errors (in percent and accounting for all the stationary points) of each DFT-D method with respect to the CCSD(T)//ωB97X-D3 for R′1, R′2, and R′3 and CCDS(T)//MPWB1K-D3(BJ) for H′1 and H′2 reference values. The last row indicates the global averaged unsigned error (in percent).

For the hydrogenation steps (reactions 2 and 3), we considered only the hydrogenation of vinyl alcohol, namely, CH_2_CHOH + H, because it is the unique step that can exhibit an energy barrier due to involving a closed-shell molecule (vinyl alcohol) with a radical (H atom). In contrast, the other steps consist of the hydrogenation of radical species, which are barrierless processes because of the spontaneous spin–spin coupling. For this H-addition reaction, we found two possible pathways (labeled as **H′1** and **H′2** in Figure 2), leading to two different products (CH_3_CHOH and CH_2_CH_2_OH, respectively), depending on which C atom the H addition takes place. **H′1** and **H′2** share a similar mechanism, in which the H atom in the reactant structures is located at *ca.* 3.5 Å from the C atom with which it will react. Results show that path **H′1** is more favorable than **H′2**, both for the stability of the product and for presenting a lower activation energy barrier (see Table 1). This is because the **H′1** product (CH_3_CHOH) exhibits a better delocalization of the unpaired electron with respect to the **H′2** product (CH_2_CH_2_OH). Among the used DFT methods, the functional with the smallest average unsigned error compared to CCSD(T) single-point energy calculations is MPWB1K-D3(BJ). The performance of the other functionals, from better to worse, is PW6B95-D3(BJ), ωB97X-D3, and BHLYP-D3(BJ). M062X-D3 was discarded for convergence problems of the reactant structures. Therefore, according to this benchmarking study, the ωB97X-D3 DFT method has been chosen to simulate the addition of water to CCH on the W18 and W33 cluster models, and MPWB1K-D3(BJ) has been adopted for the hydrogenation step of vinyl alcohol.

### Adsorption of CCH on Water Ice and Binding Energies

**Figure 3:**
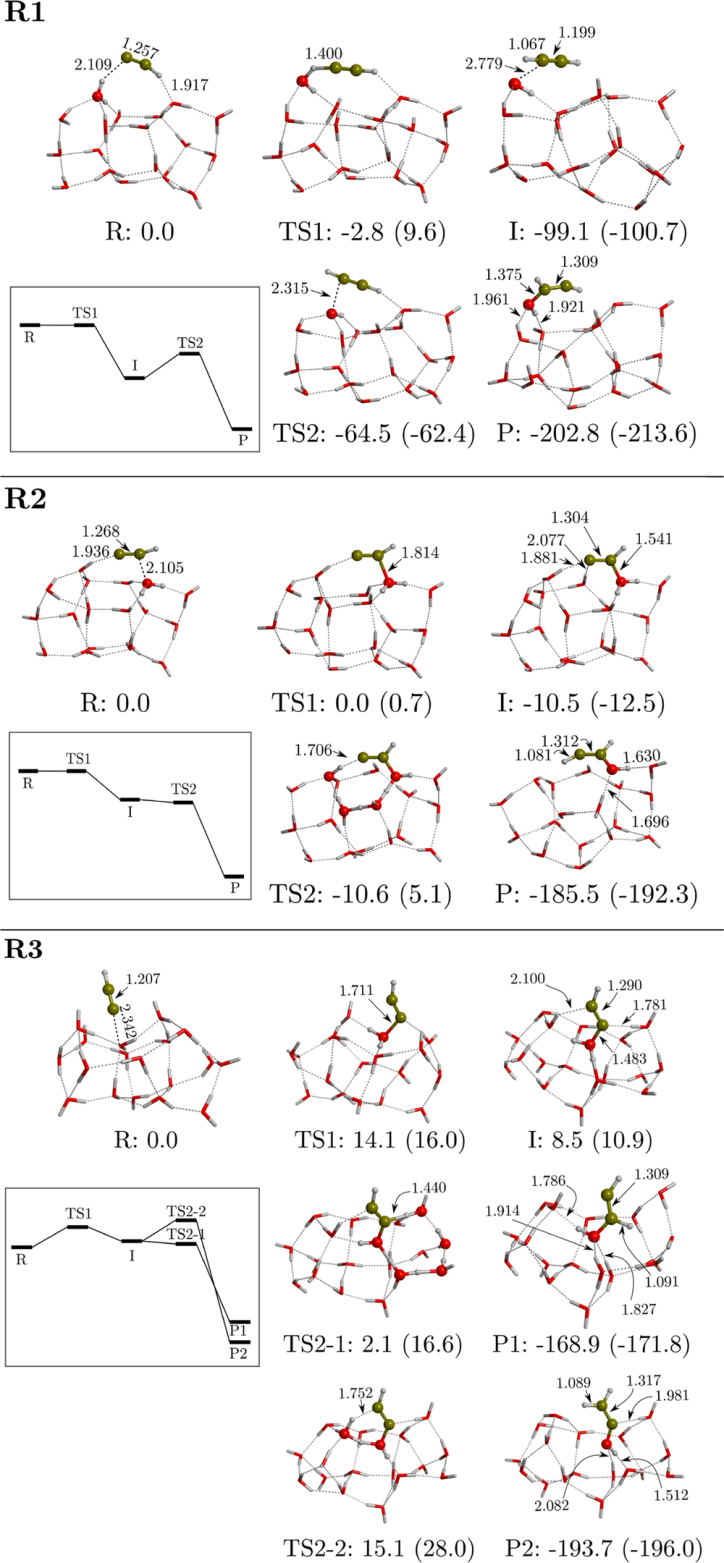
Computed potential energy surfaces (PESs) of the reactions **R1-3** between CCH and the W18 ASW ice cluster model. Bare energy values correspond to relative ZPE-corrected values, while values in parentheses correspond to those missing this correction. The miniature panels sketch the ZPE-corrected PESs. Energies are in kJ mol^–1^, and distances are in Å.

**Figure 4:**
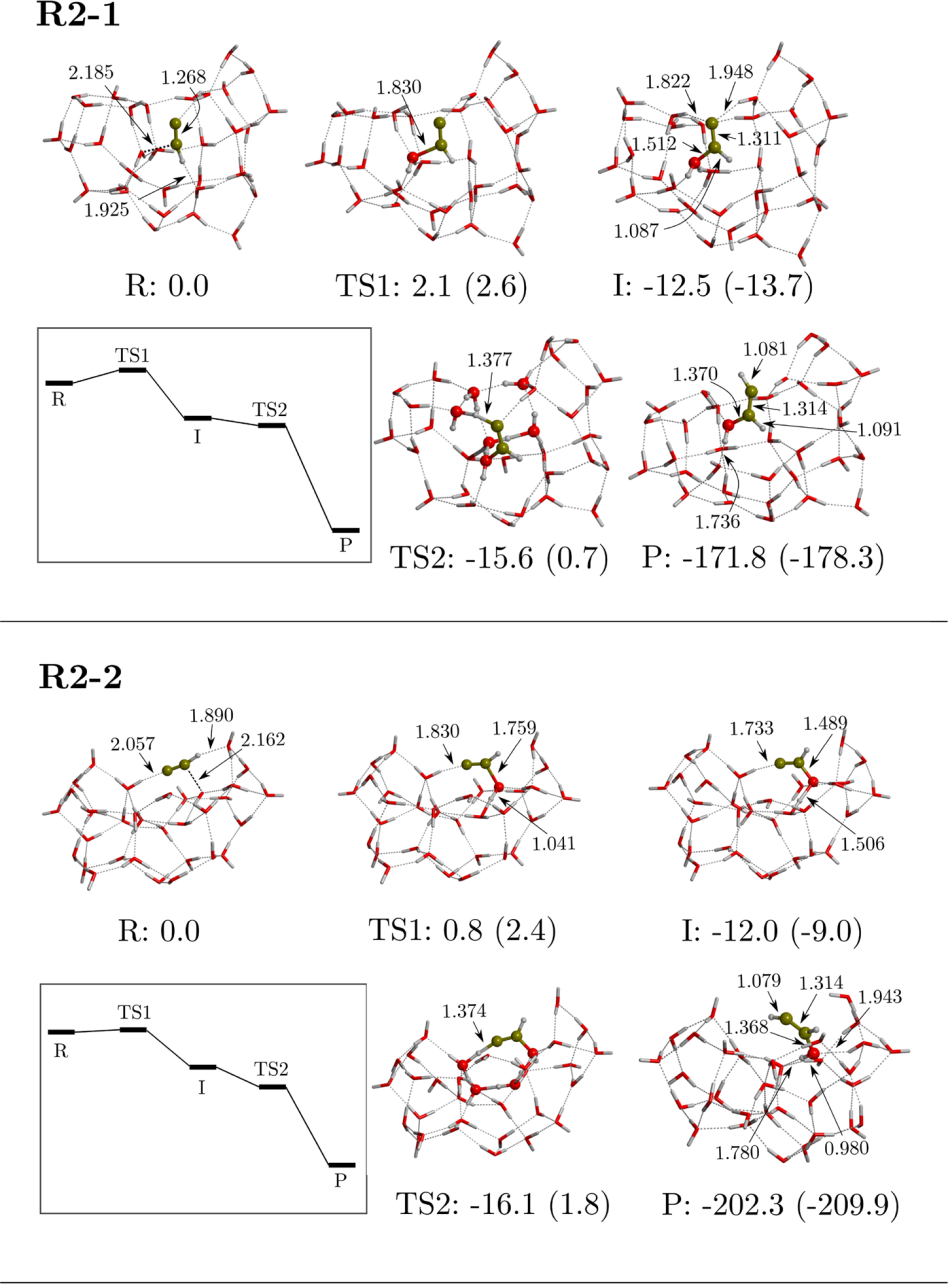
Computed potential energy surfaces (PESs) of the reactions **R2-1** and **R2-2** between CCH and W33 ASW ice cluster model. Bare energy values correspond to relative ZPE-corrected values, while values in parentheses correspond to those missing this correction. The miniature panels sketch the ZPE-corrected PESs. Energies are in kJ mol^–1^, and distances are in Å.

The complexes formed when CCH adsorbs on W18 and W33 are shown in the R structures of Figures 3 and 4, respectively. Table 2 reports the computed binding energies and the different contributions, as detailed in Methodology.

**Table 2:** Binding Energy (BE) Values (in kJ mol^–1^) of CCH on the W18 and W33 Cluster Models According to the Computed Complexes Shown in Figures 3 and 4 (the R Structures of the Reactions (Rx) **R1**, **R2**, **R3**, **R2-1**, and **R2-2**)^a^

surface	Rx	Δ*E*_ads_	Δ*E*_disp_	ΔZPE	ΔBSSE	BE
W18	R1	–21.2	–3.3	–0.6	1.4	23.7
	R2	–32.8	–10.1	3.0	2.1	37.9
	R3	–32.2	–22.1	2.2	2.2	49.9
W33	R2-1	–97.1	–7.2	11.2	3.3	89.7
	R2-2	–81.6	–19.0	11.2	3.5	86.0

a The contributions from the pure potential energy values (Δ*E*_ads_), dispersion corrections (Δ*E*_disp_), zero-point energy corrections (ΔZPE), and BSSE corrections (ΔBSSE) are also shown.

In most of the cases, a nonclassical hemibond between the CCH species and an H_2_O of the ice is established because of the formation of a two-center, three-electron system between the unpaired electron of CCH and a lone pair of H_2_O. This interaction is highlighted by the computed spin density values and maps, which clearly indicate a delocalization of the unpaired electron between the two centers (see data in the Supporting Information). The distances of these hemibonds vary between 2.1 and 2.3 Å (see the R structures of the **R2**, **R3**, **R2-1**, and **R2-2** sequences of Figures 3 and 4). The only exception not presenting a hemibonded complex is the structure R of **R1** (Figure 3), in which classical, weak hydrogen bond (H-bond) interactions are established between CCH and the W18 ice model. Interestingly, any attempt to find a pure H-bonded complex (*i.e.*, without any hemibonded interaction) on W33 failed, with the geometry optimizations collapsing into the hemibonded structures. This reinforces the idea that the more surface water molecules, the higher the possibility to form hemibonded complexes, although most of the hemibonded complexes also present H-bond interactions. This is because the outermost water molecules of the cluster are unsaturated in terms of H-bonds, presenting H- and O-dangling atoms ready to establish H-bond and hemibonded interactions, respectively. Remarkably, hemibonded systems also form in the reactant structures of the gas-phase reactions (see above). However, these gas-phase systems present a lower hemibonded character than the complexes on W18 and W33 because the spin is less delocalized between the two centers (see spin density values and maps in the Supporting Information).

As expected, hemibonded complexes present BE values larger than those of H-bonded ones (see Table 2). The difference in the BEs between the complexes shown in **R2** and **R3** (*i.e.*, on W18) arises from the orbital occupied by the CCH unpaired electron. In the former, the unpaired electron belongs to the π system of the CCH (the spin density is shared between the two C atoms, and the linearity of CCH becomes broken upon hemibond formation), while in the latter it belongs to a σ orbital of the C-end of CCH, in both cases interacting with a lone pair of H_2_O to form the hemibond. As the orbital overlap is more efficient in σ orbitals than in π orbitals, the latter complex presents a larger BE than the former (49.9 and 37.9 kJ mol^–1^, respectively). On W33, both hemibonded complexes have similar BE values (89.7 and 86.0 kJ mol^–1^) because in both systems the unpaired electron occupies a π orbital. Remarkably, the values on W33 are notably higher than those on W18, and we investigated what might be the cause. We checked for a correlation between the BE and the number of water molecules forming the cluster, carving several structures from our W33 model, that is, by removing water molecules from the edges of the model in order to build a set of water clusters with decreasing dimensions (and blocking some O atoms of the cluster to prevent the cavity from collapsing). By proceeding inthis way, we found evidence that the increasing number of interactions between CCH and the ice, together with the cooperativity of H-bonds, is responsible for the increment of the BE. Data of this analysis are provided in the Supporting Information.

Finally, it is worth mentioning that we performed a preliminary benchmarking study on the binding energies of the dimeric CCH/H_2_O system. Results (available in the Supporting Information) indicate that while the H-bonded dimer is very well described by most of the DFT methods, this is not the case for the hemibonded one. However, for the particular case of ωB97X-D3, the computed binding energies are somewhat overestimated, belonging to the group of the functionals that better compare with CCSD(T). Accordingly, the computed binding energies of the hemibonded complexes arising from CCH in interaction with W18 and W33 should be considered overestimated by some amount. Despite this drawback, we would like to stress that the main scope of the work is on the reactivity between CCH and H_2_O followed by H-additions and that the used DFT methods are actually accurate enough for this purpose, as shown above. In this case, the error in the binding energies self-canceled when deriving the energy features (energy barriers and reaction energies) of the reactions.

### Reactivity between CCH and H_2_O on the Ice Surface Models

#### W18 ASW Ice Cluster Model

Following the three reaction types found in the benchmark study, we tried to reproduce them on top of the W18 ice cluster model. However, significant differences in the mechanisms have been found, precisely because they occur on the ice model. Indeed, the larger number of water molecules indicates that (i) there are no concerted mechanisms at work and (ii) water-assisted proton-transfer reactions are operative. Water-assisted proton-transfer mechanisms were elucidated theoretically in the latter years of the last century^109^ and can induce important decrease in the energy barriers with respect to the nonwater-assisted analogues. This is because the assisting water molecules bridge the accepting/donating proton processes with their neighboring molecules and, at the same time, reduce the strain of the rings in the transition-state structures. Hence, this mechanism is also called proton-transport catalysis. Such catalytic effects have also been observed in processes of interstellar interest in icy surfaces.^58,110,111^ It is worth mentioning that the water-assisted proton transfers, to be catalytically effective, need a proper H-bond connectivity among the bridging water molecules, from the first to the last proton transfer. This means that, if the H-bonding network is truncated because of the presence of interstitial unpurities, the mechanism is not operative. This is an aspect not to overlook in interstellar ices because they contain minor volatile species in the ices that can obstruct the chained proton relays. Remarkably, it is worth stressing that the catalytic transfers involve H atoms with a proton character and not atomic radicals. This is because the H species exchanged during the transfers are proton-like atoms chemically bonded to a more electronegative O atom.

Because the identified reaction channels on W18 differ significantly from the gas-phase ones, we redefine the **R′1-3** model channels into **R1-3**, which adopt the following simplified mechanistic steps:

R1 H_2_O + CCH → OH + HCCH → HCCHOH
R2 H_2_O + CCH → CC(H)–OH_2_ → HCCHOH
R3 H_2_O + CCH → H_2_O–CCH → H_2_CCOH

The stationary points and the energy profiles of these reaction pathways are shown in Figure 3.

The **R1** path remains the same as the **R′1** one, namely, formation of HCCH and OH as intermediate species by H-transfer from the reactive H_2_O molecule to CCH, and then coupling of the OH to HCCH to form the HCCHOH radical as vinyl alcohol precursor. Both **R2** and **R3** start with the formation of the hemibonded systems (see the Supporting Information for their spin densities). However, because the involved C atoms are different, the pathways proceed in a different way as well. The hemibonded structure of **R2** evolves toward the formation of the CC(H)–OH_2_ intermediate, which is followed by a proton transfer, from the OH_2_ moiety and adopting a water-assisted proton-transfer mechanism, to form the final HCCHOH radical. In contrast, the hemibonded structure of **R3** transforms into H_2_O–CCH as the intermediate species. From this intermediate, two different paths are possible, namely, from the OH_2_ moiety, a proton transfer to (i) the central C atom (TS2-1) or (ii) the terminal C atom (TS2-2), forming HCCHOH or H_2_CCOH, respectively. Interestingly, both paths proceed through a water-assisted proton-transfer mechanism. The fact that **R3** exhibits these two paths is because there are well-oriented H-bonds in the water cluster allowing for the water-assisted mechanism in two directions, while this is not possible in **R2** because of the geometry of the intermediate structure.

In relation to the energetics (see also Figure 3), the first steps (TS1) of paths **R1** and **R2** become submerged below the energy of reactants once they are ZPE-corrected. The same happens for the second step (TS2) of path **R2**. This means that **R2** is an overall effectively barrierless reaction on W18. In contrast, **R1** has a low-energy intermediate (associated with the formation of HCCH), and consequently, the second step (TS2) encounters a relatively high energy barrier of 34.6 kJ mol^–1^ (with respect to the intermediate). For **R3**, the first step already presents a non-negligible energy barrier, even when it is ZPE-corrected (14.1 kJ mol^–1^). For the second steps, TS2-1 has an energy lower than that of TS2-2 because the geometry associated with the latter saddle point is more strained, because the cycle created by the water assistant molecules is smaller. All the reaction paths present negative and very large reaction energies, indicating that the formed vinyl alcohol radicals are more stable than the reactant states. We observed that, in some cases, the ZPE corrections added to the potential energies are very large (see, for instance, TS1 in **R1**, TS2 in **R2**, and TS2-1 and TS2-2 in **R3**). Interestingly, these transition states involve the motion of H atoms of the water molecules, either as a direct proton transfer (case of TS1 in **R1**) or as a water-assisted proton-transfer mechanism (cases of TS2 in **R2** and TS2-1 and TS2-2 in **R3**). In contrast, when a C–O bond is formed (namely, without involving any proton motion) it results in a slight ZPE correction. In order to understand this behavior, we simulated the IR spectra of R, TS1, I, and TS2-1 of **R3** (see the Supporting Information). The spectra of R and TS1, where only the C–O bond forms, show mostly common features, with the exception of a couple of bands around 3000 cm^–1^, because of O–H stretching of the water molecules surrounding the CCH. On the other hand, comparing the spectrum of TS2-1 with R highlights the presence of a number of features at a shorter wavenumber, arising from the water-assisted proton-transfer. Given that the ZPE arises from the sum of the energy of all the vibrational modes, the shorter wavelength features of TS2-1 explain why its ZPE is smaller than R and hence why the resulting ΔZPE is negative.

As a final comment, we stress the influence of the saturated state (in terms of H-bonding) of the reacting water molecule in the energy barriers. Indeed, according to the potential energy values, the TS1 barrier in **R2** is actually lower than that in **R3**, 0.7 and 16.0 kJ mol^–1^, respectively (see Figure 3). This is because, in the former, TS1 is more reactant-like than in the latter because, in the R structure of **R2**, the reacting water molecule is not fully saturated by H-bonds (*i.e.*, 2 H-bonds as donor +1 H-bond as acceptor) while this is the case in **R3** (*i.e.*, 2 H-bonds as donor +2 H-bonds as acceptor). The lack of the H-bond in R of the **R2** reaction allows the water “unsaturated” lone pair to form the hemibonded system with CCH, in which the C–O bond is half-formed. Thus, to reach the TS1 structure, no significant energy requirements and geometrical changes are needed, and hence the low energy barrier. In contrast, in **R3**, to form TS1 from the R structure, the reacting water molecule has to break one of the H-bonds to form the new C–O bond, implying a more energetic cost and relevant geometrical changes. Nevertheless, the associated quantum tunnelling crossover temperature is rather high (244 K), indicating that this channel could be relevant at interstellar temperatures.

#### W33 ASW Ice Cluster Model

After modeling the reaction on the flat surface of W18, we set out to investigate the possible effects of a cavity structure like that of W33. We tried to reproduce the same three reaction paths as those taking place on W18, but we found only two mechanisms, and both are similar to **R2**; hence, they are termed **R2-1** and **R2-2**. The stationary points and the energy profiles of these reaction pathways are shown in Figure 4. It is worth mentioning that, to model the reaction path **R2-2**, it was necessary to fix the position of some of the oxygen atoms placed at the edge of the model because full geometry relaxation led to the collapse of the cavity.

In both cases, CCH is located in the cavity, with the central C atom interacting with the oxygen atom of the reactant water molecule of the surface, forming the aforementioned hemibonded systems (see the Supporting Information for spin densities). Along the reactions, these structures evolve to form the CC(H)–OH_2_ intermediate. For both cases, this step has a very low ZPE-corrected energy barrier (2.1 and 0.8 kJ mol^–1^ for TS1 and TS2, respectively). The second step involves, for both paths, the proton transfer from the OH_2_ moiety to the terminal C atom forming the HCCHOH radical, also by means of a water-assisted proton-transfer mechanism. The two paths lead to the same radical because the H-bond network enables the water-assisted proton-transfer to connect with the terminal C atom and not the central one. These second steps are energetically below the corresponding intermediates (by including ZPE corrections), and accordingly, the water-assisted proton transfers proceed in a barrierless fashion. Because the reaction energies are very large and negative, the computed reactions on W33 are energetically very favorable, similarly to the **R2** reaction occurring on W18.

### Toward the Formation of Ethanol: Hydrogenation of Vinyl Alcohol

As shown above, reaction of CCH with an icy water molecule leads to the formation of CHCHOH and H_2_CCOH. From these species, to reach ethanol, a set of hydrogenation steps are necessary, as sketched in reactions 6–8.
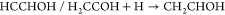
6
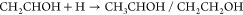
7
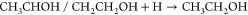
8The first hydrogenation forms vinyl alcohol (VA). As this H-addition is a radical–radical coupling, we consider it as barrierless. The second hydrogenation step is the H-addition to the VA. It involves the reactivity between a closed-shell species (namely, VA) with a radical (H), and accordingly, it is expected to have an activation barrier. Interestingly, depending on the C in which the H-addition takes place, the species formed is either CH_3_CHOH or CH_2_CH_2_OH. The third and final hydrogenation step leads to the formation of ethanol, irrespective of the initial radical species. This H-addition is, again, a radical–radical coupling, and accordingly, it is expected to be barrierless in a similar fashion as the first hydrogenation. According to this reactive scheme, to investigate on ethanol formation, we simulated only reaction 7 on the W18 ice model.

**Figure 5:**
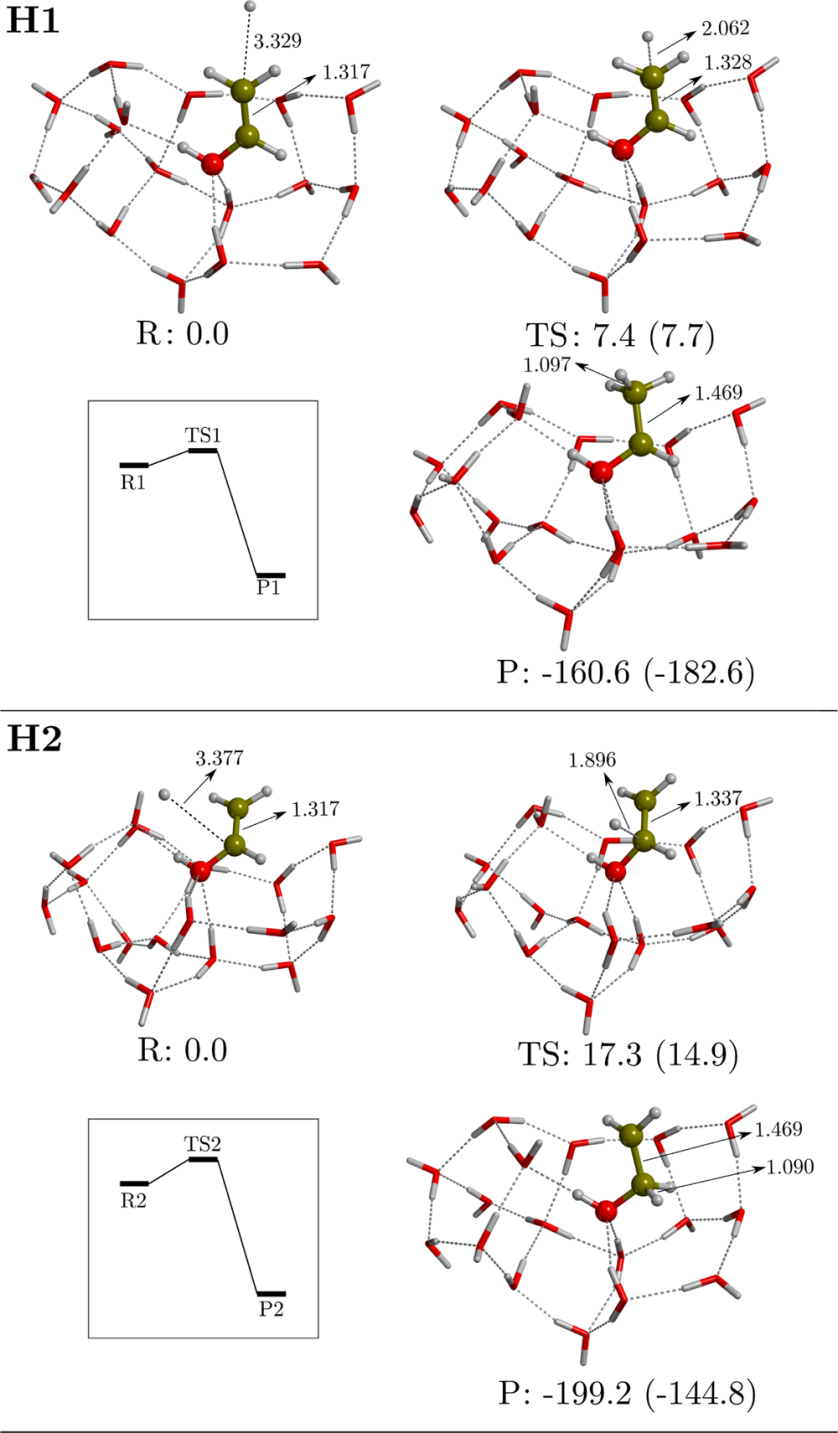
Computed potential energy surfaces (PESs) of the hydrogenation reactions **H1** and **H2** on W18 ASW ice cluster model. Bare energy values correspond to relative ZPE-corrected values, while values in parentheses correspond to those missing this correction. The miniature panels sketch the ZPE-corrected PESs. Energies are in kJ mol^–1^, and distances are in Å.

We identified two reaction paths, termed **H1** and **H2** (see Figure 5), the difference of which being the C atom that undergoes the H-addition, in analogy to the gas-phase **H′1** and **H′2** reactions (see Figure 2). Both reactions start from a prereactant structure in which the H atom is *ca.* 3.3 Å from the VA because of the weak interactions between the two partners. The computed potential energy surfaces indicate that the two hydrogenation reactions present a non-negligible energy barrier. In agreement with gas-phase results, the path leading to the formation of CH_3_CHOH is more favorable than that resulting with CH_2_CH_2_OH, in terms of both energy barriers and reaction energies. Remarkably, the computed energy barriers on W18 are higher (3–4 kJ mol^–1^) than those obtained in the gas-phase. This is probably because the adsorption of VA with the surface induces an enhanced stabilization of the former due to the intermolecular forces between VA and the icy surface. Thus, chemically strictly speaking, the surface does not act as a chemical catalyst but slightly inhibits the process. However, it is worth highlighting that the **H1** reaction presents a very low potential energy barrier and, because of the involvement of a H atom and the very low temperatures of the ISM, tunneling effects can operate as well. Indeed, its associated quantum tunnelling crossover temperature is 118 K. For reaction **H2**, the crossover temperature is slightly higher, 174 K; therefore, quantum tunneling could also play a role.

### Isomerization between Vinyl Alcohol and Acetaldehyde

Vinyl alcohol and acetaldehyde are tautomers (*i.e.*, structural isomers), and therefore, we have here considered the conversion from one to the other according to reaction 9. This isomerization reaction has been computed at the MPWB1K-D3(BJ)//6-311++G(2df,2pd)//MPWB1K-D3(BJ)//6-311++G(d,p) level for consistency with the methodology applied to the hydrogenation of vinyl alcohol.
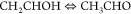
9Among them, acetaldehyde is the most stable isomer (by ∼41 kJ mol^–1^). In the gas phase, the barrier connecting vinyl alcohol with acetaldehyde is very high (237.9 kJ mol^–1^, see the Supporting Information). This is because the mechanism involves an intramolecular H-transfer from the OH of vinyl alcohol to the terminal C atom, with a highly strained transition state.

**Figure 6:**
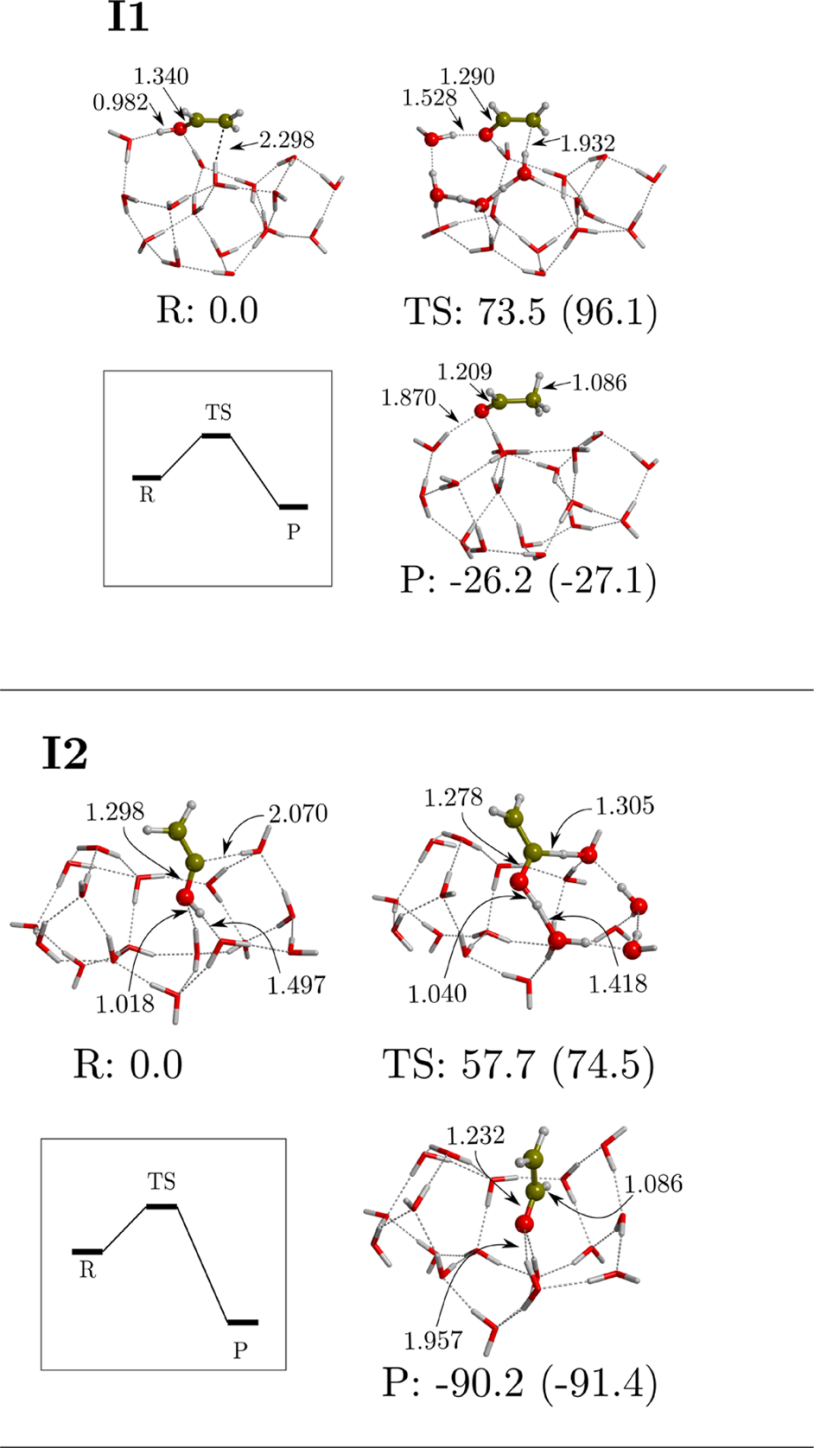
Computed potential energy surfaces (PESs) of the isomerization between vinyl alcohol and acetaldehyde (**I1**) and the analogue reaction involving their precursor, *i.e.*, the product P2 of R3 (**I2**) on W18 ice cluster model at MPWB1K-D3(BJ)/6-311++G(2df,2pd)//MPWB1K-D3(BJ)/6-311++G(d,p). Bare energy values correspond to relative ZPE-corrected values, while values in parentheses correspond to those missing this correction. The miniature panel sketches the ZPE-corrected PESs. Energies are in kJ mol^–1^, and distances are in Å.

If we now consider this reaction to take place through the water molecules of W18, as shown in Figure 6, we can see that a water-assisted proton-transfer mechanism can take place, lowering the activation energy barrier down to 73.5 kJ mol^–1^ when the reaction starts from vinyl alcohol (**I1**) and to 57.7 kJ mol^–1^ when it starts from its less hydrogenated precursor (**I2**). The latter reaction produces CH_2_CHO, which can be successively hydrogenated to form acetaldehyde. The difference in the two activation barriers is probably due to the structure of the ring through which the proton is transferred; that is, in **I1** it is more strained. This is a great example of how interstellar ices act as chemical catalysts. However, computed energy barriers are very high to be surmountable under interstellar conditions, and accordingly, these channels seem unlikely. Nevertheless, we note that the barrier of **I2** is narrower and lower than that of **I1** (with barriers widths of about 1.6 and 2.2 Å, respectively, assuming the asymmetric Eckart barrier model; see ref (112)). Therefore, **I2** would be the most efficient mechanism among the two, if assuming that quantum tunneling does play a role.

## Discussion and Astrophysical Implications

CCH is a highly reactive species that easily reacts with water. Because it is a C-centered radical and contains both donor and acceptor H-bond atoms (the H and the C-end atoms, respectively), it tends to form H-bonded and hemibonded complexes with water ice molecules. The most relevant computed energetics of CCH reactivity on our ASW ice models are summarized in Table 3. On W18, **R3** presents an activation barrier of 14.4 kJ mol^–1^, and accordingly, it is not *a priori* an efficient path to form vinyl alcohol, although it could still be relevant if quantum tunnelling effects work. The first step of **R1** is barrierless to form HCCH + OH, but the reaction stops at this stage. This is because the second step has an intrinsic energy barrier of 34.6 kJ mol^–1^ (the energy difference between TS2 and I; see Figure 3). Moreover, the energy released by the first reaction step will no longer be available for the second step, as water ices tend to efficiently dissipate chemical energy fast.^113,114^ Therefore, **R1** will lead to the formation of a highly stabilized acetylene, which will hardly react with OH remaining stuck on the ice. However, this reaction can be an effective channel toward the formation of OH radicals on the ice surfaces without the need of a direct energy processing. This can be of importance because the generated OH could participate in further surface reactions in the form of OH additions. **R2**, at variance with the other two reactions, is an effective barrierless reaction path toward vinyl alcohol formation. Because of that, this reaction has also been modeled on W33 to study the effects of the cavity on the reactivity. On W33, we found that the reactions are no more barrierless, although the two identified paths present low activation barriers (2.1 and 0.8 kJ mol^–1^ for **R2-1** and **R2-2**, respectively). Because these two pathways are equivalent to **R2** on W18, the effect of the cavity is almost insignificant for the energetics of the path. Therefore, we can consider that the mechanistic steps represented by the **R2**, **R2-1**, and **R2-2** pathways constitute the most likely channel to form vinyl alcohol.

**Table 3:** Summary of the Energetics of the Simulated Reactions and Their Products

reaction		barrier	product
W18	R1	NO	HCCH + OH
	R2	NO	HCCHOH
	R3	14.4 kJ mol^–1^	H_2_CCOH/HCCHOH
W33	R2-1	2.1 kJ mol^–1^	HCCHOH
	R2-2	0.8 kJ mol^–1^	HCCHOH
W18	H1	7.4 kJ mol^–1^	CH_3_CH_2_OH
	H2	17.3 kJ mol^–1^	CH_3_CH_2_OH

The hydrogenation of vinyl alcohol on grains leads to the formation of ethanol, as occurring in methanol formation from CO^115−119^ and ethane formation from C_2_H_2_ and C_2_H_4_.^120−122^ The H-addition to vinyl alcohol is the only hydrogenation step not involving a barrierless radical–radical coupling and presents an energy barrier of 7.4 and 17.3 kJ mol^–1^ for the **H1** and **H2** channel (see Table 3). Thus, **H1** is the most energetically favored pathway for the formation of the ethanol radical precursor. Despite the computed relatively high energy barriers considering very low temperatures, these reactions can proceed through tunneling and are accordingly operative in the reaction chain starting from the adsorption of CCH on water ice.

These quantum chemical results can partly be related to the experimental findings mentioned in the Introduction. Indeed, part of the experimental synthetic routes have been simulated here, providing an atomistic interpretation (including the energetics) for the formation of vinyl alcohol followed by it hydrogenation to form ethanol. The main difference between the experiments and our computations is that, in the experiments, the C_2_H_2_/H_2_O ices need to be processed to trigger reactivity (probably due to generating the radical reactive species like CCH and OH), while in our simulations the assumption is that the reaction does not require energetic processing of ices, because the CCH is readily available in the gas phase in a wide variety of environments.

We also considered the isomerization of vinyl alcohol into acetaldehyde (and the same for their less hydrogenated precursors), because in the experiments these reactions were suggested to explain the presence of both vinyl alcohol and acetaldehyde. According to our calculations, however, these reactions, although being catalyzed by the surfaces thanks to a water-assisted proton-transfer mechanism, present high energy barriers, rendering them poorly competitive to the final H additions. However, experimental authors pointed out that the isomerization could take place thanks to the exothermicity of the previous steps, in which the energy released along the reaction steps can be used to overcome the isomerization energy barrier. Our computed energetic data is consistent with this view. The very favorable reaction energies shown by the reactions indicate that the energy barriers of the isomerization processes lay below the prereactive asymptotic states, and therefore, they can be overcome by making use of the nascent reaction energies. However, one should bear in mind that water ice surfaces are extraordinary third bodies^113,114^ and accordingly, the direct transfer of the previous reaction energies to surmount the isomerization energy barriers is doubtful. To shed some light on this topic, dedicated *ab initio* molecular dynamics simulations are needed, which is outside scope of the present work.

Finally, results presented in this work are very relevant in the framework of cold astrochemistry. The presence of abundant (∼10^–9^–10^–8^) gaseous CCH radicals in cold (≤20 K) regions, where water ices envelope the refractory cores of the interstellar dust grains (see Introduction), can lead to the formation of vinyl alcohol and ethanol in addition to HCCH and OH through a competitive reaction channel. Remarkably, at variance with most of the experimentally proposed mechanisms (see Introduction), the formation of the aforementioned products does not require the energetic processing of interstellar ices. Regarding the formation of iCOMs, our proposal complements the nonenergetic reaction scheme of Chuang *et al.*,^123^ in which OH radicals attack C_2_H_2_ to form iCOMs. We note that CCH (and the other intermediate radicals) could also be destroyed by other competitive surface reactions, *e.g.*, by H-abstraction reactions with H_2_, or H-additions. This will be considered in the future.

In general, the astrochemical processes in cold regions such as the ones described in this work are important to improve our understanding of the presence of complex species detected in the cold (<20 K) outskirts of prestellar cores during the past decade (*e.g.*^16,124−128^). In this vein, ethanol has recently gained some attention, as it has been advocated to be the precursor of several iCOMs formed by cold gas-phase reactions (the genealogical tree of ethanol^23,91^). Indeed, the correlation of glycoladehyde and acetaldehyde abundances observed toward a number of interstellar sources has been shown to follow very well the theoretical predictions when their synthesis takes place through this gas-phase scheme.^24^ In the present work, we showed that an efficient path exists for the formation of ethanol on the surfaces of interstellar ices. However, the nonthermal desorption of ethanol (and of the other products) remains as a crucial missing step in the sequential events linking the chemistry of iCOMs occurring on the surface of grains and in the gas phase in cold regions; this issue is undoubtedly a central matter of further investigation.

## Conclusions

Interstellar complex organic molecules (iCOMs) have been detected in different astrophysical environments. However, the chemistry leading to their formation is not unambiguously known. Two prevailing paradigms have been largely used to rationalize their presence in the interstellar medium: one advocates reaction in the gas-phase, whereas the other presents reaction on the surface of icy grains. In this work, we focused on the latter by computing the reaction of CCH with a H_2_O molecule forming part of the ice structure, leading to vinyl alcohol (CH_2_CHOH), which upon hydrogenation is converted into ethanol (CH_3_CH_2_OH). This reaction is proposed as an alternative synthetic route for iCOMs beyond the commonly assumed radical–radical couplings. Investigations have been performed by means of DFT quantum chemical simulations and adopting cluster models of 18 and 33 water molecules (W18 and W33) to mimic the icy surfaces. For the reaction of CCH with H_2_O, three different reaction pathways have been elucidated, leading to the formation of HCCH + OH, CHCHOH, and H_2_CCOH. Some cases present small activation barriers, but in others the reactions are barrierless when zero-point energy corrections are accounted for. Hydrogenation of vinyl alcohol on the W18 cluster has been found to present activation barriers of a certain significance but, for these reactions, quantum tunneling is likely to be at work, speeding them up. Isomerization between vinyl alcohol and acetaldehyde have also been simulated on W18, with the results indicating that, despite the strong catalytic role played by the water ice, they have a barrier of significant height. Additionally, the direct H-abstraction from the water molecule to CCH leading to the formation of HCCH and the OH radical on the surface has been found to be an energetically competitive channel. It is worth mentioning that a chemical kinetics treatment of these results is underway in order to compute the rate constants (including tunneling effects explicitly) for each of the proposed reactive channels and hence elucidate branching ratios and formation efficiencies of the simulated paths.

In summary, results from our calculations indicate that the reaction of CCH with water ice can lead to the formation of vinyl alcohol and, lately, to the production of ethanol, and likely acetaldehyde, without the need of ice energy processing. This conclusion is of relevance in the context of iCOM formation because, according to the genealogical tree of ethanol, this species is the parent molecule through which different iCOMs (*e.g.*, formic acid, formaldehyde, glycolaldehyde, and others) can form by means of gas-phase processes.^23,24^ Thus, in this work, we provide quantum chemical evidence on the feasibility of our mechanistic proposal, in which ethanol can be formed on interstellar icy grain surfaces, hence linking the two paradigms in the synthesis of iCOMs, at least in this particular case. The missing link between on-grain and gas-phase chemistry stands in the nonthermal desorption of ethanol and its precursors, which should be a subject for further investigation.

## Supporting Information Available

Structures and errors of the benchmarking study; energetics relative to the CCH + H_2_O reactions on W18 and W33; contributions to the binding energies of CCH on W18 and W33; Mulliken spin densities and spin density maps of the complexes CCH + H_2_O in the gas phase and on W18 and W33; energetics relative to the hydrogenation of vinyl alcohol; energetics relative to the isomerization of vinyl alcohol into acetaldehyde (PDF)
