# Supplementary material for: Non-energetic Formation of Ethanol via CCH Reaction with Interstellar H2O Ices. A Computational Chemistry Study
Source: ACS Earth Space Chem. 2022 Mar 7;6(3):496–511. doi: 10.1021/acsearthspacechem.1c00369 (PMC8935465; doi:10.1021/acsearthspacechem.1c00369)
Supplement: Supplementary file 1 — sp1c00369_si_001.pdf [file sp1c00369_si_001.pdf]

Non-Energetic Formation of Ethanol via CCH Reaction with Interstellar H_2O ices. A Computational Chemistry Study

Jessica Perrero,[*,†,‡] Joan Enrique-Romero,[¶,†] Berta Martínez-Bachs,[†] Cecilia Ceccarelli,[¶] Nadia Balucani,[§,¶,‖] Piero Ugliengo,[‡] and Albert Rimola[*,†]

†*Departament de Química, Universitat Autònoma de Barcelona, Bellaterra, 08193, Catalonia, Spain*

‡*Dipartimento di Chimica and Nanostructured Interfaces and Surfaces (NIS) Centre, Università degli Studi di Torino, via P. Giuria 7, 10125, Torino, Italy.*

¶*Univ. Grenoble Alpes, CNRS, Institut de Planétologie et d'Astrophysique de Grenoble (IPAG), 38000 Grenoble, France*

§*Dipartimento di Chimica, Biologia e Biotecnologie, Università di Perugia, Via Elce di Sotto 8, 06123 Perugia, Italy*

‖*Osservatorio Astrosico di Arcetri, Largo E. Fermi 5, 50125 Firenze, Italy*

E-mail: juan.enrique-romero@univ-grenoble-alpes.fr; albert.rimola@uab.cat

Data of the benchmarking study

Table S1: Mulliken spin densities at ωB97X-D3/6-311++G(2df,2pd) of the reactants in the gas phase reactions **R'1** and **R'2**. **R'3** has the same reactant structure as **R'1**. Atoms of CCH molecule are labelled C2-C1-H, while atoms from H_2O are H_a-O-H_b.

	C2	C1	H	**Sum CCH**	O	H_a	H_b	**Sum H_2O**	Total
**R'1**	0.990454	-0.101992	0.005722	**0.894184**	0.108729	-0.001457	-0.001456	**0.105816**	1.000000
**R'2**	0.552337	0.393607	-0.011362	**0.934582**	0.062076	0.001671	0.001671	**0.065418**	1.000000

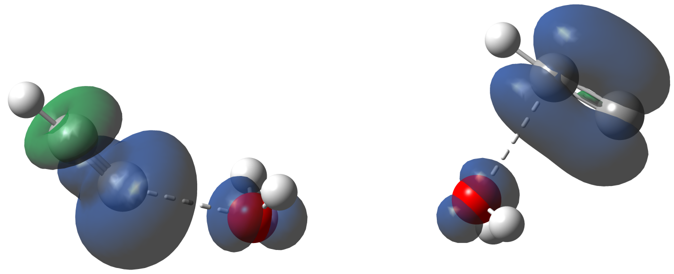

Figure S1: Spin density maps of the CCH + H_2O dimers for the reactant structures in **R'1** (left) and **R'2** (right).

Table S2: Raw energetic data of the benchmarking study. Absolute potential energy values calculated at DFT were obtained by running DFT-D/6-311++G(2df,2pd)//DFT-D/6-311++G(d,p) optimizations + single points, see main text for more details. Reference values were obtained at CCSD(T)/aug-cc-PVTZ//BHLYP-D3(BJ)/6-311++G(d,p), except for the TS1 structure of **R'2**, marked with an asterisk, which was obtained at CCSD(T)/aug-cc-PVTZ//ωB97X-D3/6-311++G(d,p). Energy units in Hartree.

**R'1**	BHLYP-D3(BJ)	M062X-D3	MPWB1K-D3(BJ)	PW6B95-D3(BJ)	ωB97X-D3	CCSD(T)
H2O + CCH	-153.013156457	-153.026600588	-153.013696426	-153.281805058	-153.055341584	-152.81644205
TS1	-153.002305407	-153.023223410	-153.003927795	-153.274340369	-153.044307971	-152.80536306
OH + HCCH	-153.048050346	-153.048294518	-153.041293625	-153.305785084	-153.079088878	-152.84271842
TS2	-153.039249817	-153.050528257	-153.036622503	-153.305091890	-153.075714706	-152.83603921
HCCHOH	-153.095817220	-153.111172069	-153.096610660	-153.359358356	-153.133782225	-152.89027409
**R'2**	BHLYP-D3(BJ)	M062X-D3	MPWB1K-D3(BJ)	PW6B95-D3(BJ)	ωB97X-D3	CCSD(T)
H2O + CCH	-153.009733360	-153.025837932	-153.005624340	-153.273611902	-153.049137706	-152.80613195
TS1*	-152.965026052	-152.984891814	-152.968902477	-153.239018589	-153.008017387	-152.76824032
HOCCHH	-153.104694442	-153.119841938	-153.107044694	-153.370068228	-153.143511897	-152.89798091
**R'3**	BHLYP-D3(BJ)	M062X-D3	MPWB1K-D3(BJ)	PW6B95-D3(BJ)	ωB97X-D3	CCSD(T)
H2O + CCH	-153.013156457	-153.026600588	-153.013696426	-153.281805058	-153.055341584	-152.81644205
TS	-152.966295093	-152.993180990	-152.976150890	-153.234783660	-153.012305240	-152.77629188
HOCCHH	-153.104694442	-153.119841940	-153.105834483	-153.368966561	-153.142813429	-152.89798091

Table S3: Unsigned error for each DFT method tested in the benchmarking study with respect to CCSD(T). Values are in percentage.

**R'1**	BHLYP-D3(BJ)	M062X-D3	MPWB1K-D3(BJ)	PW6B95-D3(BJ)	ωB97X-D3
H2O + CCH	-	-	-	-	-
TS1	2.1	69.5	11.8	32.6	0.4
OH + HCCH	32.8	17.4	5.0	8.7	9.6
TS2	33.1	22.1	17.0	18.8	4.0
HCCHOH	12.0	14.5	12.3	5.0	6.2
AVERAGE	20.0	30.9	11.5	16.3	5.1
**R'2**	BHLYP-D3(BJ)	M062X-D3	MPWB1K-D3(BJ)	PW6B95-D3(BJ)	ωB97X-D3
H2O + CCH	-	-	-	-	-
TS1	18.0	8.1	3.1	8.7	8.5
HOCCHH	3.4	2.3	10.4	5.0	2.7
AVERAGE	10.7	5.2	6.8	6.9	5.6
**R'3**	BHLYP-D3(BJ)	M062X-D3	MPWB1K-D3(BJ)	PW6B95-D3(BJ)	ωB97X-D3
H2O + CCH	-	-	-	-	-
TS	16.7	16.8	6.5	17.1	7.2
HOCCHH	12.3	14.4	13.0	6.9	7.3
AVERAGE	14.5	15.6	9.7	12.0	7.2

Binding energies of CCH on W18 and W33

Table S4: Binding energies (BE, in kJ mol^{-1}) of the $CCH \cdots H_2O$ dimer complexes adopting H-bonded and hemibonded structures (see S2) computed at the different DFT methods and compared with those obtained at CCSD(T) level. Distances (in Å) of the H-bond and the C-O are also shown. DFT values were computed by performing single point energy calculations at DFT/6-311++G(2df,2pd) level on the optimized geometries at DFT/6-311++G(d,p) and benchmarked against single point energies computed at CCSD(T)/aug-cc-VTZP on the wB97x-D3/6-311++G(d,p) optimized geometries.

Method	BE		Distances	
	H-bonded	Hemibonded	H-bonded	Hemibonded
CCSD(T)	18.3	10.7	2.111	2.324
ωB97X-D3	15.3	16.9	2.111	2.325
BHLYP-D3(BJ)	16.4	13.7	2.083	2.389
M062X-D3	15.2	16.5	2.096	2.550
MPWB1K-D3(BJ)	14.8	16.5	2.111	2.324
PW6B95-D3(BJ)	15.0	23.2	2.122	2.303
B3LYP-D3(BJ)	15.7	25.9	2.085	2.297

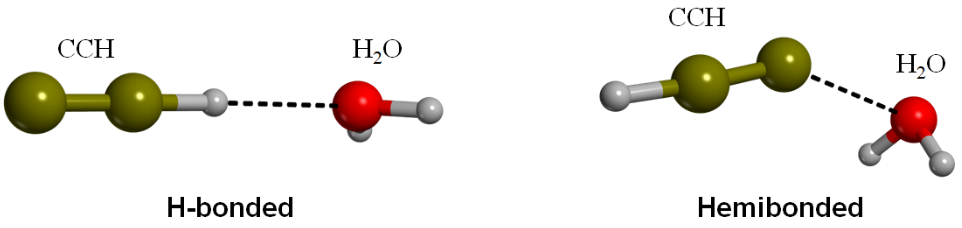

Figure S2: H-bonded and hemibonded complex between CCH and one water molecule used in the benchmarking study for BE calculation.

Table S5: Mulliken spin densities at ωB97X-D3/6-311++G(2df,2pd) of the reactants for the reactions **R1**, **R2** and **R3** occurring on the W18 and W33 clusters. Atoms of CCH molecule are labelled C2-C1-H, while atoms of the reacting water molecule H_2O are H_a-O-H_b. In reaction **R1** there is no hemibond, in **R2** the atom C1 is interacting with oxygen to form a hemibond, while in **R3** C2 forms the hemibond with water.

**W18**	C1	C2	H	**Sum CCH**	O	H_a	H_b	**Sum H_2O**	Total
R1	0.503069	0.478872	0.024583	**1.006524**	-0.003686	-0.000571	0.001811	**-0.002446**	1.004078
R2	0.413329	0.473437	-0.009109	**0.877657**	0.104090	0.004954	0.007772	**0.116816**	0.994473
R3	-0.171422	0.998879	0.006988	**0.834445**	0.125833	0.009058	0.016855	**0.151746**	0.986191
**W33**	C1	C2	H	**Sum CCH**	O	H_a	H_b	**Sum H_2O**	Total
R2-1	0.316328	0.557183	-0.009007	**0.864504**	0.099037	0.002760	0.005175	**0.106972**	0.971476
R2-2	0.343767	0.513275	-0.007151	**0.849891**	0.131947	-0.010140	0.007339	**0.129146**	0.979037

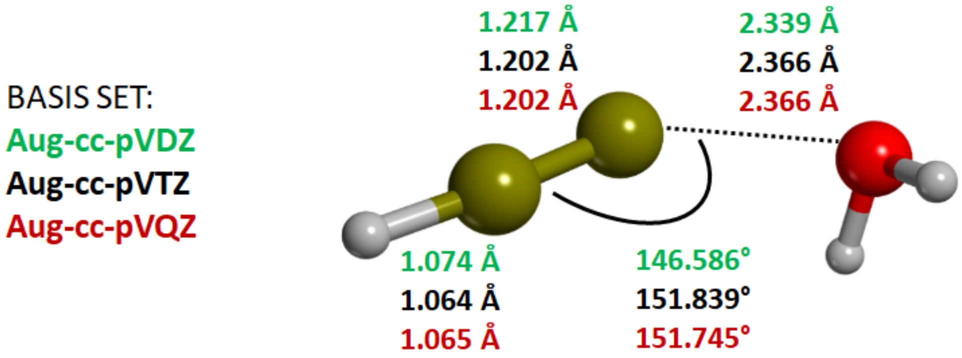

Figure S3: Optimized geometries of CCH + H_2O complex at wB97X-D3/aug-cc-pVnZ (with n = D,T,Q) level of theory. Small structural variations occur between DZ and TZ quality, while geometrical parameters obtained at TZ and QZ quality are almost identical.

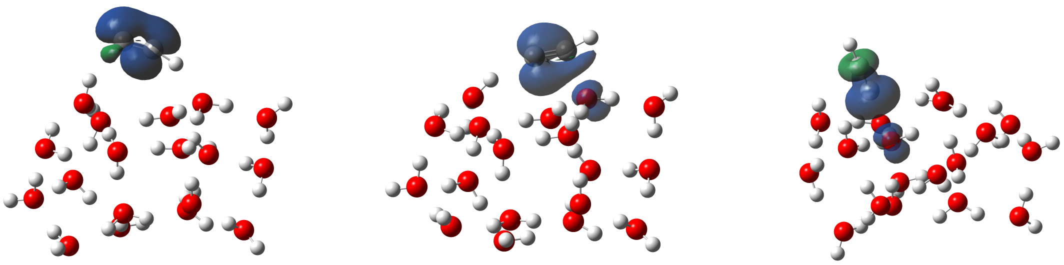

Figure S4: Spin density maps for the three reactant geometries on top of the W18 ice model. From left to right, **R1**, **R2** and **R3**, respectively.

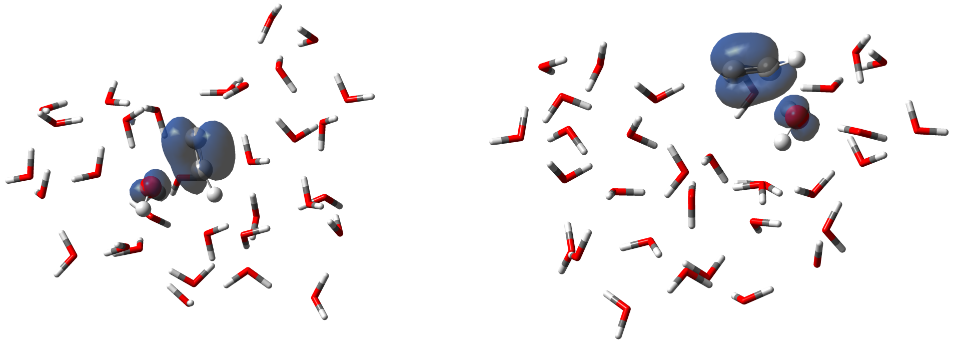

Figure S5: Spin density maps for the three reactant geometries on top of the W33 ice model, for **R2-1** (left) and **R2-2** (right)

Table S6: Absolute energy values of isolated CCH, W18 and W33 ice cluster for the calculation of the BEs. Calculations are done at ωB97X-D3/6-311++G(2df,2pd) level of theory (tζ-ext), with the exception of the ZPE corrections, which are obtained at ωB97X-D3/6-311++G(d,p) level of theory (tζ)

	tζ-ext		tζ
	U+D	D	ZPE
W18	-1376.259998	-0.0384204659	0.467565
W33	-2523.167133	-0.0779360911	0.844601
CCH	-76.60504885	-0.0000916354	0.015182

Table S7: Absolute energy values of CCH adsorbed on W18 and on W33 for the calculation of the BEs. Calculations are done at ωB97X-D3/6-311++G(2df,2pd) level of theory (tζ-ext), with the exception of the ZPE corrections, which are obtained at ωB97X-D3/6-311++G(d,p) level of theory (tζ).

	tζ-ext					tζ
**W18**	U+D	D	BSSE	A(B) ice	(A)B cch	ZPE
R1	-1452.874388110	-0.039770328	0.00051692671	-1376.251064	-76.59995526	0.482535000
R2	-1,452.881405880	-0.042376974	0.000782677333	-1376.247627	-76.599804	0.483883000
R3	-1,452.885737960	-0.046945017	0.000819352204	-1376.256773	-76.6048896	0.483598000
**W33**	U+D	D	BSSE	A(B) ice	(A)B cch	ZPE
R2-1	-2599.811892	-0.0807698358	0.001274683073	-2523.169147	-76.59983078	0.864056000
R2-2	-2599.810529	-0.0852829482	0.001331610724	-2523.15878	-76.59989697	0.864055000

Table S8: Contributions to the binding energy of CCH on W18 and W33. Calculations are done at ωB97X-D3/6-311++G(2df,2pd) level of theory, with the exception of the ZPE corrections, which are obtained at ωB97X-D3/6-311++G(d,p) level of theory. U is the potential energy contribution, D the dispersion contibution, BSSE the basis set superposition error values, δice the deformation of the ice model and δcch the deformation of CCH. The BE is the negative value of the the sum of U + D + BSSE + ZPE.

**W18**	U	D	ZPE	BSSE	δice	δcch	BE
R1	-21.2	-3.3	-0.6	1.4	23.5	13.4	23.7
R2	-32.8	-10.1	3.0	2.1	32.5	13.8	37.9
R3	-32.2	-22.1	2.2	2.2	8.5	0.4	49.9
**W33**	U	D	ZPE	BSSE	δice	δcch	BE
R2-1	-97.1	-7.2	11.2	3.3	-5.3	13.7	89.7
R2-2	-81.6	-19.0	11.2	3.5	21.9	13.5	86.0

Energetics of CCH reactivity on W18 and W33 ASW ice models

Table S9: ωB97X-D3-absolute energy values relative to the potential energy surfaces for the reactivity of CCH on W18. tζ means 6-311++G(d,p), while tζ-ext means 6-311++G(2df,2pd); *opt* and *freq* indicate optimisation and frequency calculation (needed for ZPE calculation) and *sp* means single point energy calculation. U+D refers to the potential energy (U) plus the dispersion correction (D), and ZPE is the zero point energy correction. Energy units are in Hartree.

W18 ωB97X-D3	tζ (*opt* and *freq*)				tζ-ext (*sp*)
**R1**	U+D	D	ZPE	iν (cm-1)	U+D
H_2O + CCH	-1452.81211576	-0.03977033	0.48253500	-	-1452.87438811
TS1	-1452.80761020	-0.04443524	0.47784100	-1385.8	-1452.87075047
OH + HCCH	-1452.85014377	-0.04792195	0.48315500	-	-1452.91275921
TS2	-1452.83595685	-0.04100965	0.48175600	-40.4	-1452.89816469
HCCHOH	-1452.89202514	-0.04368950	0.48666500	-	-1452.95574465
**R2**	U+D	D	ZPE	iν (cm-1)	U+D
H_2O + CCH	-1452.81937532	-0.042376974	0.48388300	-	-1452.88140588
TS1	-1452.81862996	-0.042681317	0.48361200	-165.9	-1452.88113464
CC(H)–OH_2	-1452.82239800	-0.043392271	0.48466800	-	-1452.88617717
TS2	-1452.81539376	-0.043190833	0.47791200	-998.8	-1452.87945984
HCCHOH	-1452.89059888	-0.040719367	0.48644900	-	-1452.95463320
**R3**	U+D	D	ZPE	iν (cm-1)	U+D
H_2O + CCH	-1452.82257550	-0.04694502	0.48359800	-	-1452.88573796
TS1	-1452.81620564	-0.04450319	0.48287900	-331.9	-1452.87963184
H_2O–CCH	-1452.81719861	-0.04368832	0.48270400	-	-1452.88160226
TS2-1 (on C1)	-1452.81500405	-0.04418155	0.47809900	-876.8	-1452.87942323
TS2-2 (on C2)	-1452.81036985	-0.04434784	0.47871700	-1071.5	-1452.87509123
P1 (HCCHOH)	-1452.88698584	-0.04539624	0.48471300	-	-1452.95117273
P2 (H2CCOH)	-1452.89550893	-0.04324989	0.48448300	-	-1452.96039816

Table S10: ωB97X-D3-absolute energy values related to the potential energy surfaces for the reactivity of CCH on W33. tζ means 6-311++G(d,p), while tζ-ext means 6-311++G(2df,2pd); *opt* and *freq* indicate optimisation and frequency calculation (needed for ZPE calculation) and *sp* means single point energy calculation. U+D refers to the potential energy (U) plus the dispersion correction (D), and ZPE is the zero point energy correction. Energy units are in Hartree.

W33 ωB97X-D3	tζ (*opt* and *freq*)				tζ-ext (*sp*)
**R2–1**	U+D	D	ZPE	iν (cm-1)	U+D
R	-2599.70693151	-0.084287855	0.87738200	-	-2599.81485148
TS1	-2599.70512903	-0.084710125	0.87719200	-169.9	-2599.81386616
I	-2599.70990968	-0.086907833	0.87783500	-	-2599.82008303
TS2	-2599.70400278	-0.086037025	0.87118500	-1158.5	-2599.81460036
P	-2599.77119340	-0.083042623	0.87983500	-	-2599.88274900
**R2–2**	U+D	D	ZPE	iν (cm-1)	U+D
R	-2599.70107662	-0.0853605486	0.86582000	-	-2599.81057694
TS1	-2599.69947998	-0.0849067129	0.86524000	-178.2	-2599.80967406
I	-2599.70277889	-0.0847225810	0.86464700	-	-2599.81398591
TS2	-2599.69887363	-0.0853126006	0.85901700	-1129.0	-2599.80990082
P	-2599.77992087	-0.0896717627	0.86870200	-	-2599.89051938

Table S11: ωB97X-D3-relative energies (ΔE) of the three reactions studied on W18. Energies units are in kJ mol^{-1}. ΔU stands for the potential energy contribution and is listed at both ωB97X-D3/6-311++G(d,p), tζ, and ωB97X-D3/6-311++G(2df,2pd), tζ-ext, level of theories. ΔD is the dispersion correction and ΔZPE is the zero point energy correction contribution. ΔE = ΔU(tζ-ext) + ΔD + ΔZPE(tζ).

W18 ωB97X-D3	tζ (*opt* and *freq*)			tζ-ext (*sp*)	
**R1**	ΔU	ΔD	ΔZPE	ΔU	ΔE
R	0.0	0.0	0.0	0.0	0.0
TS1	24.1	-12.2	-12.3	21.8	-2.8
I	-78.4	-21.4	1.6	-79.3	-99.1
TS2	-59.3	-3.3	-2.0	-59.2	-64.5
P	-199.5	-10.3	10.8	-203.3	-202.8
**R2**	ΔU	ΔD	ΔZPE	ΔU	ΔE
R	0.0	0.0	0.0	0.0	0.0
TS1	2.8	-0.8	-0.7	1.5	0.0
I	-5.3	-2.7	2.1	-9.9	-10.5
TS2	12.6	-2.1	-15.7	7.2	-10.6
P	-191.3	4.4	6.7	-196.6	-185.5
**R3**	ΔU	ΔD	ΔZPE	ΔU	ΔE
R	0.0	0.0	0.0	0.0	0.0
TS1	10.3	6.4	-1.9	9.6	14.4
I	5.6	8.6	2.3	2.3	8.5
TS2-1	12.6	7.3	-14.4	9.3	2.1
P-1	-173.2	4.1	2.9	-175.9	168.9
TS2-2	25.2	6.8	-12.8	21.1	15.1
P-2	-201.2	9.7	2.3	-205.7	-193.7

Table S12: ωB97X-D3-relative energies (ΔE) of the three reactions studied on W33. Energies units are in kJ mol^{-1}. ΔU stands for the potential energy contribution and is listed at both ωB97X-D3/6-311++G(d,p), tζ, and ωB97X-D3/6-311++G(2df,2pd), tζ-ext, level of theories. ΔD is the dispersion contribution and ΔZPE is the zero point energy correction contribution. ΔE = ΔU(tζ-ext) + ΔD + ΔZPE(tζ).

W33 ωB97X-D3	tζ (*opt* and *freq*)			tζ-ext (*sp*)	
**R2–1**	ΔU	ΔD	ΔZPE	ΔU	ΔE
R	0.0	0.0	0.0	0.0	0.0
TS1	5.8	-1.1	-0.5	3.7	2.1
I	-0.9	-6.9	1.2	-6.9	-12.5
TS2	12.3	-4.6	-16.3	5.3	-15.6
P	-172.0	3.3	6.4	-181.5	-171.8
**R2–2**	ΔU	ΔD	ΔZPE	ΔU	ΔE
R	0.0	0.0	0.0	0.0	0.0
TS1	3.0	1.2	-1.5	1.2	0.8
I	-6.1	1.7	-3.1	-10.6	-12.0
TS2	5.7	0.1	-17.9	1.6	-16.1
P	-195.7	-11.3	7.6	-198.6	-202.3

IR spectra

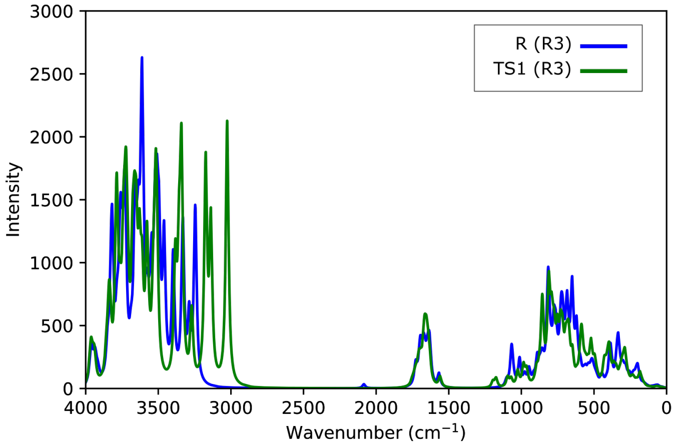

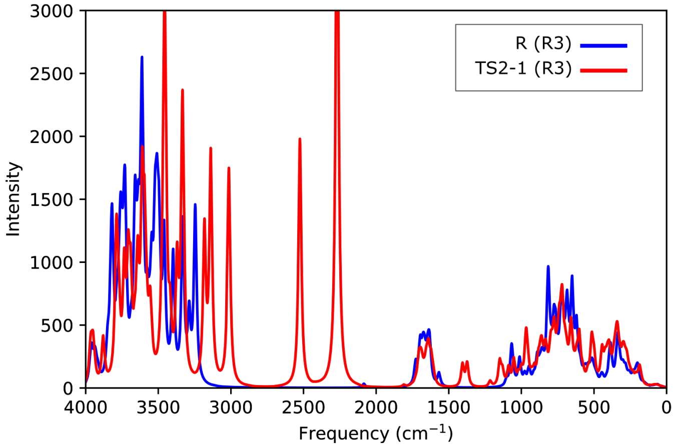

Figure S6: ωB97X-D3-simulated spectra of R, TS1 and TS2-1 structures of **R3** (W18 ice model).

Hydrogenation of vinyl alcohol

Table S13: Raw energetic data of the benchmarking study. Absolute potential energy values calculated at DFT were obtained by running DFT-D/6-311++G(2df,2pd)//DFT-D/6-311++G(d,p) optimizations + single points, see main text for more details. Reference values were obtained at CCSD(T)/aug-cc-PVTZ//mPWB1K-D3(BJ)/6-311++G(d,p). Energy units in Hartree.

**H'1**	BHLYP-D3(BJ)	MPWB1K-D3(BJ)	PW6B95-D3(BJ)	ωB97X-D3	CCSD(T)
R	-154.282720206	-154.284338934	-154.553355469	-154.327030565	-154.080318640
TS	-154.282611913	-154.282759514	-154.552482359	-154.322692502	-154.077858950
P	-154.356068817	-154.354172668	-154.620809898	-154.398660085	-154.147345110
**H'2**	BHLYP-D3(BJ)	MPWB1K-D3(BJ)	PW6B95-D3(BJ)	ωB97X-D3	CCSD(T)
R	-154.282720899	-154.284357654	-154.553370275	-154.327040444	-154.080266390
TS	-154.279687916	-154.278954351	-154.548626323	-154.318818975	-154.074198430
P	-154.342845464	-154.338836358	-154.620809898	-154.383806827	-154.134346310

Table S14: Unsigned error for each DFT method tested in the benchmarking study for the hydrogenation of vinyl alcohol with respect to CCSD(T). Values are in percentage.

**H'1**	BHLYP-D3(BJ)	MPWB1K-D3(BJ)	PW6B95-D3(BJ)	ωB97X-D3
CH_2CHOH + H	-	-	-	-
TS	54.2	11.9	23.6	38.5
CH_2CH_2OH	11.1	0.7	24.6	4.9
AVERAGE	32.7	6.3	24.1	21.7
**H'2**	BHLYP-D3(BJ)	MPWB1K-D3(BJ)	PW6B95-D3(BJ)	ωB97X-D3
CH_2CHOH + H	-	-	-	-
TS	116.4	43.6	78.5	93.0
CH_3CHOH	9.4	4.2	0.6	6.8
AVERAGE	62.9	23.9	39.6	49.9

Table S15: MPWB1K-D3(BJ)-absolute energy values relative to the potential energy surfaces for the hydrogenation of C_2CHOH on W18. tζ means 6-311++G(d,p), while tζ-ext means 6-311++G(2df,2pd); *opt* and *freq* indicate optimisation and frequency calculation (needed for ZPE calculation) and *sp* means single point energy calculation. U+D refers to the potential energy (U) plus the dispersion correction (D), and ZPE is the zero point energy correction. Energy units are in Hartree.

W18 MPWB1K-D3(BJ)	tζ (*opt* and *freq*)				tζ-ext (*sp*)
**H1**	U+D	D	ZPE	iν (cm-1)	U+D
R	-1453.653476	-0.025249893	0.508289000	-	-1453.717507
TS	-1453.650657	-0.025452985	0.508374000	-520.1	-1453.714791
P	-1453.724022	-0.025253836	0.516674000	-	-1453.787063
**H2**	U+D	D	ZPE	iν (cm-1)	U+D
R	-1453.653657	-0.025438836	0.507868000	-	-1453.717661
TS	-1453.648749	-0.026070842	0.509418000	-766.2	-1453.712603
P	-1453.710719	-0.025975019	0.487698000	-	-1453.773362

Table S16: MPWB1K-D3(BJ)-relative energies (ΔE) of the two reactions studied on W18. Energies units are in kJ mol^{-1}. ΔU stands for the potential energy contribution and is listed at both ωB97X-D3/6-311++G(d,p), tζ, and ωB97X-D3/6-311++G(2df,2pd), tζ-ext, level of theories. ΔD is the dispersion contribution and ΔZPE is the zero point energy correction contribution. ΔE = ΔU(tζ-ext) + ΔD + ΔZPE(tζ).

W18 MPWB1K-D3(BJ)	tζ (*opt* and *freq*)			tζ-ext (*sp*)	
**H1**	ΔU	ΔD	ΔZPE	ΔU	ΔE
R	0.0	0.0	0.0	0.0	0.0
TS	7.9	-0.5	0.2	7.7	7.4
P	-185.2	0.0	22.0	-182.6	-160.6
**H2**	ΔU	ΔD	ΔZPE	ΔU	ΔE
R	0.0	0.0	0.0	0.0	0.0
TS	14.5	-1.7	4.1	14.9	17.3
P	-148.4	-1.4	-53.0	-144.8	-199.2

Isomerization of vinyl alcohol and acetaldehyde

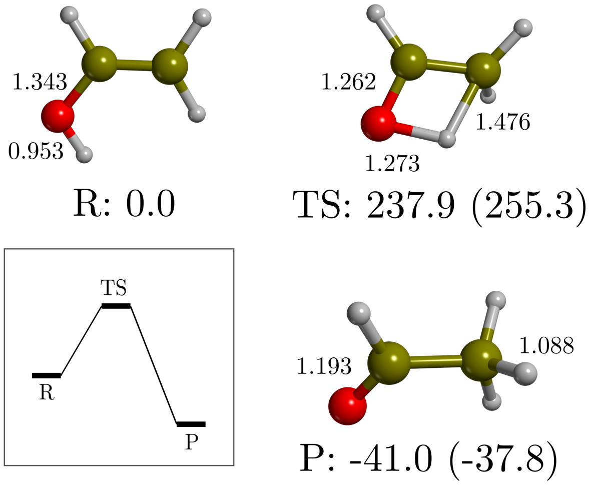

Figure S7: Potential energy surface (PES) of the gas phase isomerization between vinyl alcohol and acetaldehyde computed at MPWB1K-D3(BJ)/6-311++G(2df,2pd)//MPWB1K-D3(BJ)/6-311++G(d,p). Values in parenthesis correspond to relative potential energies, while bare values including ZPE corrections. The miniature panel sketches the ZPE-corrected PES. Energy units are in kJ mol^{-1} and distances in Å.

Table S17: MPWB1K-D3(BJ)-absolute energy values relative to the potential energy surfaces for the isomerization of C_2CHOH into CH_3CHO in the gas phase and on W18. tζ means 6-311++G(d,p), while tζ-ext means 6-311++G(2df,2pd); *opt* and *freq* indicate optimisation and frequency calculation (needed for ZPE calculation) and *sp* means single point energy calculation. U+D refers to the potential energy (U) plus the dispersion correction (D), and ZPE is the zero point energy correction. Energy units are in Hartree.

	tζ (*opt* and *freq*)				tζ-ext (*sp*)
**Gas phase**	U+D	D	ZPE	iν (cm-1)	U+D
R	-153.7775896	-0.0014761097	0.058059	-	-153.7882625
TS	-153.6805826	-0.0014709543	0.051464	-2284.1789	-153.6910395
P	-153.7934283	-0.0014348586	0.056821	-	-153.8026507
**W18**	U+D	D	ZPE	iν (cm-1)	U+D
R	-1453.166022	-0.0242819281	0.50837	-	-1453.229713
TS	-1453.128969	-0.0244099755	0.49974	-951.8493	-1453.193096
P	-1453.176748	-0.0249721724	0.508712	-	-1453.24004

Table S18: MPWB1K-D3(BJ)-relative energies (ΔE) for the conversion of CH_2CHOH into CH_3CHO in the gas phase and on W18. Energy units are in kJ mol^{-1}. ΔU stands for the potential energy contribution and is listed at both MPWB1K-D3(BJ)/6-311++G(d,p), tζ, and MPWB1K-D3(BJ)/6-311++G(2df,2pd), tζ-ext, level of theories. ΔD is the dispersion contribution and ΔZPE is the zero point energy correction contribution. ΔE = ΔU(tζ-ext) + ΔD + ΔZPE(tζ).

	tζ (*opt* and *freq*)			tζ-ext (*sp*)	
**Gas phase**	ΔU	ΔD	ΔZPE	ΔU	ΔE
R	0.0	0.0	0.0	0.0	0.0
TS	254.7	0.0	-17.3	255.2	237.9
P	-41.6	0.1	-3.3	-37.9	-41.0
**W18**	ΔU	ΔD	ΔZPE	ΔU	ΔE
R	0.0	0.0	0.0	0.0	0.0
TS	97.3	-0.3	-22.7	96.5	73.5
P	-28.2	-1.8	0.9	-25.3	-26.2

Optimized geometries

Benchmark

```
6
R'1_R
C 0.2320057019   -0.5612986126   0.2882722052
C 1.3115162435   -0.9008939456   0.7059133720
H 2.2383297202   -1.2606420675   1.0922373222
O -1.2012957676   1.0008638024   -0.7180370221
H -0.6024555369   1.1183022887   -1.4610926994
H -0.9605076979   1.6792192391   -0.0805508087

6
R'1_TS1
C 0.5007220667   -0.0644635280   0.3150397299
C 1.2971055421   0.1245192272   1.1963201201
H 2.0422643810   0.2616610247   1.9473948349
O -1.3635027060   -0.0971045663   -1.1393549267
H -0.3905896197   -0.3015613263   -0.7738441507
H -1.4319150528   0.8655806540   -1.0981022164

6
R'1_I
C 0.8634337094   -0.8132292399   -0.7449788492
C 1.3975775410   -0.0941376675   0.0507812170
H 1.8792389549   0.5440092325   0.7575567914
O -1.7831577050   0.7154202921   0.5475566902
H 0.3936406217   -1.4558241878   -1.4556976523
H -0.9113247578   0.3682519630   0.2789238476

6
R'1_TS2
C 0.5008210170   0.5494209826   0.2578675339
C 1.0125502635   -0.4363798377   0.7461606291
H 1.4043464285   -1.3457432593   1.1424794506
O -1.1704943472   -0.0356417664   -0.7840725442
H 0.2921784589   1.5560713025   -0.0290876589
H -1.1507859886   -0.9915684333   -0.6319354437

6
R'1_P
```

C 0.0673560115 0.4031125345 0.1900615599

C 0.0681440115 -0.3722734655 1.2454305599

H -0.0309429885 -1.4006524655 1.5535815599

O -0.0988999885 0.0300555345 -1.1187104401

H 0.2072810115 1.4777115345 0.2647045599

H -0.2211299885 -0.9215814655 -1.1665004401

6

R'2_R

C -1.00489306976824 0.987476420502441 -8.20676238346257E-02

C 0.24447652277248 1.02643363735407 0.163104441105061

H 1.28395065670452 1.17138385580714 0.379803974352455

O 0.495107986357984 -1.38661374113032 -6.12788247427841E-02

H 0.102913729035933 -1.49549610397159 -0.932244101336348

H -0.184550468051762 -1.66406812393288 0.559475063843509

6

R'2_TS

C -0.334635315796571 0.803080723797729 -0.155028788575873

C 0.843455628443605 1.06204168989808 0.134611806373561

H 1.83861588803412 1.38902074278698 0.360549346049056

O -0.44375426561689 -1.38945037306221 -4.96207261647306E-02

H -1.25643929673716 -1.32070262370604 0.471858306241658

H 0.39834049412699 -0.238569123177079 0.198475501907647

6

R'2_P

C -1.10599062541179E-02 -0.401904097015772 -4.84052591582391E-02

C 1.27613789311318 -0.138654760186467 1.40264376704644E-02

H 2.00568247158701 -0.918378558962077 -0.16196206072469

O -1.06700509761462 0.408672442273231 -1.24665816519944E-02

H -1.76755679074305 9.06266089353725E-03 0.510525086894991

H 1.62357040904802 0.863818438693302 0.258958651778685

6

R'3_R

C 0.2320057019 -0.5612986126 0.2882722052

C 1.3115162435 -0.9008939456 0.7059133720

H 2.2383297202 -1.2606420675 1.0922373222

O -1.2012957676 1.0008638024 -0.7180370221

H -0.6024555369 1.1183022887 -1.4610926994

H -0.9605076979 1.6792192391 -0.0805508087

```
6
R'3_TS
C -0.0454505962   0.6668116403   0.0954881310
C 1.1755114038   0.2739246403   0.1185521310
H 2.1548024038   0.7087796403   0.2224091310
O -0.9092885962   -0.6422153597   -0.2059768690
H -1.4204715962   -0.8420833597   0.5886241310
H 0.2328434038   -0.8826913597   -0.0921158690

6
R'3_P
C  -1.10599062541179E-02   -0.401904097015772   -4.84052591582391E-02
C  1.27613789311318   -0.138654760186467   1.40264376704644E-02
H  2.00568247158701   -0.918378558962077   -0.16196206072469
O  -1.06700509761462   0.408672442273231   -1.24665816519944E-02
H  -1.76755679074305   9.06266089353725E-03   0.510525086894991
H  1.62357040904802   0.863818438693302   0.258958651778685

8
H'1_R
C 0.9226612053   -0.8479074652   0.1358281645
C -0.1432820195   -0.1340758731   0.4364774608
H 1.5055762520   -0.6385431821   -0.7447787350
H 1.2202866183   -1.6556150292   0.7789024552
H -0.7337209395   -0.3381868813   1.3184307180
O -0.5671557944   0.8924223980   -0.3302274243
H -1.3485910467   1.2808455208   0.0441037455
H -0.9282443212   -1.1124101886   -2.9745572042

8
H'1_TS
C 1.2202827335   0.0266631424   0.0369595646
C -0.0447572898   0.1359465050   0.4223311381
H 1.4740228677   -0.1212092771   -0.9984038012
H 2.0076442114   0.2099207223   0.7443688750
H -0.3112660899   0.3302171182   1.4509773258
O -1.0671808531   -0.0225822764   -0.4364474709
H -1.8970150902   0.0382891670   0.0219455632
H 1.6581788191   -2.0363859862   0.2358313199

8
H'1_P
```

C 1.2676824660 -0.1376368784 -0.1055314218

C -0.0461245340 0.5206111216 -0.0378744218

H 1.2774154660 -0.8955538784 -0.8854594218

H 2.0439564660 0.5885331216 -0.3230604218

H -0.1586935340 1.4983501216 0.4096935782

O -1.0925935340 -0.3323968784 0.0944805782

H -1.9033435340 0.1580841216 0.1752455782

H 1.5270824660 -0.6368668784 0.8327225782

8

H'2_R

C 1.0302135316 0.7034172396 0.1709124051

C -0.1832114684 0.2278712396 0.3649924051

H 1.6042465316 0.4333292396 -0.6989115949

H 1.4594895316 1.3748842396 0.8915824051

H -0.7655524684 0.4944672396 1.2355544051

O -0.7767624684 -0.6179407604 -0.5030135949

H -1.6417304684 -0.8548827604 -0.1910115949

H 1.5800465316 -2.7365127604 0.3611624051

8

H'2_TS

C 1.2289350465 0.1915753808 0.2177139376

C 0.0405501567 0.1551719996 -0.3940714353

H 1.3314069537 -0.1140385335 1.2443393477

H 2.0836658028 0.5809535211 -0.3040360865

H -0.0518967508 0.3729604127 -1.4479100963

O -1.0249129075 -0.4252105637 0.1936574934

H -1.8188849074 -0.2092209720 -0.2827480533

H -0.4032149104 1.9867375434 -0.1820397505

8

H'2_P

C 1.0039435013 -0.7238611431 0.3620463345

C -0.3459211296 -0.4060019766 -0.1200929362

H 1.7704814042 0.0296427786 0.3222128160

H 1.2569828459 -1.7318436510 0.6403290185

H -1.0957066677 -0.8637575467 0.5339869147

O -0.5002868185 0.9876863792 -0.1689810859

H -1.3309539616 1.1958154177 -0.5812247395

H -0.5008212618 -0.8437493838 -1.1162030982

```
7
vinyl alc gas
C   1.144753    0.518251    0.004442
C   -0.122851    0.290751    0.296291
O   -0.905581   -0.673760   -0.214940
H   1.687561   -0.083964   -0.707552
H   -0.408993   -1.194111   -0.840824
H   -0.668135    0.890023    1.006769
H   1.666493    1.326122    0.483600

7
ts vinyl alc to acetaldehyde gas
C       -0.956284    3.430248   -1.499835
C       -2.318195    3.419453   -1.169715
O       -2.886934    2.426548   -1.701329
H       -0.813092    3.603298   -2.561915
H       -1.650976    2.189366   -1.894923
H       -2.894751    4.084616   -0.533110
H       -0.209853    3.894971   -0.875107

7
acetaldehyde gas
C  1.204126    0.364255   -0.050463
C -0.266407    0.293349    0.168290
O -0.953972   -0.630599   -0.142316
H  1.437152    1.233689   -0.661638
H  1.565150   -0.536920   -0.529998
H -0.715535    1.176548    0.654519
H  1.705139    0.507595    0.904624
```

W18

```
57
W18_R1_R
C -0.5219595184   4.5346086487   -0.1546181001
O 2.4296664816   2.5808416487   0.2820278999
O 1.2906594816   1.3543686487   2.4153658999
O 2.6337824816   -0.8835963513   2.3728308999
O 1.0376694816   -2.8627393513   1.4613868999
O 4.4626984816   1.1508306487   -0.9465011001
O 4.7695404816   -1.1007023513   0.4555448999
O 3.9323594816   -2.8262623513   -1.5613521001
O 1.4648884816   -1.8440623513   -1.1341861001
O 1.6572034816   0.8158586487   -1.7311451001
O -0.8103935184   0.8144216487   -2.6966991001
O -1.2789505184   -1.7061343513   -1.5578491001
O -3.5144355184   -3.1361373513   -0.9673531001
O -2.2410175184   2.2169436487   -0.7713891001
O -1.4491115184   1.3585796487   1.7368078999
O -3.1016645184   -0.5286223513   2.4846748999
O -1.7118485184   -2.4220883513   1.0988768999
O -4.7842255184   1.3613946487   -1.1145001001
O -4.8230355184   -0.9340663513   0.2828898999
H -5.6148535184   1.8200676487   -0.9863901001
H -4.8592285184   0.5055456487   -0.6348701001
H -4.3048545184   -0.8144023513   1.0955738999
H -4.4766475184   -1.7286873513   -0.1517571001
H 0.0712584816   -2.8287553513   1.5284798999
H 1.2124954816   -2.6020243513   0.5422838999
H 2.1462444816   3.5029336487   0.1984138999
H 2.0518004816   2.2577686487   1.1267648999
H -3.1947685184   2.0081886487   -0.8650911001
H -1.8030325184   1.7727686487   -1.5182091001
H -2.3678395184   -3.0263943513   0.7269348999
H -2.1905555184   -1.8514843513   1.7288458999
H -2.4856675184   0.2228936487   2.2722828999
H -3.3675515184   -0.4349753513   3.4003138999
H -2.7947035184   -2.7079133513   -1.4723001001
H -3.8937425184   -3.8201033513   -1.5205831001
H -1.2739555184   -0.8152773513   -1.9510371001
H -1.3103425184   -1.6051873513   -0.5887861001
H -0.4877295184   1.3283846487   1.8750348999
```

H -1.6351905184 1.6696116487 0.8309828999

H 0.1423654816 0.9014716487 -2.4391541001

H -0.8736565184 0.9958986487 -3.6359081001

H 3.3745804816 -0.9759453513 1.7535668999

H 2.0410564816 -1.6446993513 2.1880148999

H 3.9797204816 1.8046756487 -0.4112301001

H 3.8322184816 0.9550176487 -1.6517931001

H 4.7341634816 -0.2424423513 -0.0347211001

H 5.6317354816 -1.1466043513 0.8738218999

H 2.2206684816 -2.3287843513 -1.5175421001

H 0.6355654816 -2.0859183513 -1.5758041001

H 4.5610294816 -2.7589393513 -2.2807141001

H 4.3162644816 -2.3327983513 -0.8126561001

H 1.7939144816 0.4900056487 2.4095738999

H 1.3615344816 1.6994096487 3.3078278999

H 1.6573134816 -0.0950113513 -1.3701211001

H 1.7763764816 1.4341236487 -0.9864021001

H -1.3566645184 3.8734016487 -0.3868231001

C 0.6331984816 4.9673326487 0.0871638999

57

W18_R1_TS1

C 0.6355924725 3.8545496424 0.4366463906

O 3.5636674725 2.4308216424 1.4987883906

O 1.4651484725 0.7032346424 2.0202443906

O 2.4803314725 -1.6513823576 1.9431213906

O 0.4446314725 -3.1466883576 1.0706853906

O 4.3395294725 0.8925146424 -0.7904756094

O 4.4645524725 -1.6877613576 -0.1141856094

O 3.1525044725 -2.6858913576 -2.3425476094

O 0.9248744725 -1.6394813576 -1.2531796094

O 1.5380124725 0.9713336424 -1.0608636094

O -0.5400165275 1.7224586424 -2.6420816094

O -1.6148375275 -0.6663093576 -1.5707406094

O -3.6675945275 -2.3576573576 -1.1041346094

O -2.0063785275 2.7782926424 -0.5067816094

O -1.1463835275 0.6232006424 0.8729413906

O -3.0877085275 -0.4874393576 2.3832193906

O -2.3199395275 -3.0122313576 1.3499163906

O -4.6006595275 2.1034036424 -0.8176826094

O -4.9801985275 -0.3075953576 0.3132553906

H -5.4041225275 2.6121956424 -0.7068146094

H -4.7758825275 1.2147736424 -0.4315426094

H -4.4637115275 -0.3803443576 1.1317503906

H -4.7202975275 -1.0771763576 -0.2168216094

H -0.5096845275 -3.1670783576 1.2682783906

H 0.5157074725 -2.7284063576 0.1994223906

H 3.0048074725 3.3387816424 1.3444913906

H 2.8720894725 1.7834996424 1.7746163906

H -2.9755755275 2.6526686424 -0.5908426094

H -1.6379905275 2.5725356424 -1.3808736094

H -2.8195645275 -2.9843503576 0.5213973906

H -2.6424665275 -2.2569203576 1.8621993906

H -2.3487175275 -0.0129263576 1.9432173906

H -3.1529005275 -0.1577923576 3.2805083906

H -2.9658425275 -1.7762743576 -1.4644136094

H -3.9467265275 -2.9464973576 -1.8067076094

H -1.5138855275 0.1121526424 -2.1413566094

H -1.4620305275 -0.3258483576 -0.6654696094

H -0.2403925275 0.6675496424 1.2263463906

H -1.3862795275 1.5036526424 0.5000413906

H 0.3007724725 1.5521166424 -2.1619866094

H -0.3124715275 2.1120876424 -3.4876566094

H 3.1528994725 -1.7864123576 1.2566143906

H 1.7538414725 -2.2908933576 1.7611133906

H 4.3704084725 1.5273226424 -0.0601036094

H 3.4249074725 0.9773796424 -1.1105926094

H 4.5182014725 -0.7228153576 -0.3276446094

H 5.3378174725 -1.9602693576 0.1746423906

H 1.5745554725 -2.0799423576 -1.8366906094

H 0.0630994725 -1.5543983576 -1.6911706094

H 3.6446434725 -2.4173523576 -3.1193846094

H 3.7076334725 -2.4538473576 -1.5744856094

H 1.8591274725 -0.2295203576 1.9847063906

H 1.2218274725 0.8478406424 2.9386733906

H 1.3500244725 0.0057406424 -0.9896656094

H 1.3960514725 1.3526946424 -0.1854656094

H -0.3860735275 3.9991216424 0.1381923906

C 1.7848094725 3.7371876424 0.7849163906

57

W18_R1_I

C -2.5917491837 3.3592597527 0.4313017432

O -4.3729921837 1.8224417527 -1.4274692568

O -1.6999531837 1.1372847527 -1.6282222568

O -1.3835241837 -1.4293762473 -2.3242062568

O 0.9428888163 -2.4193062473 -1.1829652568

O -4.5236561837 -0.3793622473 0.3819397432

O -3.4889091837 -2.5663022473 -0.7349242568

O -2.4799081837 -3.6290602473 1.6481147432

O -0.3084061837 -2.1219262473 1.2180517432

O -1.8480741837 0.1268917527 0.9333797432

O -0.2197161837 1.8431227527 2.1640057432

O 1.6348188163 -0.2543972473 1.9273367432

O 4.1798018163 -1.0869102473 1.7957637432

O 0.4277508163 2.5669217527 -0.4761492568

O 1.6987888163 0.1438717527 -0.8207002568

O 4.0876098163 -0.1104122473 -2.0693912568

O 3.6571008163 -2.5365282473 -0.6629932568

O 3.0400078163 3.1779977527 -0.1905312568

O 4.8582948163 1.2186977527 0.2568267432

H 3.5526868163 3.9864747527 -0.1937592568

H 3.6647188163 2.4559317527 0.0456937432

H 4.8747268163 0.7772347527 -0.6064992568

H 4.7370278163 0.4975197527 0.8934477432

H 1.8830478163 -2.6657932473 -1.0688922568

H 0.5215318163 -2.5479912473 -0.3137692568

H -4.4535791837 2.5890827527 1.4679977432

H -3.4117741837 1.6998017527 -1.6172172568

H 1.3327388163 2.9426907527 -0.4839962568

H 0.2326388163 2.4364867527 0.4702837432

H 3.8690088163 -2.1845082473 0.2149197432

H 4.0178488163 -1.8789652473 -1.2757232568

H 3.1834298163 0.1085247527 -1.7580022568

H 4.1415768163 0.1324517527 -2.9947192568

H 3.2913628163 -0.8031782473 2.0964547432

H 4.6416018163 -1.4457802473 2.5552977432

H 1.1872548163 0.5290877527 2.2862407432

H 1.7037568163 -0.0763792473 0.9652607432

H 1.2271508163 -0.6801042473 -1.0712302568

H 1.1046898163 0.9137277527 -0.8797452568

H -0.9509031837 1.2596707527 1.8494957432

H -0.6159591837 2.5015027527 2.7382587432

H -2.0656971837 -1.8939442473 -1.8119292568

H -0.5549031837 -1.9076052473 -2.1631662568

H -4.6765471837 0.3560197527 -0.2361262568

H -3.6810941837 -0.1502632473 0.8128907432

H -3.9800431837 -1.7727992473 -0.3995812568

H -4.1096491837 -3.0716612473 -1.2643692568

H -0.9183311837 -2.7429432473 1.6598237432

H 0.3391228163 -1.7427222473 1.8318517432

H -3.1862091837 -3.5602552473 2.2919477432

H -2.8670091837 -3.3583922473 0.7942947432

H -1.4683981837 0.2816307527 -2.0556962568

H -0.9212391837 1.7115997527 -1.5875632568

H -1.3438351837 -0.7125662473 0.9234687432

H -1.8026321837 0.4911617527 0.0248457432

H -1.7098211837 3.6927377527 -0.0735512568

C -3.5751681837 2.9517967527 0.9832307432

57

W18_R1_TS2

C 0.7061307274 3.8637130983 -0.6424634835

O 2.5772127274 2.2822210983 0.4295625165

O 1.1000067274 1.7077500983 2.6290675165

O 2.1708087274 -0.6386189017 2.1716905165

O 0.6068987274 -2.8199519017 1.8129465165

O 4.5187057274 0.8794110983 -1.0735834835

O 4.4980187274 -1.2449809017 0.5665645165

O 3.7835777274 -3.1504709017 -1.3532904835

O 1.2611397274 -2.3485579017 -0.8576284835

O 1.7665117274 0.3013090983 -1.3285714835

O -0.5314482726 0.5438530983 -2.6413944835

O -1.3959362726 -1.8463889017 -1.4918004835

O -3.8587962726 -2.8342539017 -0.9025694835

O -1.8787182726 2.2902690983 -0.9276974835

O -1.5753422726 1.8182100983 1.7611945165

O -3.3153182726 -0.0331559017 2.3539245165

O -2.0590172726 -2.1546529017 1.1908155165

O -4.5078972726 1.8614820983 -1.4654694835

O -4.9029352726 -0.3459759017 0.0371765165

H -5.2696772726 2.4347840983 -1.3773424835

H -4.7135932726 1.0450260983 -0.9585094835

H -4.4311012726 -0.2383779017 0.8792705165

H -4.6460012726 -1.2134669017 -0.3111084835

H -0.3528362726 -2.6836729017 1.7842675165

H 0.8595717274 -2.8586069017 0.8759745165

H 2.8262817274 4.6653200983 -0.5333094835

H 2.0375537274 2.3831130983 1.2456735165

H -2.8408562726 2.2808530983 -1.1082194835

H -1.4906892726 1.6754770983 -1.5723294835

H -2.7740632726 -2.7072639017 0.8486215165

H -2.4886852726 -1.4588149017 1.7252945165

H -2.6566632726 0.6982820983 2.1933735165

H -3.6688802726 0.0882520983 3.2361355165

H -3.0578152726 -2.5801829017 -1.4027384835

H -4.3172912726 -3.5067349017 -1.4078964835

H -1.2496442726 -0.9979799017 -1.9469344835

H -1.4613112726 -1.6548359017 -0.5374564835

H -0.6420372726 1.7803880983 2.0283305165

H -1.6118422726 1.9936860983 0.8004485165

H 0.3816457274 0.5358630983 -2.2629994835

H -0.4602632726 0.7694510983 -3.5703334835

H 2.9565307274 -0.8533279017 1.6474475165

H 1.5849937274 -1.4272829017 2.1442125165

H 4.2589127274 1.6536510983 -0.5577614835

H 3.6848667274 0.6339060983 -1.5086144835

H 4.6358567274 -0.4378159017 0.0151465165

H 5.2805047274 -1.3508779017 1.1116195165

H 2.0124817274 -2.8149399017 -1.2677434835

H 0.4448147274 -2.4786129017 -1.3653474835

H 4.3710747274 -3.0188499017 -2.0984044835

H 4.1122877274 -2.5700019017 -0.6425814835

H 1.4976687274 0.8034230983 2.5195545165

H 1.2380427274 1.9661550983 3.5419105165

H 1.6377877274 -0.6001459017 -0.9666474835

H 1.8411847274 0.9280130983 -0.5839624835

H -0.3081692726 3.5039930983 -0.7179334835

C 1.8482787274 4.2433260983 -0.5624944835

57

W18_R1_P

C 0.7029638835 3.6310560900 -1.1080446112

O 2.7715038835 2.9745390900 -0.0499396112

O 1.4147688835 1.9325190900 2.1544573888

O 2.1209318835 -0.6490589100 2.1738703888

O 0.7785198835 -2.9201599100 1.6278363888

O 3.9179608835 0.5835920900 -0.9574036112

O 4.5671258835 -1.1997949100 0.9254013888

O	3.8985188835	-2.3080269100	-1.6807276112
O	1.2593288835	-2.7614779100	-1.1628056112
O	1.3062178835	-0.1411089100	-0.4617016112
O	-0.5987301165	0.8341630900	-2.1039736112
O	-1.3392861165	-1.8491599100	-1.7917626112
O	-3.7932981165	-2.7751159100	-1.0545216112
O	-2.2506311165	2.5064990900	-0.5642506112
O	-1.4441081165	1.8485450900	1.9246093888
O	-2.9104441165	-0.2675679100	2.3739953888
O	-1.8309681165	-2.2960729100	0.9276293888
O	-4.8859031165	1.9313820900	-1.0092276112
O	-4.8163641165	-0.4532329100	0.2707723888
H	-5.6859411165	2.4046730900	-0.7801436112
H	-4.9479281165	1.0512000900	-0.5800056112
H	-4.2174701165	-0.3737769100	1.0302203888
H	-4.5414881165	-1.2467199100	-0.2135536112
H	-0.1856491165	-2.8306129100	1.5412713888
H	1.0615888835	-3.1066349100	0.7198603888
H	2.6379988835	3.8616800900	-1.8715236112
H	2.2130488835	2.6614730900	0.6889123888
H	-3.2150291165	2.4520540900	-0.7116116112
H	-1.8522991165	1.8675610900	-1.1760676112
H	-2.5868181165	-2.8018919100	0.5997363888
H	-2.1995041165	-1.6065539100	1.5165293888
H	-2.3282751165	0.5338530900	2.2633273888
H	-3.1866831165	-0.2912179100	3.2915113888
H	-3.0367921165	-2.4994419100	-1.6095076112
H	-4.3233091165	-3.3862289100	-1.5680336112
H	-1.2117971165	-0.9451399100	-2.1263246112
H	-1.3164511165	-1.7710139100	-0.8198966112
H	-0.4765101165	1.8769460900	1.9390753888
H	-1.7323061165	2.1585850900	1.0375503888
H	0.1160898835	0.6726320900	-1.4529226112
H	-0.2364361165	1.4603680900	-2.7360026112
H	3.0287308835	-0.8983209100	1.9261153888
H	1.5995458835	-1.4858329100	2.1552423888
H	3.9013768835	1.5270030900	-0.7388716112
H	2.9684858835	0.3490170900	-0.9185186112
H	4.5247628835	-0.4302719100	0.3130293888
H	5.4460078835	-1.2200529100	1.3080553888
H	2.1157168835	-2.7964419100	-1.6270436112

```
H 0.5189428835   -2.7969219100   -1.7837586112
H 4.1143278835   -1.4334669100   -2.0224226112
H 4.1832158835   -2.2664289100   -0.7559116112
H 1.6939058835   1.0035000900   2.3201413888
H 1.5849868835   2.4205070900   2.9634803888
H 1.2463648835   -1.0918739100   -0.7044926112
H 1.4268128835   -0.1572959100   0.5036513888
H -0.2487871165   3.4997670900   -0.6127626112
C 2.0053318835   3.5148930900   -1.0561386112

57
w18_r2_r
C  -1.57525464312044   3.93995209985251   -1.13947598524508
O  2.01566735687956   2.75275109985251   0.372649014754921
O  1.17139735687956   1.74760909985251   2.80377901475492
O  2.33748335687956   -0.356710900147492   1.75825201475492
O  1.29156335687956   -2.86298090014749   1.70201401475492
O  4.19580835687956   1.74892209985251   -0.994680985245079
O  4.86635235687956   -0.383203900147492   0.439126014754921
O  4.40558435687956   -2.25314790014749   -1.58066298524508
O  1.77128835687956   -2.28617490014749   -0.965878985245079
O  1.63233435687956   0.479402099852508   -1.16243398524508
O  -0.680072643120439   0.556757099852508   -2.38896198524508
O  -0.97073664312044   -2.10411690014749   -1.57080098524508
O  -3.29187664312044   -3.33136690014749   -0.887756985245079
O  -2.22172564312044   1.99095909985251   -0.674947985245079
O  -1.51636264312044   1.63611609985251   2.01999001475492
O  -2.88409564312044   -0.563229900147492   2.39508901475492
O  -1.45370864312044   -2.53127790014749   1.14339701475492
O  -4.73301064312044   1.27928109985251   -1.17178498524508
O  -4.55438264312044   -1.01960890014749   0.169124014754921
H  -5.55230664312044   1.71366509985251   -0.931885985245079
H  -4.74435164312044   0.396495099852508   -0.730932985245079
H  -4.03099964312044   -0.867754900147492   0.973960014754921
H  -4.21663164312044   -1.83707690014749   -0.230237985245079
H  0.322368356879561   -2.87304690014749   1.70030601475492
H  1.51763735687956   -2.90653490014749   0.758222014754921
H  1.31742835687956   3.30655409985251   -4.02399852450794E-02
H  1.78352435687956   2.67447009985251   1.31171001475492
H  -3.18275064312044   1.83970809985251   -0.856287985245079
H  -1.71696464312044   1.45855709985251   -1.32796098524508
H  -2.13387764312044   -3.14107790014749   0.828951014754921
```

H -1.91951064312044 -1.86886990014749 1.68975401475492

H -2.35686464312044 0.277453099852508 2.29896701475492

H -3.22672464312044 -0.576195900147492 3.29026001475492

H -2.53325664312044 -2.99189390014749 -1.40555298524508

H -3.68853764312044 -4.04723390014749 -1.38585198524508

H -0.99778864312044 -1.21011890014749 -1.95449398524508

H -0.96513164312044 -1.98691690014749 -0.602319985245079

H -0.57708964312044 1.71722509985251 2.26144301475492

H -1.61871364312044 1.89770909985251 1.09112101475492

H 0.22952435687956 0.606902099852508 -1.98516298524508

H -0.61471364312044 0.920647099852508 -3.27415398524508

H 3.24513235687956 -0.446205900147492 1.43023801475492

H 1.96278135687956 -1.26052990014749 1.84513401475492

H 3.65160335687956 2.34406609985251 -0.447878985245079

H 3.51668435687956 1.29071109985251 -1.51001198524508

H 4.73465335687956 0.475129099852508 -3.96599852450794E-02

H 5.69433735687956 -0.318600900147492 0.918539014754921

H 2.61856835687956 -2.44942790014749 -1.41949298524508

H 1.02307135687956 -2.49359890014749 -1.54252898524508

H 4.89432035687956 -1.93981090014749 -2.34257298524508

H 4.65225935687956 -1.66641490014749 -0.841619985245079

H 1.63948735687956 0.921765099852508 2.51916201475492

H 1.38300135687956 1.89474809985251 3.72651901475492

H 1.73154635687956 -0.432962900147492 -0.830264985245079

H 1.68058335687956 1.08767109985251 -0.399955985245079

H -2.53299564312044 4.28027009985251 -1.47713498524508

C -0.34802464312044 3.90762509985251 -0.822387985245079

57

w18_r2_ts1

C -1.72419583246208 3.61175807753748 -1.13988762250478

O 1.96865816753792 2.77221107753748 0.251094377495225

O 1.31063016753792 1.86538207753748 2.79305937749522

O 2.41141116753791 -0.27318092246252 1.74373137749523

O 1.36515616753792 -2.78418792246252 1.79512637749522

O 4.08885416753792 1.76674707753748 -1.17956562250478

O 4.87955716753792 -0.27497792246252 0.319524377495225

O 4.40254716753792 -2.24399292246252 -1.59065062250478

O 1.78633516753792 -2.31804892246252 -0.902740622504775

O 1.53381016753791 0.43941707753748 -1.15396562250477

O -0.786659832462085 0.44711407753748 -2.32901162250478

O -0.968507832462085 -2.22241092246252 -1.49201962250478

O	-3.29367683246209	-3.37894592246252	-0.706097622504775
O	-2.30997483246209	1.94108707753748	-0.742831622504775
O	-1.35553883246208	1.75743907753748	2.01062237749522
O	-2.78361083246209	-0.41235492246252	2.36119337749522
O	-1.39608483246208	-2.49395192246252	1.24801937749522
O	-4.78132483246208	1.23763807753748	-1.21067962250478
O	-4.49967583246208	-0.98487292246252	0.206931377495225
H	-5.59575083246208	1.68047507753748	-0.968979622504775
H	-4.77496883246208	0.36905307753748	-0.738556622504775
H	-3.94812683246209	-0.78395692246252	0.982176377495225
H	-4.18428683246208	-1.83226292246252	-0.146613622504775
H	0.396650167537915	-2.79915092246252	1.81307037749523
H	1.57220016753792	-2.86477692246252	0.849286377495225
H	1.20729716753791	3.26565507753748	-0.142158622504775
H	1.81766816753791	2.75209507753748	1.20808037749522
H	-3.28105783246209	1.79661107753748	-0.923771622504775
H	-1.79249083246208	1.35334707753748	-1.35045762250478
H	-2.09033583246209	-3.11470392246252	0.991655377495225
H	-1.84450383246209	-1.78218692246252	1.74451137749523
H	-2.23532783246209	0.41080107753748	2.24580837749522
H	-3.13067983246209	-0.38567692246252	3.25464837749522
H	-2.53505383246209	-3.08622192246252	-1.25181062250478
H	-3.70599383246209	-4.11960392246252	-1.15272062250478
H	-1.02489383246208	-1.33993392246252	-1.89723462250478
H	-0.938247832462085	-2.07468592246252	-0.527755622504775
H	-0.421647832462085	1.84042407753748	2.27700637749522
H	-1.44125683246208	2.10134107753748	1.11313137749522
H	0.125876167537915	0.51843007753748	-1.92413962250478
H	-0.723861832462085	0.80702607753748	-3.21662762250478
H	3.30449216753791	-0.36334892246252	1.37786237749523
H	2.04552816753791	-1.17524092246252	1.87139537749523
H	3.54611716753791	2.36842707753748	-0.636180622504775
H	3.40633516753792	1.27578007753748	-1.65753162250478
H	4.69567316753792	0.55507707753748	-0.192293622504775
H	5.72204516753792	-0.15625692246252	0.761878377495225
H	2.63412316753791	-2.46618792246252	-1.36160662250478
H	1.04206416753791	-2.56237092246252	-1.46944962250478
H	4.85854016753792	-1.96222592246252	-2.38452162250478
H	4.66387816753792	-1.61635592246252	-0.891026622504775
H	1.76243416753792	1.03199907753748	2.50445637749522
H	1.56495016753791	2.03328307753748	3.70121137749522

H 1.68204716753791 -0.45507192246252 -0.794575622504775

H 1.62093016753792 1.08435307753748 -0.423976622504775

H -2.63078583246209 4.05295907753748 -1.51521162250478

C -0.484618832462085 3.73512107753748 -0.848705622504775

57

w18_r2_i

C 2.07277406154812 -3.14999650743472 -1.10323212072869

O -1.82992693845188 -2.79018750743472 8.21387927131189E-03

O -1.64609293845188 -2.09955050743472 2.71276387927131

O -2.62288693845188 0.102257492565283 1.66364687927131

O -1.54047893845188 2.57961149256528 2.00349887927131

O -3.82441793845188 -1.75588050743472 -1.55971012072869

O -4.92904593845188 8.84304925652835E-02 -5.09812072868811E-03

O -4.37560693845188 2.26340649256528 -1.63624012072869

O -1.81930193845188 2.39159149256528 -0.746960120728688

O -1.36177393845188 -0.326381507434717 -1.12179812072869

O 0.962769061548116 -0.203616507434717 -2.22397312072869

O 0.954655061548116 2.44011349256528 -1.25145112072869

O 3.22245906154812 3.48241049256528 -0.181650120728688

O 2.56574106154812 -1.70849850743472 -0.871946120728688

O 1.02718406154812 -2.09267350743472 1.93798187927131

O 2.59095206154812 -1.49705074347166E-02 2.20194487927131

O 1.23695806154812 2.26143949256528 1.50543587927131

O 4.94680606154812 -0.947928507434717 -1.39712412072869

O 4.40554106154812 0.967671492565283 0.313493879271312

H 5.76651806154812 -1.40327250743472 -1.19939412072869

H 4.89396106154812 -0.155878507434717 -0.804076120728688

H 3.78955406154812 0.597550492565283 0.970849879271312

H 4.10084506154812 1.86837849256528 0.116432879271312

H -0.574592938451884 2.53931549256528 2.06414187927131

H -1.69626693845188 2.76510449256528 1.06282087927131

H -0.960211938451884 -3.16630650743472 -0.292220120728688

H -1.85101993845188 -2.87117150743472 0.972523879271312

H 3.53308706154812 -1.54304650743472 -1.11255812072869

H 1.96778106154812 -1.08337550743472 -1.38810612072869

H 1.94545406154812 2.91104949256528 1.41162587927131

H 1.66233306154812 1.45191449256528 1.85182287927131

H 2.00716406154812 -0.806250507434717 2.03302887927131

H 2.95087406154812 -0.146646507434717 3.08152087927131

H 2.49154206154812 3.28492349256528 -0.802257120728688

H 3.63925906154812 4.29547749256528 -0.470372120728688

H 1.07867206154812 1.60820049256528 -1.73715412072869

H 0.874656061548116 2.18115949256528 -0.312570120728688

H 0.116925061548116 -2.08073150743472 2.28382087927131

H 0.994973061548116 -2.60500650743472 1.11267587927131

H 5.02870615481157E-02 -0.318170507434717 -1.81208512072869

H 0.893841061548116 -0.523472507434717 -3.12707412072869

H -3.47738193845188 0.209610492565283 1.21959487927131

H -2.27574793845188 0.991657492565283 1.89010587927131

H -3.30049693845188 -2.37689550743472 -1.01765112072869

H -3.12957493845188 -1.18418150743472 -1.91301512072869

H -4.62106093845188 -0.679607507434717 -0.553845120728688

H -5.80011093845188 -0.132012507434717 0.329662879271312

H -2.65337793845188 2.52614749256528 -1.23545412072869

H -1.06829893845188 2.70667649256528 -1.26762512072869

H -4.75355993845188 2.07363149256528 -2.49565412072869

H -4.67542693845189 1.54869249256528 -1.04321612072869

H -2.06907293845188 -1.25294750743472 2.42321987927131

H -1.99731493845188 -2.32118150743472 3.57565087927131

H -1.59908393845188 0.530106492565283 -0.720993120728688

H -1.49183493845188 -1.03305550743472 -0.456712120728688

H 2.90515206154812 -3.71679250743472 -1.50872112072869

C 0.835598061548116 -3.42066750743472 -0.791150120728688

57

w18_r2_ts2

C 1.64943507391603 -3.19511359323598 -1.02019736405548

O -2.04887392608397 -2.46599659323598 0.043051635944521

O -1.71033692608397 -1.81708559323598 2.72955163594452

O -2.47668292608397 0.446954406764017 1.63389263594452

O -1.19668992608397 2.82568840676402 1.98388263594452

O -4.50159992608397 -2.01434459323598 -0.895776364055479

O -4.73605892608397 0.524465406764018 -0.114824364055479

O -3.97190492608397 2.50465240676402 -1.89604836405548

O -1.51728892608397 2.49850440676402 -0.753658364055479

O -1.40707792608397 -0.213421593235982 -1.12801036405548

O 0.773690073916026 -0.335028593235983 -2.11473036405548

O 1.21975007391603 2.29595040676402 -1.24104836405548

O 3.58258207391603 3.20724940676402 -0.243949364055479

O 2.21791107391603 -1.84591659323598 -0.951066364055479

O 0.959528073916026 -2.16549159323598 1.97669663594452

O 2.66436307391603 -0.193478593235982 2.18399363594452

O 1.53412007391603 2.19328740676402 1.50197463594452

O 4.70094907391603 -1.33142159323598 -1.61886936405548

O 4.52922607391603 0.570336406764018 0.228469635944521

H 5.48703207391603 -1.86769459323598 -1.50883036405548

H 4.77344907391603 -0.578333593235983 -0.985620364055479

H 3.89379707391603 0.259582406764018 0.896985635944521

H 4.31397607391603 1.49929740676402 0.051723635944521

H -0.239795926083974 2.68329840676402 2.04138063594452

H -1.34262592608397 2.99841640676402 1.04002363594452

H -1.20093392608397 -2.94018859323598 -0.297457364055479

H -2.01533292608397 -2.50424259323598 1.01229663594452

H 3.16749407391603 -1.80769459323598 -1.23476736405548

H 1.50806507391603 -1.07204659323598 -1.53870636405548

H 2.29990707391603 2.77076840676402 1.38708963594452

H 1.88618907391603 1.34867440676402 1.85066563594452

H 2.02323307391603 -0.942112593235983 2.02876563594452

H 3.04470507391603 -0.360092593235982 3.04889963594452

H 2.84329307391603 3.05092540676402 -0.863893364055479

H 4.07773707391603 3.96217940676402 -0.564955364055479

H 1.23086907391603 1.43602240676402 -1.69283536405548

H 1.17112207391603 2.08637640676402 -0.286470364055479

H 5.35140739160258E-02 -2.04876259323598 2.30998663594452

H 0.881764073916026 -2.66563759323598 1.14646763594452

H -0.306399926083974 -0.313663593235982 -1.61357436405548

H 0.684986073916026 -0.655264593235983 -3.01658536405548

H -3.29660692608397 0.610567406764018 1.14472863594452

H -2.06800392608397 1.30944940676402 1.86225663594452

H -3.61937492608397 -2.32861359323598 -0.587929364055479

H -4.61869092608397 -2.36051159323598 -1.78110736405548

H -4.67883992608397 -0.401664593235982 -0.457416364055479

H -5.59120692608397 0.594454406764018 0.315042635944521

H -2.29625192608397 2.69170440676402 -1.31002536405548

H -0.703432926083974 2.71246440676402 -1.23401936405548

H -4.20209592608397 2.24614840676402 -2.78916136405548

H -4.31694092608397 1.80291240676402 -1.31163936405548

H -2.04689792608397 -0.932797593235983 2.44332863594452

H -2.11389292608397 -2.02781959323598 3.57204963594452

H -1.54205892608397 0.655138406764017 -0.695497364055479

H -1.66496592608397 -0.973792593235983 -0.532594364055479

H 2.42814007391603 -3.91723859323598 -1.26692436405548

C 0.370490073916026 -3.38285759323598 -0.791645364055479

57

w18_r2_p

C -2.01153807329594 3.31526834505501 -0.629548581173248

O 2.49326492670406 2.77425734505501 5.72684188267519E-02

O 1.78030092670406 1.49288234505501 2.51779341882675

O 2.31449892670406 -0.756907654944988 1.23987641882675

O 1.25111692670406 -3.16717665494499 1.43042741882675

O 5.07494192670406 1.98735034505501 -0.213709581173248

O 4.87422192670406 -0.669193654944988 0.162019418826752

O 3.30814392670406 -1.53872965494499 -2.08211558117325

O 1.41870092670406 -3.46835865494499 -1.29654958117325

O 1.56817092670406 0.473544345055012 -1.08592058117325

O -0.851758073295941 0.451601345055012 -2.44781358117325

O -1.10967507329594 -2.07192465494499 -1.31103258117325

O -3.50185407329594 -3.04714565494499 -0.518217581173248

O -2.40823907329594 2.47274334505501 -1.63388458117325

O -0.931646073295941 1.71350634505501 2.13077341882675

O -2.62388007329594 -0.255266654944988 2.60595041882675

O -1.49314707329594 -2.46988065494499 1.41890541882675

O -4.76670007329594 1.69116634505501 -0.852276581173248

O -4.44784607329594 -0.577459654944988 0.486810418826752

H -5.65701107329594 1.77633534505501 -1.19288958117325

H -4.70452707329594 0.824844345055012 -0.381055581173248

H -3.87113307329594 -0.465243654944988 1.26084541882675

H -4.22552307329594 -1.43813865494499 9.66644188267519E-02

H 0.296059926704059 -3.09613165494499 1.57594241882675

H 1.34657792670406 -3.43303065494499 0.491530418826752

H 0.265234926704059 3.55973834505501 -0.672425581173248

H 2.25856492670406 2.58681834505501 0.979183418826752

H -3.34537007329594 2.20157734505501 -1.46489258117325

H -1.42644507329594 1.20451934505501 -2.18463958117325

H -2.26587107329594 -2.96787065494499 1.11982241882675

H -1.83875307329594 -1.74866265494499 1.97577741882675

H -2.00244107329594 0.496363345055012 2.43262441882675

H -2.93341807329594 -0.152423654944988 3.50763241882675

H -2.72425807329594 -2.80853965494499 -1.06480258117325

H -3.95892207329594 -3.76622965494499 -0.956471581173248

H -1.10137907329594 -1.18959065494499 -1.72025758117325

H -1.01624107329594 -1.94629965494499 -0.349790581173248

H 1.85789267040588E-02 1.64725634505501 2.34158341882675

H -0.996739073295941 2.29177734505501 1.35937641882675

H 0.683084926704059 0.431418345055012 -1.48390458117325

H -0.702857073295941 0.532083345055012 -3.39232858117325

H 3.24200292670406 -0.863136654944988 0.958991418826752

H 1.94092792670406 -1.66410965494499 1.40055841882675

H 4.18017592670406 2.38510034505501 -0.113243581173248

H 5.45378092670406 2.37417234505501 -1.00423658117325

H 5.02145292670406 0.292700345055012 5.40141882675191E-03

H 5.66919792670406 -1.00698965494499 0.577652418826752

H 2.12581392670406 -2.88850665494499 -1.63623558117325

H 0.587753926704059 -3.05370765494499 -1.56684458117325

H 2.76940692670406 -0.741901654944988 -1.95917158117325

H 4.01681992670406 -1.43010465494499 -1.43244058117325

H 2.02345292670406 0.601349345055012 2.16370041882675

H 2.21535692670406 1.59090734505501 3.36629941882675

H 1.58628492670406 -7.04966549449883E-02 -0.273652581173248

H 2.11834092670406 2.01861234505501 -0.437396581173248

H -2.83524407329594 3.70054434505501 -2.61485811732481E-02

C -0.769839073295941 3.65214534505501 -0.373837581173248

57

w18_v3_R

C 2.5544821822 3.0337298281 -2.2648324608

C 2.0419641822 2.2644088281 -1.4880914608

O 1.0531381822 0.4374158281 -0.4075314608

O -0.9525448178 1.7110278281 -1.8697164608

O 1.2264721822 2.0532198281 1.7176915392

O -1.3266348178 2.6950908281 0.6367765392

O -2.6511678178 -0.0487181719 -2.8486484608

O 3.1002891822 -0.5660641719 -2.0513904608

O 3.8455751822 1.8475888281 1.1182755392

O 4.4555381822 -0.7418831719 0.3955755392

O -0.6799948178 -1.5399621719 -1.7355014608

O 1.7265331822 -2.9771681719 -1.6901224608

O -4.1383808178 1.8065748281 0.8602095392

O -4.7974658178 -0.0922191719 -0.9405434608

O -4.3563668178 -2.5240851719 0.3408455392

O -1.8323198178 -1.7552331719 0.8146815392

O -1.9818588178 0.5673168281 2.2211235392

O 0.2698931822 0.1933208281 3.5723585392

O 0.8418851822 -1.6786481719 1.5571365392

O 2.9933291822 -3.1434451719 0.9070525392

H 3.9940621822 2.2828498281 0.2744905392

H 4.0845831822 0.9085368281 0.9692185392

H 4.2246901822 -0.7531061719 -0.5459874608
H 4.1171261822 -1.5854461719 0.7304665392
H 0.0860361822 -2.1410421719 -1.7760924608
H -1.0823008178 -1.6733201719 -0.8631494608
H -0.4493078178 2.9147448281 0.9832785392
H -1.1924558178 2.4643598281 -0.3038584608
H 2.2048641822 2.1026168281 1.6251035392
H 1.0485481822 1.5497048281 2.5286895392
H 2.1618621822 -3.2338291719 -0.8645044608
H 2.3164931822 -2.3113131719 -2.0717674608
H 2.3885641822 -0.1817401719 -1.5084184608
H 3.2753851822 0.0874288281 -2.7325744608
H 2.2112341822 -2.7299761719 1.3298545392
H 3.2138361822 -3.9305291719 1.4073405392
H 0.7916121822 -1.0955341719 2.3357325392
H 0.9166911822 -1.0817191719 0.7913165392
H 0.2422561822 0.5734348281 -0.9406444608
H 1.0286001822 1.0652158281 0.3576805392
H -0.6582068178 0.3363938281 3.2621655392
H 0.2616431822 0.1507388281 4.5296775392
H -3.4103058178 -0.1739021719 -2.2592684608
H -2.0351428178 -0.7823031719 -2.6637634608
H -3.4849968178 2.4802958281 0.6321985392
H -3.7136198178 1.3374718281 1.5950035392
H -4.5696958178 0.6439618281 -0.3240464608
H -5.6357178178 0.1459908281 -1.3422544608
H -2.6590788178 -2.2758171719 0.7858085392
H -1.1142898178 -2.2396841719 1.2432075392
H -5.0901758178 -2.7374881719 0.9180485392
H -4.6077138178 -1.7137691719 -0.1407954608
H -1.6383498178 1.1338428281 -2.2988794608
H -0.4717558178 2.1678358281 -2.5639744608
H -1.9857598178 -0.2504091719 1.6806615392
H -1.6828858178 1.2833018281 1.6206015392
H 3.0004291822 3.7460728281 -2.9228224608

57

w18_v3_TS1

C 1.9203015042 3.7843881496 -0.2203898494
C 2.3406875042 2.6221241496 -0.4979798494
O 1.1193265042 1.4262731496 -0.5691928494
O -1.1259054958 2.8121131496 -0.8599698494

```
O 1.1538655042   0.7639501496   1.9811301506
O -1.3971204958   1.9725561496   1.7533891506
O -2.6788444958   1.3666611496   -2.3239838494
O 3.8266495042   0.6911601496   -1.8586478494
O 3.7884795042   1.1042711496   1.8536121506
O 4.6255855042   -0.9824978504   0.2384781506
O -0.9270164958   -0.6878578504   -2.5000298494
O 1.7678535042   -1.2150468504   -1.7546848494
O -4.2355354958   1.0356541496   1.7734251506
O -4.8267514958   0.3081781496   -0.7515898494
O -4.4639664958   -2.4767418504   -0.7904458494
O -1.8361354958   -2.1839768504   -0.3208328494
O -2.0405134958   -0.6852728504   1.9277031506
O 0.2061975042   -1.6885188504   2.9055881506
O 0.8024995042   -2.7039198504   0.3694461506
O 3.2574515042   -3.3788638504   -0.6003918494
H 3.7908575042   1.7916991496   1.1755081506
H 4.1399955042   0.3081851496   1.4060681506
H 4.5301225042   -0.4946128504   -0.5939958494
H 4.2287705042   -1.8499988504   0.0724021506
H 0.0377445042   -0.7541018504   -2.4434308494
H -1.2528164958   -1.2570248504   -1.7839148494
H -0.4843894958   1.9043331496   2.0745921506
H -1.3371794958   2.4173571496   0.8892231506
H 2.1272335042   0.9031251496   2.0972451506
H 0.9478735042   -0.0965568504   2.3839971506
H 2.3457045042   -1.9744818504   -1.9034328494
H 2.3576355042   -0.4405408504   -1.7924418494
H 3.4510185042   1.4863261496   -1.3898698494
H 4.2199185042   1.0100681496   -2.6728828494
H 2.4068925042   -3.4214218504   -0.1220618494
H 3.6167345042   -4.2664628504   -0.6321948494
H 0.7286465042   -2.4013758504   1.2908781506
H 1.0249715042   -1.9255818504   -0.1758708494
H 0.2677735042   1.9124161496   -0.7359248494
H 1.0774995042   1.0550621496   0.3575861506
H -0.7251944958   -1.4057008504   2.7383601506
H 0.2621935042   -2.0488208504   3.7917181506
H -3.4275344958   0.9602871496   -1.8600438494
H -2.0552674958   0.6383581496   -2.5329908494
H -3.5959354958   1.7465791496   1.9051881506
```

H -3.7367584958   0.2500251496   2.0518701506

H -4.6535744958   0.6497991496   0.1585011506

H -5.6696404958   0.6785051496   -1.0226228494

H -2.6872654958   -2.6258198504   -0.4976558494

H -1.1198174958   -2.8139808504   -0.1505028494

H -5.1815864958   -2.8795818504   -0.3006018494

H -4.6543574958   -1.5211608504   -0.8203518494

H -1.7861514958   2.3369421496   -1.4499528494

H -0.9575684958   3.6845191496   -1.2225068494

H -2.0242564958   -1.1729088504   1.0763711506

H -1.7461224958   0.2284101496   1.7330011506

H 2.1226855042   4.8233911496   -0.0470978494

57

w18_v3_I

C 0.8818746177   2.5764373356   -2.4847241128

C 1.5088726177   1.8571823356   -1.6159041128

O 0.7612886177   1.1879833356   -0.5233201128

O -1.6275433823   2.0611653356   -1.1396401128

O 1.7330396177   1.8318163356   1.6526278872

O -2.8676163823   2.6990283356   1.1091298872

O -2.9100563823   0.2553713356   -2.5225191128

O 3.8934736177   0.5354663356   -2.0932701128

O 4.3002196177   1.2230933356   1.9544628872

O 4.9530986177   -0.6556216644   0.1721278872

O -0.7444973823   -1.3331916644   -2.2341271128

O 2.0311336177   -1.4618806644   -1.6224611128

O -4.6760953823   0.5659913356   1.3398548872

O -4.8942193823   -0.9721896644   -0.8517871128

O -3.5679353823   -3.1505906644   0.2086768872

O -1.4172473823   -1.5408086644   0.5093078872

O -1.9239563823   0.3570673356   2.3600578872

O 0.5455466177   -0.0267536644   3.2691348872

O 1.2777276177   -1.6522926644   1.0492418872

O 3.4497376177   -3.0855256644   0.2471278872

H 5.0311626177   1.8417963356   1.9757198872

H 4.5724506177   0.4930353356   1.3489308872

H 4.6911726177   -0.2688106644   -0.6817331128

H 4.5628086177   -1.5422056644   0.2043808872

H 0.2222796177   -1.3170696644   -2.2855561128

H -0.9387693823   -1.4055546644   -1.2869951128

H -2.8633083823   3.6157313356   1.3883958872

H -2.4291123823   2.6534033356   0.2282728872

H 2.7121406177   1.7136863356   1.7037048872

H 1.3699436177   1.3042413356   2.3859118872

H 2.5290026177   -2.2698526644   -1.4457951128

H 2.6799046177   -0.8239306644   -1.9694421128

H 3.1249986177   1.1595233356   -1.8880871128

H 4.2864076177   0.8406243356   -2.9129381128

H 2.6850926177   -2.7692966644   0.7659818872

H 3.6930406177   -3.9488886644   0.5834598872

H 1.2356976177   -1.0342116644   1.7970478872

H 1.4651926177   -1.1545926644   0.2311418872

H -0.2163913823   1.3904533356   -0.6089601128

H 1.1661076177   1.4745933356   0.4075478872

H -0.4168123823   0.1217283356   3.1018218872

H 0.6598976177   -0.2312296644   4.1982468872

H -3.5836063823   -0.2499226644   -2.0424121128

H -2.1569263823   -0.3528566644   -2.6639741128

H -4.2908913823   1.4442813356   1.1858888872

H -4.0649413823   0.1739873356   1.9766538872

H -4.8747713823   -0.3739926644   -0.0672661128

H -5.7832873823   -0.9319496644   -1.2095131128

H -2.0787203823   -2.2612286644   0.5026998872

H -0.5497543823   -1.8903646644   0.7787418872

H -4.1217173823   -3.5892386644   0.8553238872

H -4.1353013823   -2.5047446644   -0.2516451128

H -2.1694553823   1.4008633356   -1.6637141128

H -1.1011543823   2.5609873356   -1.7945091128

H -1.8108093823   -0.2519576644   1.5968968872

H -2.0848413823   1.2460353356   2.0019588872

H 1.2235046177   3.0939203356   -3.3704341128

57

w18_v3_TS2_c1

C 0.8845504427   1.7361250131   -3.0198313081

C 1.4618124427   1.1534610131   -2.0135833081

O 0.8342384427   0.9146140131   -0.7573103081

O -1.6596975573   1.6448920131   -1.6464213081

O 1.9958184427   2.2749110131   0.9777356919

O -2.8546375573   2.9332210131   0.2821906919

O -2.9550475573   -0.4850649869   -2.5028473081

O 3.6574374427   -0.2251209869   -2.0698873081

O 4.4130344427   1.7348980131   1.1813936919

O 4.8908214427 -0.6151009869 0.2555726919

O -0.8537925573 -1.9744649869 -1.6778183081

O 1.9184164427 -2.0218509869 -1.1048173081

O -4.6130345573 0.9658730131 1.1710306919

O -4.9390475573 -1.1157369869 -0.5074153081

O -3.6583175573 -2.8867699869 1.1738866919

O -1.4935505573 -1.2739099869 1.0198526919

O -1.7446115573 1.1582410131 2.1363796919

O 0.7338854427 1.1123780131 2.9464966919

O 1.2248674427 -1.3331989869 1.4645066919

O 3.4668204427 -2.8795619869 1.2191846919

H 5.0605974427 2.3046490131 0.7622716919

H 4.6243234427 0.7932500131 0.8909546919

H 4.5289404427 -0.5323939869 -0.6587973081

H 4.5282984427 -1.4344559869 0.6273966919

H 0.1129934427 -2.0199059869 -1.7308903081

H -1.0387095573 -1.7080049869 -0.7652503081

H -2.9209545573 3.8867980131 0.2184626919

H -2.4253995573 2.6004350131 -0.5464473081

H 3.0176944427 2.0711960131 1.0024546919

H 1.5900474427 1.9686430131 1.8303776919

H 2.4267074427 -2.7610739869 -0.7539933081

H 2.5660064427 -1.4613969869 -1.5911733081

H 2.7396784427 0.4927780131 -2.0804853081

H 4.0417124427 -0.2205639869 -2.9486583081

H 2.6671884427 -2.4365549869 1.5617456919

H 3.7182164427 -3.5519459869 1.8534696919

H 1.2290404427 -0.4770739869 1.9163696919

H 1.3804394427 -1.1847129869 0.5082816919

H -0.1326695573 1.0564680131 -0.8587843081

H 1.5155594427 1.7390670131 0.2222166919

H -0.2417395573 1.1895710131 2.7469796919

H 0.8519944427 1.1665810131 3.8963606919

H -3.6343825573 -0.8086519869 -1.8930833081

H -2.2121455573 -1.1178439869 -2.4361643081

H -4.1929615573 1.7512990131 0.7791216919

H -4.0450225573 0.7577510131 1.9207326919

H -4.8735885573 -0.3239939869 0.0755456919

H -5.8352805573 -1.1356549869 -0.8483273081

H -2.1732525573 -1.9489889869 1.2158926919

H -0.6276255573 -1.5760419869 1.3444926919

H -4.2342545573   -3.1367619869   1.8969066919

H -4.2129465573   -2.4180439869   0.5231396919

H -2.1916535573   0.8761700131   -1.9921033081

H -1.0616335573   1.9160370131   -2.3699393081

H -1.7445035573   0.3185340131   1.6233236919

H -1.9966555573   1.8769220131   1.5314086919

H 1.2110264427   1.9402810131   -4.0302923081

57

w18_v3_TS2_C2

C 0.8089873464   -0.2141285726   -3.4723508363

C 1.3984433464   0.0603294274   -2.3536858363

O 0.7032073464   0.4228084274   -1.1561298363

O -1.4407596536   0.3484184274   -2.1754748363

O 1.7056623464   2.5624614274   -0.1875628363

O -2.7534266536   2.5996444274   -1.4607888363

O -2.7806056536   -1.7624535726   -1.7432948363

O 3.8608343464   -1.1792695726   -1.8534148363

O 4.2974973464   2.3115834274   0.4843571637

O 4.9372453464   -0.2941365726   0.5558301637

O -0.7408846536   -2.7641135726   -0.3353568363

O 2.0117863464   -2.2567955726   -0.0545848363

O -4.6461636536   1.4417314274   0.2892051637

O -4.8580686536   -1.2250705726   0.1218921637

O -3.6166956536   -1.7624825726   2.5372021637

O -1.4202336536   -0.6479005726   1.4264731637

O -1.9515996536   1.9910644274   1.1951691637

O 0.4635633464   2.4253984274   2.2768781637

O 1.2647093464   -0.2760475726   1.8407241637

O 3.4063403464   -1.8597315726   2.3838801637

H 5.0464993464   2.7664154274   0.0979261637

H 4.5601163464   1.3653994274   0.5619801637

H 4.6701003464   -0.6597505726   -0.3044908363

H 4.5461953464   -0.8693465726   1.2306751637

H 0.2226583464   -2.7425015726   -0.4478708363

H -0.9217106536   -2.0642095726   0.3106861637

H -2.6531006536   3.3986074274   -1.9811958363

H -2.3056206536   1.8780544274   -1.9449808363

H 2.6791813464   2.5153534274   -0.0641788363

H 1.3498423464   2.7556684274   0.6939441637

H 2.5330223464   -2.5846735726   0.6894641637

H 2.6479223464   -2.0835475726   -0.7679628363

H 3.0930993464 -0.6012905726 -2.1296668363

H 4.2509543464 -1.5207625726 -2.6600888363

H 2.6466833464 -1.2505225726 2.4689631637

H 3.6178863464 -2.1878635726 3.2586111637

H 1.2157023464 0.6906664274 1.9221001637

H 1.4105333464 -0.5020875726 0.9044601637

H -0.4828716536 0.4152474274 -1.4758088363

H 1.0943683464 1.2844324274 -0.7895998363

H -0.4807496536 2.4028154274 1.9997101637

H 0.5249873464 2.9372864274 3.0843581637

H -3.4905836536 -1.6984415726 -1.0840488363

H -2.0793146536 -2.3322305726 -1.3573248363

H -4.2729406536 1.8850464274 -0.4882878363

H -4.0087336536 1.6626334274 0.9827611637

H -4.8492886536 -0.2377075726 0.1399431637

H -5.7339756536 -1.4911505726 -0.1648968363

H -2.0833586536 -1.0590145726 2.0154121637

H -0.5479486536 -0.6401465726 1.8599961637

H -4.1713166536 -1.4969895726 3.2714581637

H -4.1680306536 -1.7073645726 1.7354771637

H -2.0134116536 -0.4813315726 -1.9845048363

H -0.8407186536 0.1220804274 -2.9879308363

H -1.8077886536 1.0195974274 1.1455841637

H -2.0291926536 2.3195964274 0.2850481637

H 1.2506293464 -0.5675035726 -4.3958098363

57

W18_v3_P_C1

C 1.6114393346 1.3386870177 2.2985449270

C 1.6961783346 0.4407580177 1.3504169270

O 0.6465993346 -0.2703259823 0.8090819270

O -1.3788276654 0.6205520177 2.4056209270

O 1.5459163346 -2.9639599823 0.9377069270

O -2.5009466654 -1.7874949823 2.3048269270

O -2.7292746654 2.5229150177 1.2488279270

O 4.1904923346 2.4642180177 1.0347159270

O 4.1979763346 -2.3937509823 0.5968329270

O 4.7382973346 0.1687080177 -0.2831060730

O -0.5590066654 2.4176560177 -0.3054510730

O 2.1230173346 2.7673080177 -0.9118290730

O -4.6107346654 -1.2463649823 0.5095439270

O -4.8933766654 1.3281260177 -0.2060800730

```
O -3.7782426654   1.1682640177   -2.7260790730
O -1.5445216654   0.0982590177   -1.6518250730
O -2.0466256654   -2.2705879823   -0.4262650730
O 0.1031633346   -3.5109139823   -1.4255680730
O 1.0045073346   -0.8952949823   -1.8992030730
O 3.1443393346   0.6201390177   -2.5650370730
H 4.6848703346   -2.7851939823   1.3228419270
H 4.6250333346   -0.7546039823   0.0079569270
H 4.5030103346   1.6193060177   0.6465129270
H 4.3348653346   0.2378580177   -1.1616050730
H 0.3798383346   2.6171150177   -0.4854020730
H -0.7835536654   1.6297080177   -0.8186780730
H -2.3584776654   -2.3793679823   3.0451779270
H -2.0789076654   -0.9279979823   2.5276979270
H 3.2601953346   -2.6423789823   0.7317549270
H 1.1314513346   -3.3607409823   0.1561589270
H 2.5047283346   2.1615820177   -1.5621110730
H 2.7578563346   2.7822290177   -0.1763350730
H 2.6513363346   0.1735520177   0.8948399270
H 4.9485483346   3.0500510177   1.0352579270
H 2.3954913346   -0.0051289823   -2.4726420730
H 3.3644023346   0.6678700177   -3.4963960730
H 0.8934713346   -1.8609329823   -1.9162510730
H 0.8950213346   -0.6471639823   -0.9617720730
H -0.1893266654   0.0837040177   1.1857869270
H 1.0925533346   -2.1157999823   1.0506279270
H -0.7755966654   -3.1993699823   -1.1036020730
H -0.0365196654   -4.3107229823   -1.9342820730
H -3.4741776654   2.2341690177   0.7004899270
H -1.9981976654   2.7477730177   0.6372579270
H -4.1220426654   -1.4015089823   1.3332049270
H -4.0872186654   -1.7349039823   -0.1398980730
H -4.8390306654   0.3898960177   0.0954119270
H -5.7649546654   1.6464330177   0.0368469270
H -2.2655706654   0.4507220177   -2.2118530730
H -0.7810586654   -0.1386059823   -2.1990370730
H -4.4056366654   0.8155530177   -3.3578410730
H -4.2743526654   1.3367920177   -1.9034540730
H -1.9500016654   1.3451760177   2.0124839270
H -0.7738306654   1.0510070177   3.0193139270
H -1.8989696654   -1.3689439823   -0.7868210730
```

H -2.0197786654   -2.1934709823   0.5426819270

H 2.2985413346   2.0355140177   2.7526679270

57

w18_v3_P_c2

C 0.8546783857   2.8178732537   1.7702528811

C 1.4582063857   1.7185392537   1.3665968811

O 1.0897323857   0.4738412537   1.1954048811

O -2.2439796143   2.4030732537   1.1154018811

O 2.5156013857   -1.2622167463   2.3629228811

O -2.3754046143   0.3588692537   2.7578318811

O -3.8981046143   2.2715682537   -1.1071001189

O 3.7334623857   2.4436422537   -0.2984731189

O 4.8593063857   -1.5422117463   1.0588868811

O 4.9625923857   0.0350652537   -1.1204011189

O -1.0796916143   1.6569232537   -1.2638661189

O 1.6316243857   1.4521612537   -1.9360651189

O -4.3000746143   -1.2229267463   1.4778368811

O -5.1287946143   -0.3266067463   -0.9049851189

O -3.6304816143   -1.7523197463   -2.7102791189

O -1.4300566143   -1.1069127463   -1.2832421189

O -1.4990136143   -1.8582607463   1.3262058811

O 0.8383163857   -3.1007887463   1.1585148811

O 1.3013753857   -1.4093357463   -1.0476361189

O 3.0251663857   -0.7638567463   -3.0038691189

H 5.7413083857   -1.5909297463   1.4284478811

H 4.9267563857   -0.9971557463   0.2426768811

H 4.6500703857   0.9233672537   -0.8921441189

H 4.4219063857   -0.2518887463   -1.8734291189

H -0.1616226143   1.7644692537   -1.5629821189

H -1.2253916143   0.6946252537   -1.2671921189

H -2.1741346143   0.6385642537   3.6519748811

H -2.3064546143   1.1652182537   2.1778208811

H 3.4114673857   -1.3434847463   1.9703098811

H 2.0516783857   -2.0828827463   2.1294468811

H 2.0598363857   0.8986242537   -2.6031871189

H 2.3342953857   1.9911872537   -1.5480721189

H 3.1427083857   2.2128582537   0.4485368811

H 4.1068603857   3.3048722537   -0.0999091189

H 2.4358523857   -1.2224147463   -2.3682641189

H 3.0942613857   -1.3090127463   -3.7885881189

H 1.3181873857   -2.0682957463   -0.3302921189

H 1.2893303857 -0.5400157463 -0.6121501189

H -1.5939056143 2.2191882537 0.4094578811

H 1.6856533857 -0.1729767463 1.7206458811

H -0.0638996143 -2.7265547463 1.3248738811

H 0.7656403857 -4.0554187463 1.1994248811

H -4.2995936143 1.3920962537 -1.1605191189

H -3.0351526143 2.1934962537 -1.5373291189

H -3.9051646143 -0.5546737463 2.0625918811

H -3.5699076143 -1.8490847463 1.3727288811

H -4.8550416143 -0.6379547463 -0.0066811189

H -6.0824466143 -0.2270767463 -0.8789951189

H -2.1022526143 -1.4454727463 -1.9084681189

H -0.5417016143 -1.3971117463 -1.5443481189

H -4.0091766143 -2.6176647463 -2.8687731189

H -4.2745596143 -1.2652157463 -2.1603571189

H -3.0588376143 2.5117452537 0.5934148811

H -0.1670166143 2.7957502537 2.1453268811

H -1.5264676143 -1.5131987463 0.4088848811

H -1.5588726143 -1.0753837463 1.9045658811

H 1.3632363857 3.7733992537 1.7258508811

59

w18_H1_r

C 1.7084326293 -1.6216658444 -2.4210841844

C 1.9019586293 -0.5355158444 -1.7019561844

O 0.9234946293 0.2741311556 -1.2142441844

O -1.4804643707 -0.2760118444 -2.5845161844

O 1.7218656293 3.0085081556 -0.9960811844

O -2.9403303707 1.9128361556 -2.0329851844

O -2.6933933707 -2.3483498444 -1.5640261844

O 4.4183026293 -2.3562358444 -0.6743851844

O 4.2987386293 2.5363361556 -0.2020301844

O 4.8698936293 -0.0531008444 0.6664028156

O -0.5334683707 -2.1758328444 -0.0148821844

O 2.0521506293 -2.6132748444 0.9123968156

O -4.9297403707 1.1062161556 -0.2035131844

O -4.8556423707 -1.5588788444 0.2159708156

O -3.6038593707 -1.5839728444 2.7277588156

O -1.6112183707 -0.0896368444 1.6611568156

O -2.3393433707 2.3543751556 0.6985688156

O 0.0401716293 3.4474621556 1.2693888156

O 0.9992296293 0.8205701556 1.6387208156

O 3.0231486293 -0.5817438444 2.7802328156

H 4.8937716293 3.0320161556 -0.7526271844

H 4.7146096293 0.8502531556 0.3653958156

H 4.6810026293 -1.5121318444 -0.2705131844

H 4.3783506293 -0.1556908444 1.4842008156

H 0.3610846293 -2.4086828444 0.2733728156

H -0.8578773707 -1.5126948444 0.5966058156

H -2.9346793707 2.5696651556 -2.7210201844

H -2.4049003707 1.1601601556 -2.3446781844

H 3.4020686293 2.7606641556 -0.4963351844

H 1.1514736293 3.3266951556 -0.2917661844

H 2.4019746293 -2.1212928444 1.6558758156

H 2.7698696293 -2.6389008444 0.2696788156

H 2.8819246293 -0.1936748444 -1.4131811844

H 5.1633236293 -2.9378418444 -0.5753081844

H 2.3013496293 0.0116481556 2.5221558156

H 3.1081686293 -0.5351898444 3.7258138156

H 0.8549746293 1.7735841556 1.6232978156

H 1.0425236293 0.5515691556 0.7131978156

H 0.0612126293 -0.0361328444 -1.5306501844

H 1.3669556293 2.1509601556 -1.2414311844

H -0.8855273707 3.1839231556 1.0969308156

H 0.0204866293 4.2189961556 1.8250218156

H -3.4418533707 -2.1851508444 -0.9844201844

H -1.9504613707 -2.5795388444 -0.9849031844

H -4.4364143707 1.3656421556 -0.9882031844

H -4.5199933707 1.6144791556 0.4950828156

H -4.9311703707 -0.5994088444 0.0522498156

H -5.7069933707 -1.9341368444 0.0150798156

H -2.1873573707 -0.5718298444 2.2689968156

H -0.7594553707 0.0936441556 2.0655618156

H -4.2287453707 -1.5547978444 3.4425458156

H -4.1096813707 -1.7030298444 1.9145528156

H -1.9862533707 -1.0541658444 -2.2351711844

H -1.1915693707 -0.5155138444 -3.4602011844

H -2.1525543707 1.4536571556 1.0119958156

H -2.4279963707 2.2909891556 -0.2576391844

H 2.5631026293 -2.1971538444 -2.7254691844

H 1.1651146293 -4.7139718444 -1.3142621844

H 0.7204596293 -1.9850608444 -2.6505181844

59

w18_H1_ts

C 1.8650233108 -1.4158911104 -2.5805142171

C 1.8916713108 -0.5310411104 -1.5910152171

O 0.8341053108 0.1918998896 -1.1470462171

O -1.4750836892 -0.3503891104 -2.6267152171

O 1.6989133108 2.9383708896 -0.9889442171

O -2.8883696892 1.8793028896 -2.0825802171

O -2.6310636892 -2.3405331104 -1.4767922171

O 4.4064013108 -2.3757951104 -0.7288312171

O 4.3247473108 2.4991268896 -0.3252442171

O 4.8792933108 -0.0725871104 0.5992617829

O -0.6100966892 -2.3721291104 0.2479157829

O 2.0848573108 -2.5553861104 0.9212687829

O -4.9201636892 1.1507478896 -0.2617082171

O -4.8708946892 -1.5127391104 0.1923107829

O -3.7410786892 -1.4651471104 2.7591447829

O -1.6516306892 -0.0926741104 1.7162817829

O -2.3093006892 2.3240398896 0.6550377829

O 0.0441133108 3.4383798896 1.2747047829

O 0.9635453108 0.8019238896 1.6955917829

O 3.0840413108 -0.5231021104 2.7624127829

H 4.8793173108 2.9442928896 -0.9555322171

H 4.7448263108 0.8284508896 0.2810787829

H 4.7092183108 -1.5488311104 -0.3186692171

H 4.4098853108 -0.1420841104 1.4336267829

H 0.3297403108 -2.5224881104 0.4178167829

H -0.8723706892 -1.6183821104 0.7771677829

H -2.8398976892 2.5415438896 -2.7637822171

H -2.3770096892 1.1087318896 -2.3889732171

H 3.4104963108 2.7052278896 -0.5736152171

H 1.1573223108 3.2858538896 -0.2755992171

H 2.4683263108 -2.0764441104 1.6560977829

H 2.7906123108 -2.6391341104 0.2709307829

H 2.7835673108 -0.3253191104 -1.0241632171

H 5.1359093108 -2.9828291104 -0.6819182171

H 2.3320303108 0.0373298896 2.5164057829

H 3.1800373108 -0.4745891104 3.7068317829

H 0.8283583108 1.7557728896 1.6657217829

H 0.9357183108 0.5101158896 0.7771127829

H 0.0231173108 -0.0713861104 -1.6119012171

H 1.3193473108 2.0830628896 -1.1989962171

H -0.8766596892   3.1705558896   1.0832687829

H 0.0107433108   4.2154298896   1.8218747829

H -3.3890866892   -2.1599701104   -0.9150662171

H -1.9037696892   -2.5865361104   -0.8787242171

H -4.4205626892   1.3922608896   -1.0476612171

H -4.4931796892   1.6496788896   0.4335917829

H -4.9448396892   -0.5569221104   0.0100397829

H -5.7075896892   -1.8994031104   -0.0450322171

H -2.2722836892   -0.5348971104   2.3112297829

H -0.8223396892   0.0911328896   2.1625617829

H -4.3989296892   -1.3681601104   3.4374247829

H -4.2101766892   -1.6153441104   1.9293507829

H -1.9671386892   -1.1128221104   -2.2087542171

H -1.2867636892   -0.6177981104   -3.5212442171

H -2.1378906892   1.4291608896   0.9946907829

H -2.3782546892   2.2414058896   -0.3011842171

H 2.7840323108   -1.8965301104   -2.8597892171

H 1.4063813108   -3.2703381104   -1.8039552171

H 0.9844633108   -1.5843031104   -3.1763042171

59

w18_H1_p

C 1.4406894892   1.4935407809   2.5790879788

C 1.7562209776   0.5261796542   1.5189914883

O 0.7907260453   -0.3626776504   1.1243112027

O -1.6333433503   0.1664640289   2.4526185160

O 1.9971652003   -2.9202327754   0.9016449650

O -2.7949609196   -2.2036633412   1.9808972153

O -3.0182655292   2.0681316897   1.3266676438

O 4.0345698262   2.8114829459   1.0615693492

O 4.5067414205   -1.9456792659   0.3778715482

O 4.8989115562   0.7120124701   -0.4119226650

O -0.7013637137   2.2545913305   0.0203893283

O 1.8238078954   3.1157052934   -0.7100290218

O -4.7366124962   -1.6533365315   0.0064046680

O -5.0116383294   0.9829717181   -0.4851371509

O -3.6069542461   1.1462073447   -2.8878927583

O -1.4800738877   0.0149635574   -1.6284899648

O -1.9796754683   -2.5110372743   -0.7159393300

O 0.4262329226   -3.4897298576   -1.4149153225

O 1.1555375608   -0.7734338939   -1.6761654026

O 2.9694918298   1.0850469350   -2.4894819351

H 5.1242106816 -2.4068711260 0.9336866430

H 4.8106176015 -0.2081241783 -0.1373182869

H 4.4126871768 2.0335105525 0.6152346027

H 4.3775744070 0.7981912393 -1.2134469897

H 0.1591990343 2.6308274226 -0.2207185647

H -0.8698032761 1.5295904555 -0.5840113122

H -2.7485248201 -2.8356085683 2.6901790659

H -2.3759339015 -1.3813234339 2.2934602533

H 3.6369698356 -2.3364333727 0.5548562168

H 1.5220416245 -3.3013437614 0.1590479541

H 2.1940758304 2.5564527821 -1.3952412389

H 2.4925821223 3.1117960992 -0.0155768190

H 2.7530865722 0.1711374650 1.3226140190

H 4.7279372361 3.4614136282 1.0813026631

H 2.3566210719 0.3510261326 -2.3302139064

H 3.1075825688 1.1374187097 -3.4287712002

H 1.1294374141 -1.7319707934 -1.7618921940

H 1.0731723635 -0.6039603167 -0.7287112864

H -0.0774630789 -0.0714220889 1.4414262932

H 1.4755818426 -2.1587302726 1.1655944848

H -0.4988560875 -3.2670166072 -1.1944980981

H 0.4100856475 -4.2641821594 -1.9663922151

H -3.6945712089 1.8122233702 0.6947001337

H -2.2754037906 2.4223010319 0.8143677793

H -4.2898981936 -1.8412680854 0.8376168593

H -4.1804718097 -2.0852123901 -0.6416235979

H -4.9686587194 0.0273296689 -0.2885730770

H -5.9061930198 1.2568834637 -0.3106270051

H -2.1061079878 0.3917117297 -2.2621920931

H -0.6297680980 -0.1384540768 -2.0493440985

H -4.1453625921 0.9593151191 -3.6478926275

H -4.1995583131 1.1980995590 -2.1276326808

H -2.2009204150 0.8684132869 2.0416808465

H -1.3549129318 0.5169887415 3.2935367296

H -1.8660464704 -1.5850765406 -0.9891206966

H -2.1029908558 -2.4968042662 0.2383866477

H 2.2782707280 2.1698664398 2.7030021247

H 0.5614624572 2.0795852663 2.3152374971

H 1.2491139322 1.0071493896 3.5439512957

59

w18_H2_r

C 1.7375339559 -1.4609865106 -2.4179322826

C 1.8805519559 -0.4082225106 -1.6400832826

O 0.8657209559 0.3213864894 -1.1001222826

O -1.4908310441 -0.2910495106 -2.5190272826

O 1.6353219559 3.0645024894 -0.8853702826

O -2.9444230441 1.9080844894 -2.0371562826

O -2.6831950441 -2.3846835106 -1.5271862826

O 4.4566729559 -2.3444005106 -0.7734362826

O 4.2396689559 2.5023954894 -0.2504382826

O 4.8915949559 -0.0681775106 0.6261717174

O -0.4681680441 -2.2783885106 -0.0470902826

O 2.1271729559 -2.7498425106 0.8371147174

O -4.9158360441 1.0782924894 -0.1976452826

O -4.8673320441 -1.5870915106 0.2218537174

O -3.5958470441 -1.6170645106 2.7110137174

O -1.5692220441 -0.1909445106 1.6079627174

O -2.3133230441 2.2686214894 0.6921717174

O -0.0164480441 3.4504044894 1.4069637174

O 0.9895159559 0.8343384894 1.7231057174

O 3.0283509559 -0.6719065106 2.7106727174

H 4.8112309559 3.0079694894 -0.8166632826

H 4.7092119559 0.8316684894 0.3310467174

H 4.7013269559 -1.5040385106 -0.3509852826

H 4.3883499559 -0.1963335106 1.4332027174

H 0.4303679559 -2.5241035106 0.2182907174

H -0.7691930441 -1.6141205106 0.5751017174

H -2.9232130441 2.5612224894 -2.7283002826

H -2.4133580441 1.1472034894 -2.3368632826

H 3.3323669559 2.7594714894 -0.4768112826

H 1.0948209559 3.3787004894 -0.1559592826

H 2.4474469559 -2.2185795106 1.5670207174

H 2.8365729559 -2.7247615106 0.1852417174

H 2.8442149559 -0.0275095106 -1.3433242826

H 5.2190729559 -2.9065545106 -0.6960372826

H 2.3078499559 -0.0560605106 2.5086277174

H 3.1370699559 -0.6819745106 3.6550437174

H 0.8392659559 1.7849964894 1.7666807174

H 1.0048009559 0.6211704894 0.7815657174

H 0.0147149559 -0.0172765106 -1.4189402826

H 1.2579189559 2.2172754894 -1.1317232826

H -0.9193810441 3.1491244894 1.1844597174

H -0.0980490441 4.2173914894 1.9631337174

H -3.4187970441 -2.2245075106 -0.9308342826

H -1.9297810441 -2.6398715106 -0.9724172826

H -4.4443840441 1.3460464894 -0.9926662826

H -4.4737150441 1.5673284894 0.4953727174

H -4.9372650441 -0.6286065106 0.0497807174

H -5.7139310441 -1.9638295106 0.0046617174

H -2.1680380441 -0.6558125106 2.2083537174

H -0.7472730441 0.0305184894 2.0535717174

H -4.2038550441 -1.5421345106 3.4369987174

H -4.1232060441 -1.7318425106 1.9107807174

H -1.9910230441 -1.0766175106 -2.1770402826

H -1.1650410441 -0.5351545106 -3.3803992826

H -2.1168690441 1.3586404894 0.9713577174

H -2.4016370441 2.2441344894 -0.2661012826

H 2.6180909559 -1.9726095106 -2.7598792826

H 0.7688529559 -1.8678005106 -2.6553472826

H 0.8366089559 1.6072634894 -4.1407362826

59

w18_H2_ts

C 3.1541641219 -0.4662377826 -0.1053998465

C 2.3325799991 -0.0047207019 0.8438069267

O 1.0604524512 0.4157571034 0.6147692275

O 1.3685675474 1.8040017891 -1.7902335365

O 0.3961283489 1.9934946745 2.9369438569

O -0.3978682850 3.7006590398 -1.1448406187

O 0.6423604768 0.4023452032 -3.8827942477

O 3.6902719395 -3.2220532975 1.4356536861

O 1.7728673629 0.1570058355 4.4246882234

O 2.1482969835 -2.4751437678 3.5400865416

O 0.4948161444 -1.3479369664 -1.8472838820

O 1.2841060527 -3.3389124512 -0.0861009778

O -2.5968157184 3.1190488936 -2.8265226230

O -2.1219161690 1.0678100637 -4.5111388884

O -3.4771136553 -0.9627924330 -3.1456441053

O -2.0416016959 -0.4229151966 -0.9138535311

O -2.4493498369 2.1307304564 -0.0314399195

O -2.3814804382 1.4920315717 2.5647352256

O -1.2235735047 -0.9246848614 1.6810770096

O -0.3116704311 -3.3776267319 2.3833266158

H 2.3650361547 0.6034618242 5.0187508237

H 2.0135810015 -1.5902274805 3.8963497160

H 3.2427259509 -2.9064523851 2.2383197469

H 1.2805890514 -2.8157574320 3.3120224534

H 0.8064461986 -2.0873276277 -1.3037363870

H -0.3817490318 -1.1253287936 -1.5259372052

H -0.0199099998 4.4894745650 -0.7708541560

H 0.3398346514 3.1050858885 -1.3737110293

H 1.2630201104 0.8465901610 3.9716022488

H -0.5631961996 1.9436722138 2.9564025035

H 0.7173024541 -3.5074833771 0.6677489032

H 2.1742746644 -3.2996524892 0.2823197016

H 2.5332440705 -0.1314449048 1.8949186684

H 4.1501754579 -4.0138044761 1.6910285568

H -0.7938643854 -2.5502425509 2.2335234041

H -0.9363459588 -4.0177722492 2.7055113427

H -1.6602087883 -0.2315157201 2.1879005282

H -0.4001735782 -0.5321785433 1.3642340183

H 0.9710151665 0.6307973324 -0.3250862176

H 0.6425787062 1.7530678725 2.0423159257

H -2.5335459438 1.8218701009 1.6571988403

H -3.1299011522 1.7444245371 3.0941799709

H -0.2592362720 0.5551345139 -4.1762208340

H 0.6284086933 -0.4256121706 -3.3812867127

H -1.8035776802 3.5184767607 -2.4570206643

H -3.0781359584 2.8347179964 -2.0496333856

H -2.2954131164 1.8468179174 -3.9480498012

H -2.2752758521 1.3440752573 -5.4089548581

H -2.6834219508 -0.7697142483 -1.5489202861

H -2.0838651796 -0.9068035353 -0.0852905124

H -4.3824137891 -1.0513813271 -3.4192289209

H -3.0608050518 -0.3292298913 -3.7435727626

H 1.1316327309 1.3402970348 -2.6298232451

H 2.3176855709 1.8863408794 -1.7679530823

H -2.2835474275 1.2550458167 -0.4180096704

H -1.6869224348 2.6746546994 -0.2586929197

H 4.1122821468 -0.8586306353 0.1803882089

H 2.8632875660 -0.4739749403 -1.1422796457

H 3.1478627057 1.6961108026 1.0417139108

59

w18_H2_p

C 1.5982715808 -1.3310195961 -2.4063023117

C 1.8998305808 -0.1243885961 -1.6248563117

O 0.7698715808 0.4180454039 -0.9475513117

O -1.4464804192 -0.4497205961 -2.4783193117

O 1.3355245808 3.1406614039 -0.8853733117

O -2.6814264192 1.8753944039 -2.0867593117

O -2.6748774192 -2.4964425961 -1.4083653117

O 4.4016635808 -2.4264005961 -0.8327183117

O 3.9511115808 2.4132924039 -0.5838963117

O 4.7662945808 -0.0553225961 0.4356296883

O -0.4154704192 -2.2840105961 0.0048696883

O 2.1518805808 -2.8110875961 0.8651846883

O -4.7180254192 1.0986594039 -0.2929243117

O -4.8070304192 -1.5310715961 0.2471636883

O -3.5995864192 -1.5129305961 2.7365256883

O -1.5066984192 -0.2281825961 1.6245786883

O -2.1923704192 2.2092524039 0.6443246883

O -0.0263904192 3.4695584039 1.5676266883

O 0.9941915808 0.8710354039 1.8042616883

O 3.0544125808 -0.6784355961 2.6045896883

H 4.5327785808 3.0670504039 -0.9534823117

H 4.5239925808 0.8141144039 0.0967146883

H 4.6468045808 -1.5626205961 -0.4617563117

H 4.3139495808 -0.1636705961 1.2750146883

H 0.4588975808 -2.5554105961 0.3198606883

H -0.7214634192 -1.5908045961 0.5941706883

H -2.5813984192 2.5233224039 -2.7758803117

H -2.2198124192 1.0667364039 -2.3770963117

H 3.0469475808 2.7498494039 -0.6907583117

H 0.9277145808 3.4748434039 -0.0832093117

H 2.4708535808 -2.2243765961 1.5534256883

H 2.8644795808 -2.8362375961 0.2164166883

H 2.6462195808 -0.3543965961 -0.8626283117

H 5.1910725808 -2.9551515961 -0.8225753117

H 2.3268385808 -0.0504195961 2.4640976883

H 3.2242705808 -0.7111645961 3.5395556883

H 0.8492805808 1.8200664039 1.8794216883

H 0.9529175808 0.6768724039 0.8541256883

H -0.0333334192 0.0167454039 -1.3032543117

H 0.9542735808 2.2648734039 -1.0113933117

H -0.9029594192 3.1398864039 1.2867826883

H -0.1675194192 4.2132284039 2.1430106883

H -3.4063224192 -2.2862205961 -0.8227503117

H -1.9228584192 -2.7242125961 -0.8426013117

H -4.2607644192 1.3375874039 -1.1043263117

H -4.2310014192 1.5816344039 0.3757886883

H -4.8174414192 -0.5796825961 0.0216316883

H -5.6683944192 -1.8710075961 0.0270806883

H -2.1460844192 -0.6528075961 2.2145186883

H -0.7138284192 0.0202504039 2.1075826883

H -4.2111614192 -1.3387405961 3.4423166883

H -4.1217964192 -1.6335755961 1.9328526883

H -1.9591384192 -1.2146895961 -2.1188743117

H -0.9275814192 -0.7881655961 -3.2030733117

H -1.9879034192 1.3010914039 0.9262106883

H -2.2076694192 2.1974174039 -0.3193813117

H 2.3261685808 -1.6631795961 -3.1265743117

H 0.8876955808 -2.0419415961 -2.0176273117

H 2.3382745808 0.6487924039 -2.2557783117

58

VinylAlc_w18

C -1.2985172052 3.6907467743 -0.7092003519

C -2.4926562052 3.1424157743 -0.8727743519

O -2.7705092052 2.2545947743 -1.8376933519

O -4.9822392052 1.1529487743 -1.0533553519

O -4.6148052052 -0.8749412257 0.6061536481

O -0.7614242052 0.6947637743 -2.7709873519

O -0.8874722052 -1.1882842257 -0.7320033519

O -2.8633822052 -2.9575812257 -0.1350713519

O -1.5486652052 -2.7947722257 2.4331586481

O -2.7589852052 -0.1629992257 2.6410416481

O -0.8452322052 1.0411187743 1.1161526481

O 1.5855887948 1.6450587743 2.2033306481

O 2.2692447948 -0.5776542257 1.0367796481

O 1.1745017948 -2.8636282257 1.7315566481

O 1.1419887948 -3.2199222257 -1.0277173519

O 3.3313697948 -1.6889302257 -2.0355083519

O 4.9322067948 -0.8774582257 0.2392286481

O 5.5099427948 1.7419327743 0.0872696481

O 3.0239587948 2.7945037743 -0.0551483519

O 1.7756157948 0.6963037743 -1.3935803519

H -5.8670982052 1.1396407743 -1.3982813519

H -4.8806842052 0.3796067743 -0.4584613519

H -4.1080122052   -0.6261202257   1.3850886481

H -4.1960322052   -1.6792892257   0.2836836481

H 0.2624437948   -2.8818782257   2.0477716481

H 1.1663227948   -3.2525122257   0.8473666481

H -0.4720112052   3.4634777743   -1.3628883519

H 2.5618627948   2.6869867743   0.7782136481

H -3.6403692052   1.8342507743   -1.6616443519

H -1.4866522052   1.3080757743   -2.5753083519

H -2.0694082052   -3.0806932257   1.6803646481

H -1.9408272052   -1.9590572257   2.6911556481

H -2.0873832052   0.3566587743   2.1699096481

H -2.9561452052   0.2986927743   3.4490316481

H -2.1436602052   -2.3921092257   -0.4603293519

H -2.8722032052   -3.7394962257   -0.6769263519

H -0.9406382052   -0.6411972257   -1.5234263519

H -0.7916412052   -0.5670922257   -0.0013063519

H -0.0216022052   1.2936047743   1.5652356481

H -1.0918692052   1.8076457743   0.5914386481

H 0.9159587948   0.7298257743   -1.8182953519

H -0.7189202052   0.5908127743   -3.7159713519

H 3.1952507948   -0.7813412257   0.8521946481

H 1.8458627948   -1.4114412257   1.3486066481

H 4.6512647948   2.2027087743   0.0453576481

H 6.0768847948   2.1694287743   -0.5444863519

H 5.2484147948   0.0420487743   0.1627576481

H 5.5865247948   -1.3562052257   0.7365866481

H 1.9412707948   -2.8414162257   -1.4132173519

H 0.5022057948   -2.5060212257   -1.0397293519

H 2.9182427948   -0.8261582257   -2.1012823519

H 4.0167447948   -1.5667542257   -1.3757303519

H 1.9590137948   0.7851857743   1.9051226481

H 1.7872197948   1.7393247743   3.1281396481

H 1.6964857948   0.1587347743   -0.5936263519

H 2.5652107948   2.1960567743   -0.6584243519

H -3.3264032052   3.3795757743   -0.2265673519

H -1.1525312052   4.4223427743   0.0648846481

58

TS_VA_to_AC_w18

C 0.2321649825   3.3108052480   1.6041575118

C -0.9850573938   3.1064182365   2.1489930154

O -1.9506888255   2.4063361236   1.6566710350

O -3.7223061115 1.6128931220 3.2669829264

O -3.0182536355 -0.8307324626 3.2234514757

O -1.8265932354 1.5667948772 -0.7270646721

O -2.3175030916 -1.1053203733 -0.8782114044

O -3.8152920845 -2.2073191897 1.0246295107

O -0.9963020910 -2.8068181728 1.0205670056

O -0.6439807365 -1.0084007223 3.2582053620

O 0.9130243717 0.6949443299 2.6879258889

O 2.9373760295 0.0281037476 1.1101341957

O 2.1497872944 -1.1913516683 -1.0695914996

O 0.7154526421 -3.4064593168 -1.1917778336

O -0.8945121082 -2.3470756453 -3.1469708096

O 0.7378334127 -0.2967797910 -4.1594494844

O 3.5249374862 -0.8102081391 -3.4648581193

O 4.7492015745 1.4967854784 -2.8727889803

O 3.1343467933 2.2748646904 -0.8627812057

O 0.7402147039 1.1466410810 -1.6757280325

H -4.5735732304 2.0165788270 3.1493068251

H -3.3826930199 0.0986879965 3.2615706867

H -1.8007690543 -0.8726495200 3.2336853541

H -3.4084911836 -1.2942663110 2.4635637306

H 0.1515098163 -3.4635348904 -0.4192974159

H 0.1268984983 -3.1829636623 -1.9319063976

H 0.4664013755 2.9752363251 0.6053424941

H 3.2283561064 1.7641354476 -0.0591531285

H -3.0781861203 2.0191493809 2.6120860722

H -1.8705747725 1.9242113204 0.2172184223

H -1.9044520749 -3.0592219942 1.1898367368

H -0.7015913074 -2.3439381778 1.8076786129

H 0.0948835049 -0.1276850778 2.9422937250

H -0.3936595460 -1.3227048336 4.1230391449

H -3.4719366765 -1.7618075983 0.2248876335

H -4.6644880556 -2.5795803886 0.8129074023

H -2.2037365315 -0.1398018886 -0.8472153749

H -1.5664819286 -1.4822913335 -0.4127336398

H 1.6547897545 0.4207435469 2.1077093811

H 0.6056922055 1.5956106860 2.4104179088

H -0.1425896364 1.3197666572 -1.3203621051

H -2.3027818289 2.1824082117 -1.2749603731

H 2.6876849510 -1.1592360346 -1.8725027311

H 1.6698691046 -2.0493555197 -1.0832597222

H 4.2283413184 1.8458438441 -2.1253303978

H 4.9105892209 2.2303899944 -3.4552132766

H 4.0239638218 0.0215549947 -3.3541527863

H 4.1268095401 -1.4462986045 -3.8363639023

H -0.3702272871 -1.6718335524 -3.6000706324

H -1.5724669776 -1.8714942092 -2.6672517022

H 0.6692173687 0.3931823407 -3.4944112990

H 1.6686120742 -0.5273597471 -4.1581894420

H 2.7099469630 -0.5076007856 0.3163258514

H 3.7296150224 -0.3445133002 1.4830677526

H 1.0159129158 0.2719418027 -1.3755128556

H 2.2399836239 2.0727391500 -1.1693507836

H -1.1738430062 3.5433351569 3.1324623816

H 0.9299755452 3.9664822291 2.0956859527

58

Acetaldehyde_w18

C 0.1981711456 1.8261914999 3.0698768569

C -1.2603616614 1.7784457686 2.8561289465

O -1.8643344529 2.5007688843 2.0978307690

O -4.4621958216 1.9339658019 1.1427403589

O -4.7919896291 -0.6397218936 0.1017013657

O -0.5549601409 3.0750936263 -0.2390734238

O -1.2239017835 0.4976670664 -1.1849173957

O -3.3660694054 -0.7233682337 -2.3292693764

O -2.1370706543 -3.1383833026 -1.3224457279

O -3.1113035802 -2.2947198313 1.2784661904

O -0.9792274910 -0.5313814705 1.3677858753

O 1.6111087106 -1.3583428173 2.1538349310

O 2.0073410879 -1.3371861034 -0.4199772002

O 0.5263891171 -2.7300875883 -2.0661557424

O 0.4391428931 -0.2939118421 -3.3988751868

O 2.9534378029 0.9393077874 -2.8318545734

O 4.5975391095 -1.0079804289 -1.4428686499

O 5.7033859867 0.1265148587 0.7328625487

O 3.4360877046 0.9134656229 1.9872689151

O 1.8082156397 1.4171179774 -0.2023464590

H -5.1188820764 2.5015856445 1.5293775451

H -4.7370721259 0.2636053587 0.4323701199

H -3.8066041544 -1.7095760221 0.9019080374

H -4.5273428035 -0.6081726342 -0.8223614346

H -0.3747080339 -2.9605733350 -1.8035961076

H 0.4444154120 -2.0361660173 -2.7337933566

H 0.6868473542 2.5139495421 2.3915784167

H 2.9420421197 0.1555211930 2.3056486687

H -3.6158265422 2.1797578179 1.5282400951

H -1.0299281038 3.1341179676 0.5993573308

H -2.6523272922 -2.4757700530 -1.7879466668

H -2.3986686161 -3.0444987923 -0.4045950966

H -1.6631210720 -1.2093899947 1.4503679801

H -3.5379493351 -2.8699666313 1.9037587938

H -2.6018177294 -0.2039886950 -2.0285790461

H -3.4705295891 -0.5514441574 -3.2590418119

H -1.1834116546 1.4395015656 -1.0031589263

H -1.1017171093 0.0645722529 -0.3238233522

H -0.1519290160 -0.9306234767 1.6569249617

H 0.6102799794 0.8283850458 2.9541433366

H 0.9952760454 1.9254046005 -0.2370628665

H -0.5603762916 3.9377526991 -0.6403397691

H 2.8919129606 -1.3460570096 -0.8064759359

H 1.4379241444 -1.9073748755 -0.9911465685

H 4.9434369861 0.4344839050 1.2595322713

H 6.3476811798 0.8249006953 0.7525407844

H 5.1009946546 -0.6050361515 -0.7121353437

H 5.1218392330 -1.7266940688 -1.7794270113

H 1.3129710026 0.1122501961 -3.3571047656

H -0.0472717049 0.0751116756 -2.6587589286

H 2.7095864538 1.3675255502 -2.0093908175

H 3.6136289513 0.2949435514 -2.5694788223

H 1.8451395763 -1.4863723485 1.2063900385

H 1.7895842344 -2.1809667791 2.5975121500

H 1.5954311607 0.4861473072 -0.3584310709

H 2.8906126426 1.2563760673 1.2668081840

H -1.8187460397 1.0558334321 3.4598567930

H 0.3852955906 2.1299343011 4.0989468968

57

I2_W18_R

C 0.8539395768 3.4911831480 0.1204894540

C 1.5008555768 2.4156111480 0.4954984540

O 1.1521735768 1.2465261480 0.9391164540

O -2.2463764232 2.4465841480 -0.6929485460

O 2.1251465768 0.4533331480 3.1087004540

O -3.0039244232 1.5547851480 1.6505494540

O -3.4950704232 1.1427761480 -2.8056815460

O 3.9018545768 1.9852861480 -1.2482295460

O 4.6246815768 -0.4119918520 2.5684294540

O 5.1120735768 -0.3295348520 -0.0868505460

O -0.7581684232 0.5743771480 -2.0858235460

O 1.9954445768 0.2109531480 -2.5547035460

O -4.7066684232 -0.5798558520 1.0596324540

O -4.9508884232 -1.0883048520 -1.5720135460

O -3.1791824232 -3.1680728520 -1.9796145460

O -1.3223274232 -1.7541468520 -0.5965575460

O -1.9136394232 -1.0018708520 1.9718994540

O 0.4224285768 -1.7729728520 2.9900944540

O 1.2936665768 -1.6042758520 0.3119294540

O 3.3215165768 -2.0973848520 -1.4048315460

H 5.4311865768 -0.2295458520 3.0362534540

H 4.8403965768 -0.3927178520 1.6185344540

H 4.8501355768 0.4939471480 -0.5053165460

H 4.6562125768 -1.0177528520 -0.5796605460

H 0.1698215768 0.5095231480 -2.3422735460

H -0.9528464232 -0.2342548520 -1.6003635460

H -3.0536864232 2.2738751480 2.2708554540

H -2.6992364232 1.9350961480 0.7920874540

H 3.0471195768 0.1639251480 2.9987064540

H 1.6076005768 -0.3320008520 3.3121674540

H 2.4419335768 -0.6252978520 -2.4128225460

H 2.6264435768 0.8796951480 -2.2851885460

H 3.2540655768 2.2890281480 -0.5983175460

H 4.2995725768 2.7670801480 -1.6188115460

H 2.6268655768 -2.1111648520 -0.7272155460

H 3.4459805768 -2.9901578520 -1.7076105460

H 1.1785075768 -1.8035928520 1.2489434540

H 1.2437685768 -0.6443958520 0.2539844540

H -1.4967564232 1.9487651480 -1.0463145460

H 1.5674315768 1.0185941480 1.8399364540

H -0.4794744232 -1.4829238520 2.7357814540

H 0.3220305768 -2.5004408520 3.5945114540

H -3.9616784232 0.3640401480 -2.4967775460

H -2.5863594232 0.8646181480 -2.9393615460

H -4.3388064232 0.2894381480 1.2584504540

H -4.1049144232 -1.1721958520 1.5087754540

H -4.8883084232 -0.8914218520 -0.6162585460

H -5.8749334232   -1.0425398520   -1.7949175460

H -1.8471294232   -2.4055978520   -1.0845205460

H -0.4223834232   -2.0676428520   -0.4791535460

H -3.5800684232   -4.0183098520   -1.8413905460

H -3.8908894232   -2.5143128520   -1.9375885460

H -2.9250854232   2.2617881480   -1.3538395460

H -0.2221114232   3.5589281480   0.2111754540

H -1.7770354232   -1.2318278520   1.0394854540

H -2.1211674232   -0.0591078520   1.9782844540

H 1.3833745768   4.3310261480   -0.2941605460

57

I2_W18_TS

C -0.9201966759   3.4186969225   1.7749149772

C -0.2723356759   2.2559209225   2.1681669772

O -0.9704546759   1.2896509225   2.6276209772

O -1.7977356759   2.3781069225   -0.9214250228

O 0.2172233241   -0.6726710775   3.4981249772

O -2.9906476759   0.4489759225   0.4604559772

O -2.0310496759   2.0215969225   -3.6791980228

O 2.2641613241   2.1118859225   1.7190249772

O 2.6435213241   -0.8870080775   4.5644689772

O 4.0475003241   0.2930919225   2.6651919772

O 0.3554183241   1.1884339225   -2.1398060228

O 2.6019173241   0.8596499225   -0.5368920228

O -4.1946566759   -1.2180400775   -1.5048510228

O -3.3867016759   -0.5494590775   -3.9902400228

O -1.1884556759   -2.1803180775   -4.5403100228

O -0.0224716759   -1.5857050775   -2.1637760228

O -1.7628286759   -1.9710700775   -0.1009740228

O 0.1735843241   -2.6787120775   1.5756509772

O 2.0947343241   -1.8305010775   -0.3743290228

O 4.6981513241   -1.0684250775   0.1713249772

H 2.9579153241   -0.6168590775   5.4191599772

H 3.2518493241   -0.5006260775   3.9002689772

H 3.4281323241   0.9755719225   2.3485359772

H 4.3550843241   -0.1784560775   1.8884129772

H 1.2373273241   1.2553899225   -1.7568010228

H 0.1940233241   0.2383039225   -2.1963100228

H -2.9271396759   0.6770959225   1.3839669772

H -2.5777576759   1.1937539225   -0.0277230228

H 1.0754553241   -0.6832580775   3.9645179772

H 0.2246203241 -1.4151170775 2.8801219772

H 3.5399393241 0.6994959225 -0.6228090228

H 2.4855843241 1.3932909225 0.2910259772

H 1.0236483241 2.1825259225 2.0299599772

H 2.6255553241 2.9917959225 1.7773829772

H 3.9304273241 -1.6183960775 -0.0312900228

H 5.4728713241 -1.5907630775 -0.0038810228

H 1.5688463241 -2.1423470775 0.3698439772

H 2.0924053241 -0.8601190775 -0.3334660228

H -0.9445936759 2.0061639225 -1.1940290228

H -0.4061986759 0.5053879225 3.0118729772

H -0.6136676759 -2.5142950775 1.0144349772

H 0.1173683241 -3.5780040775 1.8820519772

H -2.4354426759 1.1706789225 -3.8617370228

H -1.0961796759 1.8443249225 -3.5734140228

H -4.1270526759 -0.5221840775 -0.8453670228

H -3.6142896759 -1.8964980775 -1.1562340228

H -3.7207726759 -0.7712100775 -3.0987750228

H -4.1536306759 -0.4571680775 -4.5463940228

H -0.2366496759 -1.9535800775 -3.0322910228

H 0.8198713241 -1.9228370775 -1.8408960228

H -1.4418426759 -2.9850210775 -4.9772180228

H -1.9877606759 -1.6435940775 -4.4579660228

H -2.2179346759 2.5175349225 -1.7769360228

H -1.8834416759 3.6886169225 2.1825129772

H -1.2099966759 -1.8268690775 -0.8857850228

H -2.0954446759 -1.0975860775 0.1527109772

H -0.4248196759 4.1094729225 1.1164289772

57

I2_W18_P

C 0.6456490405 1.6054309769 2.8011809799

C 0.9581420405 0.8239909769 1.6707259799

O 0.6019850405 -0.3497520231 1.5555459799

O -2.0824589595 2.3099699769 0.8292699799

O 0.8291030405 -2.8409560231 2.8463759799

O -3.2400809595 0.0125009769 1.4159719799

O -2.7683989595 3.1297929769 -1.7426350201

O 3.4873980405 2.7518049769 1.5924319799

O 3.4868710405 -2.2024070231 2.8260329799

O 4.1605770405 0.1605209769 1.5628499799

O -0.1605609595 2.0451569769 -1.1433710201

```
O 2.6182080405   2.2379369769   -1.0522030201
O -4.5437319595   -0.7630050231   -0.9582090201
O -4.0976939595   0.8608469769   -3.0465300201
O -1.9346309595   -0.3413510231   -4.3076280201
O -0.5851759595   -0.5905880231   -1.9670860201
O -2.0545469595   -2.0148370231   -0.1420880201
O -0.1689409595   -3.8621220231   0.3816289799
O 1.5154780405   -1.8086470231   -0.6241930201
O 3.7890230405   -0.4317390231   -1.2047770201
H 3.9161340405   -2.5650390231   3.5921469799
H 3.9398820405   -0.6313000231   2.0708569799
H 3.8003520405   1.8406889769   1.7310889799
H 4.2194530405   -0.1162940231   0.6460589799
H 0.7882680405   2.2269169769   -1.1899610201
H -0.2738339595   1.1440669769   -1.4696760201
H -3.5300849595   0.0203529769   2.3215029799
H -2.7956359595   0.8755719769   1.2509119799
H 2.5656330405   -2.5073580231   2.8549779799
H 0.5080140405   -3.3743590231   2.1136549799
H 3.0875180405   1.4192089769   -1.2257740201
H 2.9931580405   2.5900849769   -0.2380520201
H 1.5293670405   1.2932919769   0.8748539799
H 4.1857590405   3.3267589769   1.8842339799
H 3.0643830405   -1.0689580231   -1.1089680201
H 4.3935400405   -0.7863110231   -1.8475200201
H 1.2356000405   -2.7058960231   -0.4227310201
H 1.2367340405   -1.2954480231   0.1505939799
H -1.2572899595   2.1982989769   0.3384249799
H 0.4947980405   -1.9628570231   2.6577649799
H -0.9355179595   -3.2774220231   0.2207639799
H -0.4504169595   -4.7488310231   0.1844379799
H -3.1954999595   2.4370649769   -2.2499530201
H -1.8265229595   2.9712659769   -1.8378710201
H -4.3513039595   -0.3572110231   -0.1060980201
H -3.9379079595   -1.5055730231   -0.9716120201
H -4.3129099595   0.2829839769   -2.2866700201
H -4.9274689595   1.1085229769   -3.4410770201
H -0.8947769595   -0.6282320231   -2.8835920201
H 0.2425220405   -1.0701590231   -1.8675600201
H -2.2098009595   -0.9324150231   -4.9985520201
H -2.7307979595   0.1052839769   -3.9883330201
```

```
H -2.6262599595   2.7514069769   0.1660269799
H 0.0551250405   1.1823099769   3.5966699799
H -1.5869739595   -1.4593810231   -0.7866480201
H -2.3082349595   -1.4151140231   0.5694379799
H 0.9995740405   2.6193649769   2.8627239799
```

W33

102

w33_v1_R

```
O -0.9461512420   4.2076756350   -2.6725135072
H -1.6825892420   3.6001266350   -2.8246575072
H -1.2266312420   4.7950786350   -0.9274065072
O -6.0523332420   -0.1855383650   -0.7738065072
O -1.2560642420   -2.6640693650   0.4742264928
H -5.5573692420   1.3551656350   -1.1251655072
H -6.2295922420   -0.4020583650   0.1584224928
H -1.1426832420   -1.6852433650   0.5622054928
H -1.6344362420   -2.7683223650   -0.4171285072
O -0.8710532420   -0.0192323650   0.5470054928
O -5.2458202420   2.2834916350   -1.2776345072
H -0.1670922420   3.6512756350   -2.5063135072
H -1.2314542420   0.4500856350   1.3338784928
H -6.0111562420   2.8545576350   -1.1949995072
H -3.9827612420   2.4760456350   -2.4797085072
O -3.1849672420   2.4006896350   -3.0441625072
H -2.3444192420   1.0643366350   -2.2796315072
H -3.4920222420   2.3250876350   -3.9497405072
O -1.3432992420   4.7498836350   0.0396784928
H -1.9297922420   3.9788346350   0.1480864928
H 0.1692687580   4.2786106350   0.6174224928
O -2.8051772420   2.3825556350   0.1392304928
H -2.7560682420   2.0207536350   1.0424944928
H -3.7384082420   2.4148416350   -0.1124905072
O -1.9355552420   0.2731956350   -1.8715145072
H -2.3467122420   -1.3823823650   -2.0357855072
H -1.7180712420   0.4770166350   -0.9345025072
O -2.2263902420   1.1439726350   2.5327414928
H -2.8446812420   0.4654556350   2.8789854928
H -1.6886012420   1.4985976350   3.2678934928
O -5.0629942420   -2.7086923650   -1.7110355072
H -5.5332912420   -3.1392813650   -2.4274975072
H -5.6963422420   -0.9954123650   -1.1710035072
O -3.0742392420   -3.4044483650   2.3078794928
H -2.3584522420   -3.1894803650   1.6648784928
H -2.7307622420   -4.0761063650   2.8994564928
O -6.4434692420   -1.1242773650   1.8329324928
H -6.2253312420   -2.0561433650   1.5797154928
```

H -7.2913412420 -1.1402963650 2.2799674928

O -5.5323502420 -3.4585773650 0.9776034928

H -4.6761092420 -3.5918893650 1.4185304928

H -5.3392852420 -3.3404883650 0.0337914928

O -2.4149012420 -2.3637083650 -2.0890975072

H -4.1152402420 -2.6903603650 -1.9638035072

H -1.7513552420 -2.5994023650 -2.7503855072

O -3.9561612420 -0.8293253650 3.1899774928

H -4.8436742420 -0.7790953650 2.8027494928

H -3.6418852420 -1.7253113650 2.9956784928

H 0.0828747580 0.1593666350 0.4596294928

O -0.3225982420 2.0426876350 4.2607244928

H -0.2897932420 2.9572796350 4.5457554928

O 1.0560537580 3.8647636350 0.7717814928

H 1.4197027580 2.6095306350 1.8153944928

H 1.7369507580 4.5645006350 0.8231144928

O 4.7671487580 -1.1945283650 2.0554794928

O 2.2689587580 -2.8518633650 -2.1666575072

H 4.5397967580 -0.2630373650 2.2167514928

H 5.2798947580 -1.2152753650 1.2318054928

H 1.4787787580 -2.5543443650 -2.6551855072

H 1.9735687580 -2.9224933650 -1.2344645072

O 1.1965457580 2.6557706350 -1.6948415072

O 4.3461407580 1.5603546350 2.4115284928

H 1.2051027580 3.0430386350 -0.7951515072

H 2.1224447580 2.5940156350 -1.9776945072

H 4.8216227580 1.9610266350 3.1422394928

H 3.3920447580 1.7759466350 2.5389964928

O 1.6882617580 1.8414546350 2.3771404928

H 1.7022347580 1.0776166350 1.7686574928

H 0.4487237580 1.9207886350 3.6724594928

O 3.3389047580 5.3172616350 0.9022184928

H 3.9167667580 4.5992736350 0.5649244928

H 3.6095027580 6.1216526350 0.4590274928

O 4.7406337580 3.1462416350 0.0847924928

H 4.4788037580 2.7219436350 -0.7469505072

H 4.6747547580 2.4813526350 0.7884644928

O 1.8726377580 -0.1196733650 0.4764144928

H 1.8126297580 -1.0889283650 0.5861224928

H 2.5119647580 -0.0089423650 -0.2476805072

O 3.8459377580 2.0069606350 -2.3578725072

H 3.8264307580 1.0294436350 -2.2517315072

H 4.2956167580 2.1982196350 -3.1823945072

O 3.4866597580 -3.5063373650 2.1867844928

H 3.5820337580 -3.9099233650 3.0507594928

H 3.9066427580 -2.6098933650 2.2404634928

O 3.6646817580 -5.1840113650 -2.1808165072

H 3.1233087580 -4.3977793650 -2.3886245072

H 4.0359567580 -5.4969933650 -3.0060485072

O 5.9074167580 -1.7295853650 -0.4396815072

H 5.6699847580 -2.6930073650 -0.3559435072

H 6.8348347580 -1.6815673650 -0.6761585072

O 5.0966737580 -4.1950173650 -0.0509715072

H 4.6247867580 -4.6458613650 -0.7766845072

H 4.5075807580 -4.1839533650 0.7191974928

O 1.4993527580 -2.8497223650 0.4620874928

H 2.0156977580 -3.2791173650 1.1683394928

H 0.5451637580 -3.0092023650 0.5838424928

O 3.7257047580 -0.5487973650 -1.6198695072

H 4.5576487580 -0.9020353650 -1.2475425072

H 3.2481527580 -1.3277383650 -1.9691865072

C 0.0992007580 0.0184756350 -2.6252535072

H 0.4030337580 1.0060386350 -2.2905135072

C 0.1013897580 -1.1672593650 -3.0742535072

102

W33_V1_TS1

O -0.3822285611 0.3027797964 -4.6858116901

H -1.1887228655 -0.1497606709 -4.4053492979

H -0.7861686551 2.0860869777 -4.3252825175

O -5.9147531455 -0.6388926303 -0.5893082992

O -1.3785402993 -1.1647955028 2.4544000618

H -5.3781047835 -0.0442812315 -2.0409488415

H -6.1562657296 -0.0168358445 0.1195071181

H -1.2953686449 -0.4744315835 1.7552222565

H -1.7773010934 -1.9212533703 1.9891062278

O -1.0264083002 0.5748286939 0.4410812035

O -5.0382764673 0.3694418391 -2.8741835668

H 0.2815415220 0.1401987046 -3.9971665897

H -1.4219082872 1.4785277977 0.4860952654

H -5.7977876671 0.7110420807 -3.3487197856

H -3.6747309316 -0.4485453167 -3.6009237535

O -2.8308706340 -0.9257525372 -3.7512002300

H -2.1563366462 -0.9576627810 -2.1917367716

H -3.0527268593 -1.7714697319 -4.1470613074

O -0.9769819115 2.8838573110 -3.7996053042

H -1.6368686807 2.5656875158 -3.1578790204

H 0.5123455261 3.1257091731 -3.0225523137

O -2.6885411036 1.6495998601 -1.9519487584

H -2.7183411338 2.1759737960 -1.1326342735

H -3.6025842666 1.4756624331 -2.2147748161

O -1.7976154514 -1.1601631575 -1.2949199512

H -2.5510899372 -2.2897932049 -0.0629797166

H -1.5864173031 -0.3175985970 -0.7956707071

O -2.3485445954 2.8741896118 0.5028816187

H -2.9870186995 2.7456472660 1.2371983833

H -1.7752204687 3.6419244605 0.6987824313

O -5.2741080134 -2.9526798153 0.9751680759

H -5.7481014466 -3.7824246887 0.8942565916

H -5.7174648238 -1.4806134791 -0.1503267694

O -3.3058639307 -0.1053508137 4.0071037821

H -2.5458701812 -0.5137116360 3.5309041432

H -3.0482291526 -0.0199768709 4.9267745192

O -6.5301469483 0.8894340691 1.6685329186

H -6.3629413477 0.1369355375 2.2905323765

H -7.3993335605 1.2403513787 1.8694519996

O -5.7411968915 -1.1705808153 3.1244457509

H -4.8926563780 -0.9167575255 3.5259042838

H -5.5519336202 -1.8877951234 2.4988048429

O -2.6125424323 -2.9663077454 0.6375592907

H -4.3192138171 -3.1546529752 0.8738973095

H -1.9031798687 -3.5795011146 0.3756914251

O -4.1032412258 2.1576522917 2.4238814909

H -4.9722243561 1.8673128948 2.1057320734

H -3.8033320140 1.4606749516 3.0270443128

H -0.0518121308 0.6398304005 0.3333414454

O -0.3784867915 4.6942643291 0.9682851168

H -0.3033558636 5.5387056000 0.5216219547

O 1.3802519196 3.0209031067 -2.5598813232

H 1.5887606040 3.2013870121 -0.8882501887

H 2.0884446399 3.4469422384 -3.0835174733

O 4.7342340265 0.7616089463 2.2965279920

O 2.0486992919 -3.6674574044 0.8449594620

H 4.5457872005 1.4632264726 1.6511757100

H 5.2082552819 0.0578451455 1.8237909974

H 1.2590891057 -3.8360619411 0.2965218551

H 1.7879779587 -2.9147636920 1.4164002685

O 1.6103161951 0.3005550686 -2.6024909630

O 4.4058252492 2.8027500189 0.3924801701

H 1.6111362447 1.2833153147 -2.6178304299

H 2.4806841011 0.0050465589 -2.9213104154

H 4.9335977323 3.5860380639 0.5611667120

H 3.4739125842 3.1039293722 0.2932267109

O 1.7535092194 3.1692985663 0.0845792572

H 1.6819643072 2.2196558759 0.2994096241

H 0.4260724112 4.1915902012 0.7301365578

O 3.7225791703 3.8522934256 -3.6044581748

H 4.2454960925 3.1330767787 -3.1875635364

H 4.0507378454 3.9539038289 -4.4979783828

O 4.9483313203 1.8714560029 -2.2341426561

H 4.7477977197 0.9380218926 -2.4062165896

H 4.8043807000 2.0363184771 -1.2889022336

O 1.6417657782 0.4386875815 0.2312885612

H 1.8222922909 -0.2718411432 0.8735517930

H 1.8743472026 0.1110523806 -0.6538172827

O 4.1326105486 -0.7629649109 -2.8497648003

H 3.9834228448 -1.2888488538 -2.0247869831

H 4.5488609839 -1.3371769777 -3.4936341521

O 3.2846844807 -0.5434406018 4.0722644853

H 3.2482981519 -0.1028796812 4.9223209335

H 3.7843807280 0.0516430129 3.4557173056

O 3.3107718320 -5.0558484212 2.7995435861

H 2.8132895853 -4.7426768159 2.0174950365

H 3.6863350687 -5.9059179956 2.5690154200

O 5.7576745452 -1.6261028612 1.2992852462

H 5.4763447061 -2.1535574318 2.0948880976

H 6.6743568810 -1.8421089783 1.1227791695

O 4.8201160898 -2.8408831839 3.4318118526

H 4.3013668470 -3.6567973961 3.2951569129

H 4.2358086604 -2.1889689843 3.8478651789

O 1.4212170239 -1.4328001730 2.2886173284

H 1.9480929921 -1.1526000042 3.0604782554

H 0.4777891397 -1.3801024465 2.5241677959

O 3.6428942004 -1.8030982838 -0.4890887121

H 4.4108163008 -1.7441558842 0.1075633951

H 3.1179783527   -2.5603974721   -0.1716478930

C -0.2032284575   -2.0355345474   -1.4942802152

H 0.3443683645   -1.2892146437   -2.0522234754

C -0.2055636796   -3.1658059918   -0.8938165020

102

w33_v1_i

O -0.8811300916   -3.9663717532   2.4235583154

H -1.5911447962   -3.3993354149   2.7490030395

H -1.5119466478   -4.5466641183   0.7898874260

O -4.9267891533   1.1726878378   1.1215117110

O -0.9643699195   2.7851643601   -0.6450579092

H -5.2152534312   -0.4397954859   1.6135162201

H -5.2852399448   1.2826482852   0.2195813167

H -1.0356060260   1.8161075410   -0.7791982130

H -1.3946260031   2.9161474749   0.2239277267

O -1.0308793249   0.0584313720   -0.7365507383

O -5.2671227054   -1.4150593279   1.7463696156

H -0.1650074761   -3.3761928845   2.1342307175

H -1.5242368845   -0.3845494730   -1.4725689285

H -6.1923832784   -1.6404003614   1.8546297986

H -3.8977941461   -2.0155842008   2.7078991755

O -2.9870194364   -2.0955313525   3.0563965001

H -2.1752346220   -1.0941714192   1.9617119783

H -2.9903780773   -1.7091377832   3.9360168401

O -1.7699439240   -4.5345147502   -0.1505570580

H -2.3458110000   -3.7539181592   -0.2091538870

H -0.2388859429   -4.2944671518   -0.8754643315

O -3.1880078375   -2.0840872411   -0.0967600463

H -3.1853927182   -1.7545809925   -1.0137315851

H -4.0856529833   -1.9949661349   0.2469168970

O -1.7087655182   -0.2888846715   1.6256399939

H -2.8473794344   1.8139791075   1.5158652398

H -1.5153389364   -0.2977381445   0.6114991931

O -2.6026704291   -0.9939414478   -2.5682849375

H -3.0634798759   -0.1988197568   -2.9142426851

H -2.1001846757   -1.4191752153   -3.2931715168

O -4.6999761582   3.9012331516   1.8995524140

H -4.9230892587   4.5145993550   2.6013641864

H -5.1360703138   2.0064595786   1.5706714186

O -2.7376008257   3.8161656434   -2.3680823524

H -2.0068440126   3.5106908517   -1.7796066606

H -2.3614574400 4.4540405863 -2.9772883814

O -6.0183607060 1.8247042345 -1.3415337281

H -5.7931129261 2.7855216768 -1.2253011782

H -6.9455968324 1.7702537812 -1.5784860930

O -5.0766702686 4.2306680310 -0.8806193853

H -4.2222727090 4.2478305460 -1.3452526126

H -4.8942693779 4.2955589303 0.0708629677

O -2.3829097207 2.6378583083 1.7303095505

H -3.7590830432 3.6399066025 2.0185447188

H -1.6333915050 2.3446711256 2.3269819533

O -3.8943212718 1.3046864058 -3.1299724720

H -4.7031588184 1.3749737961 -2.5994172154

H -3.4503335501 2.1592028390 -3.0181120745

H -0.0706232407 -0.1734712126 -0.7639381306

O -0.8229718802 -2.0758617301 -4.3111640903

H -0.8745515278 -2.9813421069 -4.6214518854

O 0.6884556361 -4.0278362139 -1.0843832508

H 1.1508296286 -2.8188514308 -2.1705578795

H 1.2650864559 -4.8181842257 -1.1035838522

O 4.8898907538 0.6664728249 -1.9279933122

O 2.2756093278 2.3328600291 2.4600633150

H 4.5520576559 -0.2190410804 -2.1409774825

H 5.3322411780 0.6066745402 -1.0650731610

H 1.4021366491 2.1250110210 2.8595721887

H 2.0770916328 2.4353172386 1.5072607183

O 1.2277067406 -2.5351864869 1.1554204663

O 4.1338991119 -1.9734236412 -2.5652362683

H 1.1263379021 -3.1106105758 0.3672953410

H 2.0727832541 -2.7714772636 1.5752417832

H 4.6220868966 -2.3349877730 -3.3078456501

H 3.1785469022 -2.0871419883 -2.7707683229

O 1.4391470369 -2.0438982202 -2.7095390715

H 1.4835826986 -1.3159006535 -2.0606574063

H 0.0293535988 -2.0058650562 -3.8370389315

O 2.7641025385 -5.7424084899 -1.0899497820

H 3.4073047973 -5.0729550051 -0.7693885592

H 2.9498209577 -6.5543542294 -0.6180442656

O 4.3603462316 -3.7003160041 -0.3192136295

H 4.2588415771 -3.2750897318 0.5469512720

H 4.3499772472 -3.0034049536 -0.9939662187

O 1.5769522439 -0.3437369187 -0.5698771185

H 1.8905432643 0.5592378225 -0.3728028227
H 1.7224828922 -0.8965032904 0.2185919981
O 3.7738184147 -2.5702284799 2.1964631400
H 3.7495974620 -1.5805422713 2.1715723113
H 4.1286051485 -2.8364790712 3.0453038629
O 3.8385524054 3.0892127989 -1.9078051996
H 3.9179691153 3.5409418487 -2.7489134633
H 4.1937066927 2.1721271073 -2.0333743204
O 3.8787740966 4.5064822102 2.5679819351
H 3.2539258110 3.7677873791 2.7214703151
H 4.2493964463 4.7391079990 3.4195437939
O 5.9149905500 1.0575955217 0.6275516700
H 5.7806755579 2.0439035222 0.6133949617
H 6.8079590093 0.8957385886 0.9349307512
O 5.3617659411 3.6166395454 0.4297647815
H 4.8616671337 4.0163126591 1.1678181724
H 4.8126290178 3.6824426962 -0.3659842654
O 1.7862393462 2.3986798487 -0.2447451725
H 2.3984905570 2.8051388249 -0.8853802933
H 0.8829906551 2.7058249616 -0.4416132840
O 3.5980922691 0.0025038588 1.7367235381
H 4.4263788728 0.3638308009 1.3736070899
H 3.1412453613 0.7521954333 2.1633752753
C -0.4545693262 0.0071352770 2.4161188587
H 0.1915045324 -0.8666725614 2.3962779130
C -0.3212433036 1.2181274450 2.8998705255

102

W33_v1_TS2

O -0.7140730687 4.3677735572 -1.6464850448
H 0.1467041360 4.5809171945 -1.2638660818
H -0.3627147534 3.5914304429 -3.2769911917
O 5.0203098427 1.4370933139 0.1113616676
O 1.9744037283 -1.9038025918 1.3498456986
H 4.5190492890 2.7542164658 -0.7953960485
H 5.4646830609 0.7403855287 -0.4046672009
H 1.7493866793 -1.5050191462 0.4972004648
H 2.3786300529 -1.1167394070 1.8233386227
O 1.0528287632 -0.1436369376 -0.7396380407
O 4.1886217624 3.4219141138 -1.4459968212
H -1.0608210381 3.6019201276 -1.1603449609
H 1.4027235696 -0.4095842579 -1.6738657221

H 4.9619834999 3.8101328985 -1.8590178021

H 2.7948404819 4.3899847152 -0.9119542769

O 1.9288186479 4.6594791480 -0.5464858918

H 1.4289933994 2.9863958282 -0.1572439012

H 2.1094862408 5.1761968999 0.2416194874

O -0.1386814905 2.9241878064 -3.9524366037

H 0.7086473248 2.5663027444 -3.6373033239

H -1.4296908757 1.8462079383 -3.7622465794

O 2.1492184019 1.8877656668 -2.6968918862

H 2.4126960310 1.0062433934 -2.9954178834

H 2.9497387424 2.3748342875 -2.4543481275

O 1.2922613755 2.1103209576 0.2442960035

H 3.1310380825 0.7755547366 1.3867583270

H 1.2262588369 0.8449480579 -0.4860694763

O 2.0712920380 -0.8977773761 -2.8983184161

H 2.8112539548 -1.4848125057 -2.6106062954

H 1.4597989373 -1.3980375449 -3.4832440603

O 5.5145570040 0.4685857587 2.7203386075

H 5.8200004919 0.8001776850 3.5656532403

H 5.3567663308 1.3418243908 1.0177162740

O 4.0708166241 -3.4286488738 0.7127890887

H 3.2591304487 -2.9673352309 1.0336591537

H 3.9897011753 -4.3524223288 0.9559528442

O 6.3370226919 -0.7050383429 -1.0871802462

H 6.4513482553 -1.1559459608 -0.2068996677

H 7.1941425345 -0.6869841204 -1.5158039107

O 6.2918206775 -1.7973979891 1.2938705207

H 5.5466444518 -2.4199205986 1.2624105303

H 6.0574148951 -1.0992800534 1.9313988401

O 2.9775261808 0.3412419117 2.2382283770

H 4.5161471217 0.4157519532 2.7538867985

H 1.9555292797 0.8799314550 2.5853885841

O 4.1274953077 -2.2411812090 -1.8725068984

H 4.9194186148 -1.6910996062 -1.7512487303

H 4.0845694735 -2.7959094959 -1.0775937599

H 0.0524622463 -0.3235874006 -0.5729364287

O 0.1107439048 -2.1803170254 -4.2185656271

H -0.0861272357 -2.0633662018 -5.1491608786

O -2.1722184137 1.2897290982 -3.4187044864

H -2.0882097510 -0.3541391167 -3.0914108978

H -3.0138862411 1.5646029895 -3.8359650820

O -4.2691185920 -3.1494476395 0.6252226112

O -1.2348924334 -0.0128690313 3.8051034995

H -4.2996538912 -2.7084025090 -0.2396832021

H -4.7152108402 -2.5660624618 1.2614879841

H -0.5144322609 0.6294587789 3.6255983530

H -1.0020790670 -0.7759899115 3.2378316363

O -2.0633999095 2.0062090009 -0.7751890785

O -4.5295459770 -1.8701115346 -1.8684939557

H -2.2119802520 1.7929508204 -1.7217182057

H -2.9218114822 2.2860820187 -0.4121777865

H -5.1135201212 -2.3237463614 -2.4800110131

H -3.6781732554 -1.7311306738 -2.3402012671

O -2.0438058257 -1.2852170361 -2.7599686989

H -1.7917248902 -1.1844975273 -1.8233934710

H -0.7092520408 -1.9534411153 -3.7334006314

O -4.7503063989 1.7080612177 -4.0763298987

H -5.0920681392 1.4515011271 -3.1921724435

H -5.2345374511 2.4885927656 -4.3462935929

O -5.4399541041 0.8177604194 -1.6188892830

H -5.1730566356 1.2860586268 -0.8119007436

H -5.1448739743 -0.1021747322 -1.5360480441

O -1.3611470226 -0.5449674766 -0.1919329866

H -1.3826299820 -1.0041513357 0.6737977194

H -1.7643077861 0.3470326368 -0.1046439889

O -4.4343550577 2.2475100852 0.5931028478

H -4.0558174211 1.6199728956 1.2597702200

H -4.8528255085 2.9680977932 1.0653048587

O -2.3235729004 -4.2186876112 2.0564752473

H -2.1967965238 -5.1517881224 1.8797402046

H -3.0193701181 -3.8936325465 1.4295787847

O -1.8660565164 -1.5819632802 5.9157500953

H -1.5783123948 -0.8711109790 5.3075457793

H -2.0925513421 -1.1657061124 6.7475666465

O -5.0798327588 -1.6442507957 2.8162714610

H -4.5668453509 -2.1803642385 3.4799900714

H -5.9630590605 -1.5226536245 3.1672347922

O -3.5492191199 -3.1064516315 4.3671561409

H -2.9468869692 -2.6342312758 4.9742275522

H -3.0033044962 -3.6174999974 3.7510299161

O -0.7147799006 -2.0157293342 2.0128488959

H -1.1317143610 -2.8960268635 2.0668157942

H 0.2482219355   -2.1295861067   1.8959505135

O -3.3239764382   0.3563393754   2.0233895873

H -3.9615257569   -0.3202348717   2.3138905105

H -2.6344150282   0.3913774311   2.7136968080

C 0.4408091845   2.2031520769   1.3851661421

H -0.4780290018   2.7564264979   1.1875753683

C 0.7734749789   1.5805304225   2.4967373202

102

W33_v1_P

O -0.0033857400   3.7784127268   -2.7034080926

H -0.8244837400   3.2829677268   -2.8245430926

H -0.4156947400   4.7756017268   -1.1702430926

O -4.8213947400   0.1938077268   -0.7737650926

O -1.8825387400   -2.8545632732   0.6337219074

H -4.8274877400   1.8089027268   -1.2668340926

H -5.4374727400   0.0470247268   -0.0284500926

H -2.0722887400   -1.8903772732   0.6395849074

H -2.1296967400   -2.9156962732   -1.1906410926

O -2.2669737400   -0.2065112732   0.3964419074

O -4.6002657400   2.7464297268   -1.4713380926

H 0.6515002600   3.1420167268   -2.3742030926

H -2.4331287400   0.7149317268   1.9303599074

H -5.4154457400   3.2416477268   -1.5631760926

H -3.2344187400   2.6574997268   -2.5444230926

O -2.4701737400   2.3922887268   -3.0982540926

H -1.8420287400   0.8304467268   -2.6724260926

H -2.7537817400   2.4765147268   -4.0116010926

O -0.5582967400   5.0215207268   -0.2390300926

H -1.2643157400   4.4218287268   0.0622999074

H 0.8770362600   4.4215237268   0.4009239074

O -2.4586537400   3.0769057268   0.3876789074

H -2.6428807400   2.6292837268   1.2306909074

H -3.2940947400   3.1021157268   -0.0955030926

O -1.4444287400   -0.0644272732   -2.6387350926

H -2.0130247400   -1.7551822732   -2.2166320926

H -3.0328377400   0.0531027268   -0.1419910926

O -2.8125627400   1.3138817268   2.5998099074

H -3.9753917400   0.0313807268   3.1226329074

H -2.1081507400   1.5099427268   3.2506109074

O -4.9559147400   -2.2667172732   -2.0241940926

H -5.4031877400   -2.5741582732   -2.8145710926

H -4.9380257400 -0.5853462732 -1.3520150926

O -3.9200167400 -3.2124952732 2.4823119074

H -2.4815987400 -3.2179752732 1.3079929074

H -3.9935807400 -3.7803352732 3.2513249074

O -6.5806387400 -0.5695732732 1.2123989074

H -6.4785867400 -1.5319022732 0.9888339074

H -7.5168937400 -0.3848422732 1.2992699074

O -5.9158757400 -3.0196662732 0.5202199074

H -5.1998017400 -3.2763192732 1.1263939074

H -5.5312987400 -2.9399262732 -0.3679870926

O -2.3625037400 -2.6536952732 -2.1000550926

H -3.9894177400 -2.4838202732 -2.1302200926

H -0.0119467400 -2.0628042732 -4.1912170926

O -4.5749257400 -0.7379202732 3.1801509074

H -5.3482347400 -0.5084612732 2.6435649074

H -4.0850917400 -2.2857622732 2.8006539074

H -1.4422907400 0.1369117268 0.0007289074

O -0.6982207400 1.9088237268 4.2425589074

H -0.6758897400 2.7603317268 4.6813909074

O 1.6526232600 3.8558997268 0.6431469074

H 1.2838902600 2.6208097268 1.7394709074

H 2.4239322600 4.4177537268 0.8580289074

O 4.4021162600 -1.5310752732 2.5269299074

O 2.2007842600 -2.2601892732 -1.7726710926

H 4.1542822600 -0.5913242732 2.5796119074

H 5.1256932600 -1.6076792732 1.8837939074

H 1.6369972600 -1.7779662732 -2.3926220926

H 1.6411152600 -2.4419032732 -0.9868860926

O 1.8064002600 2.0239947268 -1.3759380926

O 3.7374042600 1.1549727268 2.6620289074

H 1.8712732600 2.6988247268 -0.6627090926

H 2.7206932600 1.8408737268 -1.6608120926

H 4.0067702600 1.5941147268 3.4720299074

H 2.7754432600 1.3516507268 2.5521369074

O 1.1588652600 1.7805357268 2.2424499074

H 0.7981302600 1.1597907268 1.5754159074

H 0.0354772600 1.9141547268 3.5943299074

O 4.0617872600 4.9077957268 1.2775629074

H 4.5561732600 4.1113907268 0.9836369074

H 4.5468752600 5.6718857268 0.9656919074

O 5.1008222600 2.5184687268 0.5907129074

H 4.9308832600 2.1545067268 -0.2923720926

H 4.6632802600 1.9307207268 1.2277059074

O 0.3776502600 0.2253637268 0.1116639074

H 0.6910912600 -0.6917802732 0.2203129074

H 0.9461832600 0.6926897268 -0.5326950926

O 4.4592532600 1.4289917268 -1.9472100926

H 4.6280632600 0.4545387268 -1.8797060926

H 4.9111562600 1.7371387268 -2.7347200926

O 2.7451702600 -3.5326212732 2.1305509074

H 2.6015802600 -4.0045552732 2.9529569074

H 3.3260082600 -2.7559112732 2.3484529074

O 4.1653582600 -4.1549642732 -2.3315090926

H 3.3305062600 -3.6596902732 -2.2968440926

H 4.8091122600 -3.4796192732 -2.5687570926

O 6.1391912600 -2.2898352732 0.4934539074

H 5.7344002600 -3.1980402732 0.4745639074

H 7.0899392600 -2.3960302732 0.5519419074

O 4.7548742600 -4.5252902732 0.3604759074

H 4.4778292600 -4.5749662732 -0.5740900926

H 3.9571032600 -4.3751782732 0.8914259074

O 0.8112472600 -2.4832952732 0.5129889074

H 1.3931782600 -2.8950592732 1.1817919074

H -0.1027247400 -2.8028942732 0.6496969074

O 4.7535192600 -1.1858822732 -1.6618300926

H 5.2816112600 -1.4944072732 -0.9031090926

H 3.8516462600 -1.5370762732 -1.5458570926

C -0.2116797400 -0.0362072732 -3.2367540926

H 0.3532832600 0.8886937268 -3.1079370926

C 0.2811972600 -1.0696492732 -3.8804960926

102

w33_v2_r

O 0.4867870788 2.7354654704 -3.0442582387

H -0.1694239212 2.0435974704 -3.2064952387

H -0.0947229212 4.0409934704 -1.8903442387

O -5.4427919212 -0.0095365296 -1.8904362387

O -3.2238129212 -1.1276325296 0.4114697613

H -4.3572989212 1.2432384704 -2.1435672387

H -6.1110899212 0.1106524704 -1.1952762387

H -2.5739549212 -0.3911495296 0.1957437613

H -3.1227449212 -1.7665695296 -0.3146532387

O -1.7105699212 0.8941904704 -0.1862552387

O -3.7606749212   1.9847524704   -2.4054322387

H 1.1951030788   2.3119804704   -2.5325992387

H -2.0261799212   1.5529814704   0.4637707613

H -4.2736119212   2.5246964704   -3.0114132387

H -2.3062999212   1.2249414704   -2.9916432387

O -1.5596369212   0.7934144704   -3.4643632387

H -0.7961089212   -0.5624875296   -2.6523522387

H -1.8217409212   0.7451794704   -4.3859622387

O -0.2564609212   4.6137794704   -1.1177372387

H -1.1879939212   4.4633934704   -0.8688272387

H 0.9660440788   4.2372174704   -0.0731332387

O -2.9028729212   3.9698864704   -0.5091332387

H -3.0036849212   3.6152844704   0.3869297613

H -3.1546599212   3.2371554704   -1.0910462387

O -0.3821069212   -1.2594695296   -2.1034042387

H -1.5806139212   -2.4540875296   -1.7459752387

H -0.0408249212   -0.7825085296   -1.3258362387

O -2.9264589212   2.3691254704   1.7981337613

H -3.6841949212   1.8122274704   2.0382227613

H -2.3303799212   2.4361754704   2.5711297613

O -4.9712109212   -2.8397205296   -1.7304802387

H -5.4087629212   -3.3837055296   -2.3886512387

H -5.2280879212   -0.9547015296   -1.8898832387

O -5.2576309212   -1.9941715296   3.1311137613

H -5.6293149212   -2.1021535296   4.0078897613

H -5.2405539212   -1.0351495296   2.9337857613

O -7.1900179212   0.0373144704   0.2703077613

H -7.1029859212   -0.9345565296   0.4337687613

H -8.1242849212   0.2476234704   0.2312727613

O -6.5270589212   -2.4998115296   0.6486817613

H -6.0644079212   -2.5621615296   1.5002077613

H -5.8977879212   -2.7477325296   -0.0464972387

O -2.2977219212   -3.0505985296   -1.4259022387

H -4.0193729212   -3.0645225296   -1.7643352387

H -1.8956639212   -3.5811775296   -0.7287322387

O -4.9724539212   0.4084844704   1.8730857613

H -5.8177629212   0.6012274704   1.4349447613

H -4.4834679212   -0.1224275296   1.2168387613

H -0.7677569212   0.7494594704   -0.0073492387

O -0.9862499212   2.4885374704   3.7118407613

H -0.8008279212   3.3115654704   4.1665037613

O 1.7162120788 3.8229344704 0.4306697613

H 1.3595040788 2.6776644704 1.6004097613

H 2.4234120788 4.4765754704 0.5918117613

O 4.9055970788 -1.2383145296 2.4041267613

O 2.1693400788 -2.4669035296 -1.8890652387

H 4.5483980788 -0.3344365296 2.4156067613

H 5.4613520788 -1.3390635296 1.6138827613

H 1.3750220788 -2.1141185296 -2.3183972387

H 1.9117880788 -2.5836075296 -0.9510842387

O 2.4205330788 1.7981384704 -1.2321582387

O 3.8965600788 1.3675154704 2.5500267613

H 2.3357540788 2.5865974704 -0.6517552387

H 3.3386750788 1.7484584704 -1.5535982387

H 4.2006140788 1.8364214704 3.3306347613

H 2.9287890788 1.5381114704 2.4907827613

O 1.2444140788 1.8880534704 2.1865687613

H 1.0155200788 1.1692474704 1.5699367613

H -0.1609519212 2.2489144704 3.2445757613

O 4.0795270788 5.0497174704 0.9274707613

H 4.6208810788 4.2679414704 0.6878307613

H 4.5281620788 5.8194864704 0.5774027613

O 5.2893740788 2.6695054704 0.4549787613

H 5.3221510788 2.1940774704 -0.3890352387

H 4.8234560788 2.0984394704 1.0859147613

O 0.9159210788 0.0578534704 0.0765707613

H 1.2803270788 -0.7921515296 0.4052497613

H 1.6055480788 0.5444634704 -0.4343102387

O 5.0106390788 1.3525414704 -2.0383452387

H 4.9443600788 0.3653244704 -2.0932042387

H 5.3758090788 1.6611254704 -2.8689912387

O 3.3565820788 -3.3463835296 2.5163647613

H 3.2258430788 -3.6863785296 3.4025867613

H 3.9330130788 -2.5409665296 2.5856297613

O 4.1386520788 -4.3858495296 -2.1487262387

H 3.3109890788 -3.8805675296 -2.2193432387

H 4.7892500788 -3.8058235296 -2.5553692387

O 6.2168290788 -2.1758955296 0.1447757613

H 5.8740060788 -3.0959005296 0.3076587613

H 7.1699200788 -2.2350795296 0.0593207613

O 5.0057070788 -4.4804845296 0.4930067613

H 4.5755340788 -4.6156495296 -0.3719782387

H 4.3155420788 -4.3255505296 1.1557497613

O 1.4683930788 -2.4865415296 0.7339987613

H 2.0726470788 -2.8307445296 1.4213157613

H 0.5486920788 -2.7091875296 1.0077907613

O 4.7104280788 -1.2772845296 -2.0205492387

H 5.1988740788 -1.6137235296 -1.2472862387

H 3.7905540788 -1.5903055296 -1.9483472387

C -2.4161789212 -2.4095845296 1.9533407613

H -3.3180469212 -2.3424365296 2.5417677613

C -1.2806639212 -2.7094025296 1.4839467613

102

w33_v2_ts1

O 2.4584613790 -2.3499408377 -2.4891981584

H 1.9179356287 -2.8893152484 -1.8961154551

H 1.5931441277 -1.6093122729 -3.9326188839

O -3.6181803148 -4.0958752848 -0.6114400737

O -3.1590509564 -1.1798438255 0.6959674057

H -2.3026622982 -3.9402975805 -1.6446685919

H -4.5276825794 -3.9387678047 -0.9148495984

H -2.4008919880 -1.0149230416 0.0024652285

H -2.7744213961 -1.8338112380 1.3188310020

O -1.3899988376 -0.8930927403 -1.0438128393

O -1.5402380024 -3.9419550155 -2.2692276726

H 2.7022676759 -1.5599813682 -1.9792251029

H -1.8364020728 -0.4643416546 -1.8058653524

H -1.6975444581 -4.6745654792 -2.8695195672

H -0.0880561591 -3.9153152168 -1.3523101673

O 0.7359850021 -3.9024622380 -0.8142746341

H 0.6802755122 -2.8018884301 0.5726991635

H 1.0186492955 -4.8143857416 -0.7223114005

O 1.1459183056 -1.0065272572 -4.5551612688

H 0.2039228362 -1.2579423843 -4.5261801590

H 1.5778913723 0.5085316655 -4.0468056295

O -1.5021791771 -1.8188345878 -4.2171425788

H -2.1157850190 -1.1104848830 -3.9726954935

H -1.5034220909 -2.4254378763 -3.4619398786

O 0.5762435935 -2.1246036779 1.2701361062

H -0.8533686740 -2.4199504130 2.2027286361

H 0.5461203784 -1.2755028677 0.7932528809

O -3.0282964902 0.1696904723 -2.9172412397

H -3.8944557259 -0.0034751608 -2.5152316397

```
H -2.8931532628   1.1379349478   -2.9795991215
O -3.8647792007   -4.0405555140   2.2302388476
H -4.0086831201   -4.8195127540   2.7713328560
H -3.6626502390   -4.1781148599   0.3532388005
O -6.4338369377   -0.0000557909   0.9709761459
H -7.1736622935   0.6081347959   1.0009057436
H -6.1594861553   -0.0844745427   0.0387542651
O -6.2412703032   -3.3636470172   -1.1409853094
H -6.4193604068   -3.2076954459   -0.1795454627
H -6.9572633564   -3.8940957131   -1.4935865747
O -6.3137043851   -2.7689055081   1.4324492172
H -6.3790332291   -1.8016785116   1.5078363083
H -5.4838345530   -3.0420401056   1.8519837100
O -1.7305810398   -2.4475430579   2.6529748022
H -3.0593043557   -3.6015332597   2.5698690477
H -1.7755349184   -1.6217344884   3.1547791325
O -5.1180126096   -0.8474373015   -1.2961603384
H -5.5971641557   -1.6434018641   -1.5808153925
H -4.4851210832   -1.1853313970   -0.6410572796
H -0.6424870509   -0.3310742906   -0.7698105026
O -2.2720406670   2.7758428678   -2.9262917165
H -2.1674051467   3.2690382388   -3.7413341854
O 1.8750359159   1.3143424883   -3.5471117371
H 0.7866529445   2.0978392558   -2.5420669331
H 2.5040978489   1.8357687836   -4.0824435808
O 2.7458439602   4.5388282174   1.8090395992
O 2.3730572048   -0.6150660578   2.8826272907
H 2.5855981137   4.3720339481   0.8654856582
H 3.5887754066   4.1234562869   2.0547266271
H 1.9822573640   -1.3846265317   2.4425031326
H 1.6642752156   0.0644409787   2.8785301886
O 2.9491196813   0.1632209466   -1.3392179438
O 2.2315742297   4.1844819401   -0.9235181597
H 2.7256072691   0.6515681873   -2.1621407005
H 3.8748836679   0.3604349085   -1.1066636454
H 2.1654794533   5.0231536941   -1.3860083008
H 1.4700352635   3.6436531754   -1.2321317002
O 0.2557091549   2.5253623829   -1.8240491241
H 0.2435595821   1.8626616826   -1.1109994936
H -1.3906074271   2.7684920664   -2.5018307890
O 3.8261677973   2.9796456305   -4.4095584204
```

H 4.2744154572 3.0246082881 -3.5381917874

H 4.5116743366 2.9262749321 -5.0755890299

O 4.6845307943 3.1147551797 -1.8449223449

H 5.0497294626 2.3900549281 -1.3147044527

H 3.8771141323 3.4156539911 -1.3989318037

O 0.7143114100 0.4589786672 0.0541186696

H 0.7237854765 0.9139068835 0.9274658952

H 1.6330963477 0.3887957519 -0.2992792184

O 5.4613420903 0.7913851005 -0.4256259197

H 5.2745379011 0.6950603285 0.5437133578

H 6.2430369781 0.2714713967 -0.6186127454

O 1.0136149094 3.7762815250 3.6269002114

H 0.3649205650 4.4409415192 3.8624621131

H 1.5996591560 4.1617141997 2.9258216747

O 3.8174501491 0.1272181087 5.1141580316

H 3.2440429800 -0.3400622807 4.4827718646

H 4.6779759072 0.1174568965 4.6850994651

O 4.8112140703 3.2054853744 3.1046857348

H 4.2765545351 3.1501210388 3.9429801236

H 5.6503409530 3.6126789960 3.3265991245

O 3.2133116078 2.8440824618 5.1554138126

H 3.2549575677 1.8779272757 5.2815291547

H 2.3237496364 3.0727796993 4.8445747754

O 0.4845667873 1.2901734701 2.5859269947

H 0.5927512004 2.1816946284 2.9698689025

H -0.4803347565 1.0544082928 2.6430763318

O 4.7835016818 0.6006654535 2.1204083106

H 4.7500293191 1.5049528308 2.4825224209

H 3.9187947566 0.1826610034 2.2863865453

C -3.2984933335 0.2469602986 1.7157069454

H -4.3454081480 0.4710414771 1.5741223442

C -2.2077654916 0.5427427084 2.3202281715

102

w33_v2_i

O 0.5961992498 2.8581665033 -3.1776280210

H -0.0314127502 2.1928795033 -3.4911150210

H 0.0108102498 4.1262875033 -1.9956960210

O -5.0156407502 -0.0915954967 -1.6260360210

O -3.2412737502 -1.1900814967 0.6326519790

H -4.0769237502 1.1167335033 -2.2656690210

H -5.7410767502 0.1221535033 -1.0141670210

H -2.3020047502 -0.0791074967 0.2426069790

H -3.3328877502 -1.6648184967 -0.2071570210

O -1.7347807502 0.7562565033 0.0570069790

O -3.5459307502 1.8647195033 -2.6329880210

H 1.2456322498 2.3775225033 -2.6386040210

H -2.0257857502 1.4415335033 0.7610599790

H -4.1648657502 2.4093365033 -3.1252370210

H -2.1675467502 1.2571045033 -3.4764390210

O -1.3546337502 0.8978625033 -3.8966440210

H -0.6508757502 -0.4689514967 -2.9672970210

H -1.5459157502 0.7949985033 -4.8308820210

O -0.1770467502 4.6549555033 -1.1976570210

H -1.1043147502 4.4586125033 -0.9731920210

H 1.0261252498 4.2633235033 -0.1199620210

O -2.7894207502 3.8189475033 -0.6542140210

H -2.9067837502 3.4844095033 0.2449769790

H -2.9858487502 3.0740155033 -1.2418290210

O -0.3366767502 -1.1658734967 -2.3608390210

H -1.6701297502 -2.3462214967 -1.9271230210

H -0.0342137502 -0.6973884967 -1.5687510210

O -2.7311257502 2.3175595033 1.7796419790

H -3.5209007502 1.8292425033 2.0871279790

H -2.1534237502 2.5402605033 2.5464929790

O -4.9717307502 -2.9075684967 -1.6410100210

H -5.4056507502 -3.5523134967 -2.2028260210

H -5.0801227502 -1.0430614967 -1.8066300210

O -5.4559447502 -1.8961934967 3.2353629790

H -5.7454217502 -2.0474614967 4.1356329790

H -5.2742447502 -0.9458624967 3.1401779790

O -6.9698177502 0.2011225033 0.3249409790

H -6.9571727502 -0.7782134967 0.4878389790

H -7.8833257502 0.4703345033 0.2185239790

O -6.5364187502 -2.3605124967 0.7131189790

H -6.1309637502 -2.4347944967 1.5946069790

H -5.8964827502 -2.6885324967 0.0632279790

O -2.3142017502 -2.9264284967 -1.4778130210

H -4.0053707502 -3.0854954967 -1.6892750210

H -1.8660617502 -3.1691224967 -0.6463600210

O -4.8163087502 0.5395425033 2.0336039790

H -5.6509407502 0.7308585033 1.5739479790

H -4.3737087502 -0.1057554967 1.4463549790

H	-0.7508577502	0.5306105033	0.1132269790
O	-0.8729297502	2.8009825033	3.6367169790
H	-0.6829977502	3.6838155033	3.9582789790
O	1.7608012498	3.8417235033	0.3971599790
H	1.4509362498	2.7101095033	1.5847319790
H	2.4925242498	4.4772515033	0.5296639790
O	4.8300522498	-1.3265234967	2.5507059790
O	2.1375292498	-2.5437604967	-1.9442750210
H	4.5305382498	-0.4037664967	2.5193759790
H	5.3632952498	-1.5008024967	1.7571609790
H	1.3798222498	-2.1918304967	-2.4336000210
H	1.8394482498	-2.5694784967	-1.0054160210
O	2.3334872498	1.7751825033	-1.2606690210
O	3.9579622498	1.3465735033	2.6519959790
H	2.3060632498	2.5728515033	-0.6875590210
H	3.2611252498	1.6255515033	-1.5290600210
H	4.2565082498	1.7609635033	3.4647279790
H	2.9999632498	1.5505285033	2.5761889790
O	1.3240742498	1.9492815033	2.2067719790
H	1.0589112498	1.2122515033	1.6308139790
H	-0.0434867502	2.4722955033	3.2333019790
O	4.1343122498	5.0370295033	0.8371129790
H	4.6570462498	4.2271405033	0.6514679790
H	4.6031852498	5.7721215033	0.4419879790
O	5.2842312498	2.6163685033	0.4966819790
H	5.2783382498	2.0907875033	-0.3185720210
H	4.8722322498	2.0739515033	1.1874669790
O	0.7517072498	0.1451415033	0.0740539790
H	1.0966802498	-0.7474824967	0.3532669790
H	1.4328342498	0.6372885033	-0.4519590210
O	4.9210182498	1.1946255033	-1.9144890210
H	4.8832452498	0.2004075033	-1.9446220210
H	5.3207362498	1.4849655033	-2.7360490210
O	3.0800212498	-3.2975094967	2.5403269790
H	2.7771672498	-3.5788384967	3.4049039790
H	3.7164062498	-2.5500834967	2.6708009790
O	3.7063532498	-4.7984604967	-1.9793730210
H	3.0516712498	-4.0899994967	-2.1195120210
H	4.4306302498	-4.5894394967	-2.5725540210
O	6.0452032498	-2.4319044967	0.2949019790
H	5.6074392498	-3.3156474967	0.4384009790

H 6.9894952498 -2.5903654967 0.2454689790

O 4.6543132498 -4.6306554967 0.6241829790

H 4.2119912498 -4.8117944967 -0.2261950210

H 3.9732722498 -4.4072024967 1.2775389790

O 1.3555132498 -2.3454464967 0.6090989790

H 1.9204362498 -2.6898914967 1.3267579790

H 0.3913012498 -2.5519184967 0.8571789790

O 4.7027432498 -1.4181474967 -1.9280410210

H 5.1579162498 -1.8165224967 -1.1644520210

H 3.7919802498 -1.7644894967 -1.9434580210

C -2.4962137502 -2.0732774967 1.5710079790

H -3.1216127502 -2.2508744967 2.4441359790

C -1.2922977502 -2.4914574967 1.2645339790

102

w33_v2_ts2

O 0.5776356151 2.9217921527 -3.1452364741

H -0.0501423849 2.2423231527 -3.4246054741

H 0.0511566151 4.1836881527 -1.9257264741

O -4.9562913849 0.1248711527 -1.4837794741

O -3.1357833849 -1.2459198473 0.6010885259

H -4.0523373849 1.3355511527 -2.1595424741

H -5.6980293849 0.3297091527 -0.8892744741

H -2.2111253849 -0.0292598473 0.2124665259

H -3.1544093849 -1.7249248473 -0.2451894741

O -1.6795893849 0.8123171527 0.0390865259

O -3.5042203849 2.0636641527 -2.5409464741

H 1.2622746151 2.4530001527 -2.6387854741

H -1.9600283849 1.4676521527 0.7721855259

H -4.1087303849 2.6276341527 -3.0290054741

H -2.1744393849 1.3603971527 -3.3824714741

O -1.3905293849 0.9416271527 -3.8028324741

H -0.7411413849 -0.4616018473 -2.9038944741

H -1.5814263849 0.8707581527 -4.7401554741

O -0.0911233849 4.7134881527 -1.1191504741

H -1.0123643849 4.5391481527 -0.8564014741

H 1.1479726151 4.2920331527 -0.0841954741

O -2.7054573849 3.9302131527 -0.4968474741

H -2.8126913849 3.5507901527 0.3856155259

H -2.9195363849 3.2183521527 -1.1182084741

O -0.4272213849 -1.1785848473 -2.3199034741

H -1.6753063849 -2.3316758473 -1.8816964741

H -0.0275233849 -0.7295638473 -1.5590494741

O -2.6338423849 2.3011831527 1.8551515259

H -3.4051223849 1.7859511527 2.1620575259

H -2.0300893849 2.4854541527 2.6127675259

O -5.0370123849 -2.6903758473 -1.7443364741

H -5.5031753849 -3.2211478473 -2.3931264741

H -5.0398743849 -0.8141698473 -1.7130324741

O -5.4026623849 -1.9939648473 3.1386855259

H -5.6920093849 -2.1660098473 4.0354365259

H -5.1759903849 -1.0496358473 3.0796035259

O -6.9356313849 0.3527791527 0.4386865259

H -6.9702563849 -0.6346218473 0.5294445259

H -7.8347323849 0.6795301527 0.3842955259

O -6.5954433849 -2.2462608473 0.6403965259

H -6.1755523849 -2.3859618473 1.5074225259

H -5.9749743849 -2.5508908473 -0.0385324741

O -2.3682483849 -2.9161588473 -1.5013524741

H -4.0927663849 -2.9478178473 -1.7873434741

H -1.9240533849 -3.3995838473 -0.7926614741

O -4.6927123849 0.4740161527 2.0541215259

H -5.5354983849 0.7155451527 1.6342065259

H -4.2759893849 -0.1344598473 1.4130505259

H -0.6666553849 0.5978041527 0.0559815259

O -0.7240983849 2.6994641527 3.6759685259

H -0.5285563849 3.5679861527 4.0312605259

O 1.8877446151 3.8509821527 0.4067795259

H 1.5700936151 2.6920801527 1.5707865259

H 2.6367406151 4.4692331527 0.5213545259

O 4.8634726151 -1.3845268473 2.5174375259

O 2.0512726151 -2.6480768473 -1.9613614741

H 4.6102756151 -0.4494738473 2.4607785259

H 5.3525236151 -1.6166718473 1.7104405259

H 1.3219466151 -2.2975018473 -2.4883184741

H 1.7420136151 -2.5548098473 -1.0168964741

O 2.3736646151 1.8112421527 -1.3214864741

O 4.0708256151 1.3120121527 2.5882565259

H 2.3903946151 2.5896461527 -0.7237404741

H 3.2915316151 1.6103411527 -1.5895054741

H 4.3726346151 1.7261271527 3.3997515259

H 3.1105006151 1.5080961527 2.5197505259

O 1.4358446151 1.9148951527 2.1697925259

H 1.1524976151   1.2005791527   1.5716565259

H 0.0955656151   2.3935941527   3.2340145259

O 4.3052896151   4.9901791527   0.7623045259

H 4.7952406151   4.1633951527   0.5613845259

H 4.7762206151   5.7065751527   0.3365255259

O 5.3706096151   2.5358701527   0.3983375259

H 5.3335916151   1.9931541527   -0.4049444741

H 4.9676256151   2.0140611527   1.1103075259

O 0.7448066151   0.2015321527   0.0208565259

H 1.0631476151   -0.7534798473   0.2794905259

H 1.4196806151   0.6861251527   -0.5147934741

O 4.9204596151   1.0706901527   -1.9697904741

H 4.8360266151   0.0777771527   -1.9769114741

H 5.3399816151   1.3185901527   -2.7953044741

O 2.9489446151   -3.2075128473   2.4914415259

H 2.5790296151   -3.4240768473   3.3483355259

H 3.6395216151   -2.5132718473   2.6292805259

O 3.1778086151   -5.1316458473   -1.8320014741

H 2.7068036151   -4.2958828473   -2.0182474741

H 3.8128476151   -5.2369098473   -2.5419564741

O 5.9190806151   -2.6349738473   0.2440025259

H 5.4103456151   -3.4791848473   0.3931885259

H 6.8452246151   -2.8754558473   0.1843665259

O 4.3799836151   -4.7240588473   0.6361925259

H 3.8890726151   -4.9581268473   -0.1737274741

H 3.7365436151   -4.4152618473   1.2945395259

O 1.2272126151   -2.1974838473   0.4909295259

H 1.8302316151   -2.5127918473   1.1851075259

H 0.1079796151   -2.4006578473   0.8971655259

O 4.6204206151   -1.5295548473   -1.9599004741

H 5.0754836151   -1.9550768473   -1.2112134741

H 3.7150236151   -1.8897158473   -1.9929774741

C -2.4325163849   -2.1080808473   1.5556545259

H -3.1067913849   -2.3817848473   2.3652765259

C -1.1819393849   -2.4355588473   1.3677035259

102

w33_v2_p

O 0.0143710332   2.1204529352   -4.0294215080

H -0.8046454911   1.6263960746   -3.8757266366

H -0.0085582719   3.5463163065   -2.9132889073

O -4.8095614365   0.9122905101   0.3292301570

O -1.9027776043 0.3505154372 1.6627168273
H -4.1931769613 1.8944896804 -0.9268660721
H -5.4843457100 1.2434553286 0.9455764570
H -1.4621857189 2.1953672503 0.0998969945
H -2.0191151776 -0.5064325679 1.2023087787
O -1.1145301159 1.8078014477 -0.7236098725
O -3.9663529349 2.4560086928 -1.7038201973
H 0.7233166792 1.5762661090 -3.6478952739
H -0.6744939058 2.5526383510 -1.1749305873
H -4.8125495031 2.7261208683 -2.0677618358
H -2.8464590100 1.4271020797 -2.6872097086
O -2.4398688578 0.8414204805 -3.3605108834
H -1.7123548319 -0.6379775751 -2.6377163007
H -3.0807149692 0.7802042932 -4.0719432391
O 0.0882533276 3.9931042042 -2.0485941774
H -0.7329719362 4.4840586241 -1.8453461315
H 1.6552641580 3.6641175309 -1.3390990585
O -2.3387753172 4.7590701923 -1.0393164211
H -2.1449934027 4.5800690548 -0.1040772615
H -2.9139426084 4.0212222303 -1.2937692815
O -1.2414012595 -1.3297811673 -2.1350445955
H -2.0541085786 -1.8140208014 -0.6284586749
H -0.5402861828 -0.8515476944 -1.6616665683
O -1.7283087596 3.5581535302 1.4241977515
H -2.4254475533 3.2862050983 2.0449593319
H -0.8983238210 3.5436488509 1.9340978312
O -4.7832189402 -1.8521705364 0.9741859657
H -5.3349347899 -2.5418524792 0.6003330093
H -4.8305931884 -0.0542973155 0.4135380799
O -3.5306393050 0.1535739784 4.9276368790
H -2.6694588979 -0.2686770080 4.8509429879
H -3.4544700180 0.9621677546 4.3895442928
O -6.3075690509 1.6183797094 2.5311413503
H -6.2094226203 0.7243590632 2.9533028634
H -7.1726266513 1.9616843122 2.7571943189
O -5.6749813981 -0.7595196283 3.4535357417
H -4.9301610767 -0.6059120632 4.0666034585
H -5.3101272663 -1.2501373127 2.7012469126
O -2.1964631545 -2.0403647721 0.3162073384
H -3.8534467787 -2.1043435638 0.7899960851
H -1.3724771505 -2.5051173619 0.5574587833

O	-3.5984254215	2.1386220404	2.9104529881
H	-4.5594635972	2.2575326493	2.8438062039
H	-3.3777793052	1.4824320457	2.2270769977
H	-0.0313643893	0.6297853752	-0.4232952181
O	0.5901566423	3.1026445740	2.8719998368
H	1.0955793564	3.7450968953	3.3717225016
O	2.4152760138	3.1303749043	-1.0287815748
H	2.2882655211	2.2460851294	0.4274507142
H	3.2583597756	3.5854620102	-1.2249375143
O	4.4269934815	-2.3964322940	1.4230825036
O	0.6187214154	-3.4593738543	-2.0873972021
H	4.4947424485	-1.4417175793	1.2540100107
H	4.6943971991	-2.8766248757	0.6215144825
H	-0.0722522570	-2.9015278130	-2.4763028395
H	0.6034032568	-3.2212895365	-1.1356620880
O	2.0450761351	0.8647013382	-2.5172434005
O	4.6124069339	0.3761644016	1.1249819807
H	2.3803453104	1.6759724435	-2.0839970337
H	2.8025764817	0.3864712928	-2.9039382972
H	5.2268203660	0.7611269510	1.7537620409
H	3.7599113650	0.8571112142	1.2375719567
O	2.2120950155	1.6295472752	1.1944735020
H	1.6368368173	0.9159638819	0.8614980570
H	1.2340772749	2.5829681690	2.3499331610
O	4.9976949219	3.6775799920	-1.4917878462
H	5.2331897083	2.7305112835	-1.5956632454
H	5.5169584288	4.1698970443	-2.1274233508
O	5.3948681460	1.0046506701	-1.5210935594
H	5.0630437406	0.3552227797	-2.1609820451
H	5.1529705351	0.6835149898	-0.6381201130
O	0.6700002086	-0.0777580412	-0.4086816664
H	0.6565826220	-1.6689240036	0.3560382459
H	1.2591441927	0.1437943779	-1.1659350205
O	4.1369990493	-0.6858438753	-3.3965314159
H	3.8469658749	-1.6100290454	-3.1715581611
H	4.4377347783	-0.7095989107	-4.3066338762
O	2.2672827495	-3.5934334143	2.2777318029
H	2.2140621813	-3.6303919690	3.2340280372
H	3.0973483981	-3.0970562532	2.0466534831
O	0.8225776054	-6.1133728190	-1.4681049763
H	0.6433085343	-5.2458171868	-1.8764338094

```
H 1.0030208764   -6.7217321429   -2.1857906023
O 4.7124199065   -4.2352805637   -0.6525581488
H 4.0403738884   -4.8525857670   -0.2584264031
H 5.5097408259   -4.7471738044   -0.7991731883
O 2.7895292868   -5.6726974616   0.4287201895
H 2.0508273224   -5.8846772022   -0.1725913688
H 2.4370036949   -5.1411184799   1.1577994781
O 0.4563487542   -2.6159627983   0.4957359756
H 1.0625552064   -2.9518890841   1.1850106246
H -0.4000710837   -1.9180624239   2.9383934983
O 3.3192578418   -3.1184655385   -2.7959197589
H 3.8138701556   -3.5454125244   -2.0747722297
H 2.3724495986   -3.2358578984   -2.6028552435
C -1.0036747882   0.2438374051   2.6885945422
H -0.7781271372   1.2174371928   3.1233600805
C -0.4479193868   -0.8585391081   3.1393191671
```
